# Geographic determinants of goods vehicle speeding in British cities

**DOI:** 10.1038/s44333-026-00106-3

**Published:** 2026-06-03

**Authors:** Long Chen, Ed Manley

**Affiliations:** School of Geography, University of Leeds, Leeds, UK

**Keywords:** Complex networks, Complex networks, Engineering, Environmental social sciences, Geography, Geography

## Abstract

Vehicular speeding remains a major challenge in urban and transportation planning, influenced by numerous underlying factors. Yet the influence of urban context in determining driving speed is less well understood. In this paper we undertake a large-scale, comprehensive analysis of speeding behaviour across 13 British cities, using trajectory data from goods vehicles across 3.2 million journeys. Unlike prior studies, we assess determinants of speeding across multiple scales—from engineering interventions to regional measures of road network configuration. The results indicate that road geometry and network connectivity have a causal association with speeding, similar to the effect of engineering interventions. These findings have two clear implications for urban and transportation planning. First, the credibility of speed reduction efforts can be enhanced through considering road network context, alongside engineering interventions. Second, analyses of local spatial behaviours should incorporate measures of urban context, reflecting the nature of human spatial perception.

## Introduction

Vehicular speeding remains a concern for cities and their citizens. Speeding, characterised by vehicle drivers exceeding the legal speed limit, poses a threat to road safety^1,2^ and exacerbates urban environmental challenges relating to emissions and noise pollution^3–7^. Addressing speeding behaviour can encourage the adoption of walking and cycling, and foster wider benefits in relation to human health and social capital^8–10^.

Posted speed limits are a key determinant in ensuring drivers maintain safe speeds. But it is well acknowledged that in many areas, interventions are required to ensure compliance. Engineering features and physical interventions play an important role. Many studies have shown how traffic calming interventions significantly reduce vehicle speeds and mitigate speeding behaviours^11–14^. Commonly used speed calming features, such as humps^6,15–18^, bumps^19–21^, cushions^19^, and lane narrowing^22,23^, have proven effective in reducing vehicle speeds and alleviating speeding. The implementation of speed cameras has the potential to further diminish the likelihood of speeding on arterials and highways^24^.

However, the effectiveness of traffic calming measures is highly sensitive to urban contexts, and may influence other speeding behaviours. Interventions such as raised crossings and speed humps are found to be most effective on urban and residential streets, but have limited impact beyond the local site^13,17,23^. Vertical inflections, such as humps, reduce average speeds but may increase acceleration noise due to repeated braking and acceleration, whereas horizontal measures such as curb extension tend to generate more consistent speed profiles^22,25^. When traffic calming measures are implemented in isolation or without appropriate spacing, they may act as “punctual” measures and induce rebound effects, with drivers accelerating once past the intervention location^19,23,26,27^.

The role of urban context is therefore important in considering influences on speeding behaviour. Driver speed choice is strongly influenced by spatial perceptions of safe speeds rather than posted limits alone. Compliance may depend on the perceived credibility of the speed limit within a given urban context. Features of the visually perceived environment, such as road characteristics and conditions^1,28–30^, affect these perceptions and subsequent speed selection behaviour. Longer road segments tend to exacerbate speeding^31,32^. Open spaces are associated with increased speeding^33^, while narrower lanes and shorter block lengths exhibit the opposite trend^34^. But the evidence around the role of the posted speed limit in mitigating speeding is mixed. Some studies indicate that roads with lower speed limits^24,34,35^ and congested conditions^36^ are more associated with speeding. While others find that it is arterial roads show greater association^6,31,34^. These findings are across different social contexts, but reveal that the influence of road class and speed limit are not clear.

This lack of clarity may mask an issue of analytical focus. We know from urban and behavioural science that spatial behaviour does not in fact pertain solely to local, visually perceived conditions, but influenced by factors relating to perceived and remembered urban form^27,28,37–40^. A focus on link-level engineering characteristics may therefore neglect the role of global urban form that determine local behaviour. Studies of shared space interventions, for example, show that even the most significant local interventions struggle to counteract the urban context in which they are based^41^. While accounting for other factors, propensity for speeding may in fact emerge where local and global descriptions of the city challenge the credibility of speed limit restrictions. To fully test this hypothesis, we assess the extent to which local and global features of urban space shape speeding behaviour of goods vehicles across 13 British cities, using data from over 3.2 million vehicle journeys.

## Results

**Fig. 1:**
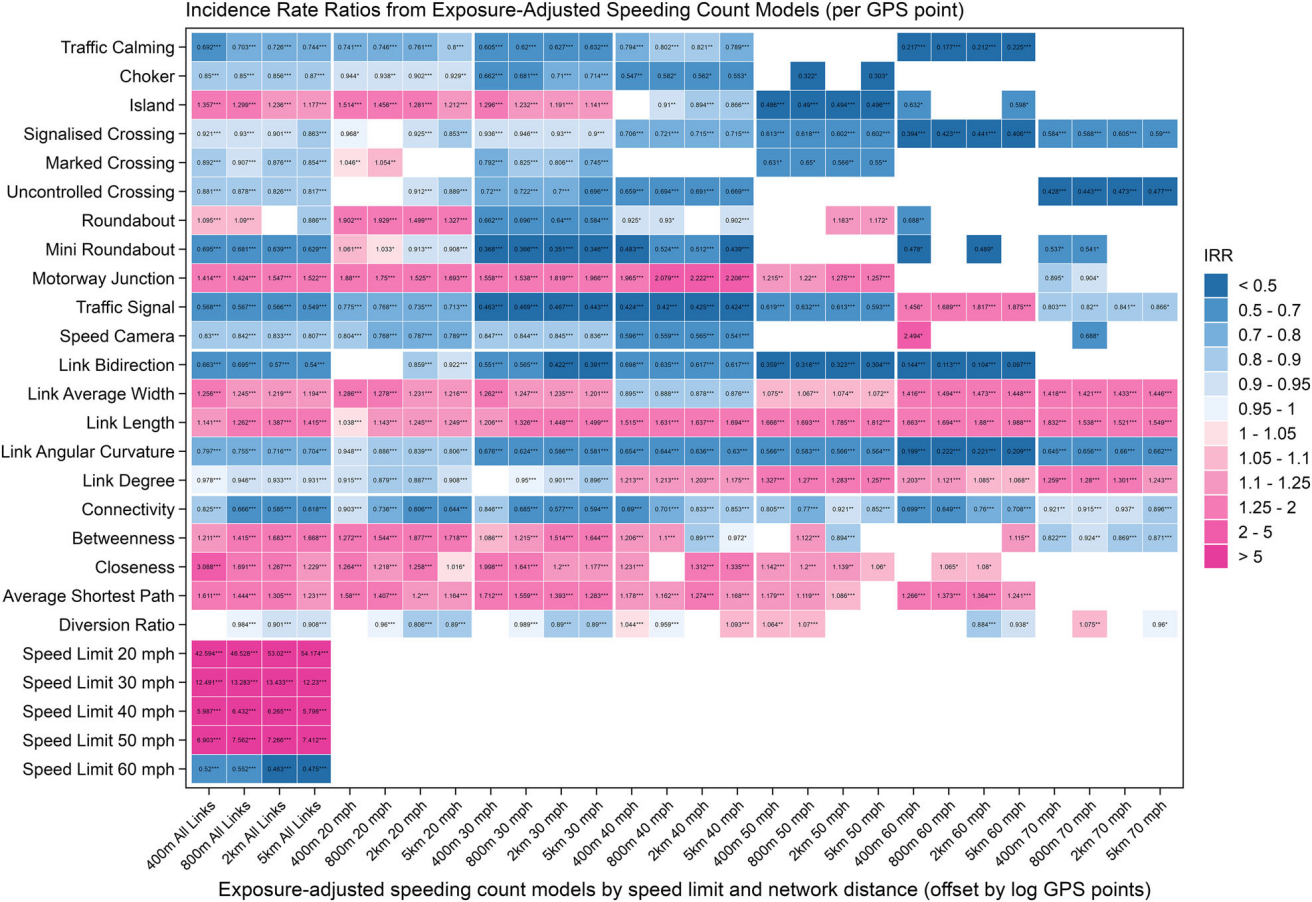
Colour grids show the Incidence Rate Ratio (IRR) for independent variables across negative binomial models (offset by log GPS points) for road links with varying speed limits and network distances. Scores above 1 indicate positive association with speeding incidence per GPS point, scores lower than 1 indicate negative associations with speeding. Network-based independent variables including connectivity, betweenness, closeness, average shortest path, and diversion ratio are adjusted for distance thresholds at 400, 800, 2k, and 5k metres. Speed limits are reported in miles per hour (mph); 20, 30, 40, 50, 60, and 70 mph correspond approximately to 32, 48, 64, 80, 97, and 113 km/h, respectively.

The negative binomial models assessing the affect of different facets of the road network on speeding behaviour are shown in Fig. 1, indicating exposure-adjusted speeding rate per GPS point, incorporating road network form (including road attributes and network metrics) and engineering features. A fuller range of models developed for each speed limit zone can be found in Table S3 to Table S9. A counterpart model is developed to measure effects on speeding incident counts, and thus not controlling for GPS points (see Figure S1 and Table S10 to Table S16). The rates model therefore highlights relative effects between road classes, the events model highlights the prevalence of speeding on each road class. All spatial results of road link-level speeding rates and count of speeding events across British cities can be found in Figure S5 to Figure S8. All continuous numerical variables are scaled to generate standardised coefficients, facilitating comparisons with binary variables within the model. Incidence rate ratio (IRR) serves as a metric for evaluating the strength of comparisons between models. Speeding rate refers to exposure-normalised speeding events per GPS point, whereas speeding events refers to the observed counts of speeding on a road link. Below we summarise the outcomes of these findings by feature group. In all cases, given the broad consistency of findings across distance thresholds, we focus on the 2km radii models for brevity, noting differences across network scales only where relevant.

### Engineering interventions have varying impact on speeding

The first intervention we test is the imposition of speed limits. Where we include speed limits as a fixed effect the model indicates that (using the 2 km network radii as the base case), after accounting for network effects and engineering features, road links with a lower speed limit of 20 mph (*β* = 3.971, CI = [3.923, 4.018], p < 0.001, IRR = 53.020 per point; *β* = 3.098, CI = [3.031, 3.165], p < 0.001, IRR = 22.155 per link) tend to exhibit higher incidence rate ratio of speeding behaviours both for speeding rate per GPS point and speeding count per link compared to the reference speed limit group of 70 mph. Furthermore, road links with posted speed limits of 30 mph (*β* = 2.598, CI = [2.551, 2.644], p < 0.001, IRR = 13.433 per point; *β* = 2.173, CI = [2.108, 2.239], p < 0.001, IRR = 8.788 per link), 40 mph (*β* = 1.835, CI = [1.787, 1.882], p < 0.001, IRR = 6.265 per point; *β* = 1.896, CI = [1.829, 1.964], p < 0.001, IRR = 6.662), and 50 mph (*β* = 1.983, CI = [1.927, 2.039], p < 0.001, IRR = 7.266 per point; *β* = 2.194, CI = [2.115, 2.274], p < 0.001, IRR = 8.974 per link) also experience a higher incidence of speeding compared to those with a speed limit of 70 mph. Only roads with a speed limit of 60 mph (*β* = -0.770, CI = [-0.828, -0.712], p < 0.001, IRR = 0.463 per point; *β* = -1.171, CI = [-1.249, -1.094], p < 0.001, IRR = 0.310 per link) are predicted to have lower incidence rate of speeding behaviours than those with a 70 mph speed limit, both for rate per GPS point and count per link. These fixed effects suggest that road links with lower posted speed limits, such as 20, 30, and 40 mph, may experience a higher prevalence of speeding behaviours, agreeing with findings elsewhere^35^.

The effect of road engineering interventions are measured across different speed limited roads. These interventions are included where located on the focal road link or within a 50-metre network distance from either the start or end nodes. We see that traffic calming measures (including humps, bumps, cushions, and tables) are negatively associated with both speeding rates and incidents (*β* = -0.321, CI = [-0.333, -0.309], p < 0.001, IRR = 0.726 per point; *β* = -0.408, CI = [-0.424, -0.392], p < 0.001, IRR = 0.665 per link), effectively deterring speeding behaviours than other engineering measures on lower speed limits of 20 and 30 mph. Chokers demonstrate a mitigating effect on speeding behaviours (*β* = -0.155, CI = [-0.197, -0.114], p < 0.001, IRR = 0.856 per point; *β* = -0.190, CI = [-0.247, -0.133], p < 0.001, IRR = 0.827 per link)^25^. In contrast, islands are anticipated to predict higher link-level speeding rates and events, with significant positive effects (*β* = 0.212, CI = [0.196, 0.228], p < 0.001, IRR = 1.236 per point; *β* = 0.540, CI = [0.519, 0.562], p < 0.001, IRR = 1.717). When assessing speeding behaviours across speed limit groups, the mitigating influences of traffic calming measures remain pronounced on 20, 30, and 40 mph roads, whereas chokers are effective on 30 and 40 mph roads. Even though islands often predict elevated speeding on lower speed limits of 20 and 30 mph roads, they can act as a deterrent on roads with higher speed limits.

Crossings are consistently negatively associated with speeding rates but positively correlated with road link-level speeding events, including signalised (*β* = -0.104, CI = [-0.120, -0.089], p < 0.001, IRR = 0.901 per point; *β* = 0.246, CI = [0.226, 0.266], p < 0.001, IRR = 1.279 per link), marked (*β* = -0.132, CI = [-0.157, -0.108], p < 0.001, IRR = 0.876 per point; *β* = 0.142, CI = [0.109, 0.175], p < 0.001, IRR = 1.152 per link), and uncontrolled crossings (*β* = -0.191, CI = [-0.208, -0.173], p < 0.001, IRR = 0.826 per point; *β* = 0.217, CI = [0.193, 0.242], p < 0.001, IRR = 1.243 per link). This indicates decreases in speeding rates but increases in speeding incidents around such crossings. The mitigating effects of crossings on speeding rates tend to increase from lower to higher speed limits. The phenomenon pertaining to speeding events per link is especially evident on 20 mph roads, whereas on roads with higher speed limits, this relationship appears to diminish or even reverse.

Junction design has a mixed impact on speeding behaviour. Among the three types of junctions assessed, mini roundabouts (*β* = -0.447, CI = [-0.472, -0.423], p < 0.001, IRR = 0.639 per point; *β* = -0.252, CI = [-0.285, -0.219], p < 0.001, IRR = 0.777 per link) have negative impacts on speeding behaviours, including rate per point and count per link. These effects are pronounced on higher speed limit roads, except for 20 mph. Even though roundabouts predict heightened speeding rates on 20 mph roads, they are expected to reduce speeding propensity in higher speed limits. However, roundabouts (*β* = 0.366, CI = [0.335, 0.397], p < 0.001, IRR = 1.442 per link) are associated with elevated speeding events per link. Motorway junctions (*β* = 0.437, CI = [0.381, 0.492], p < 0.001, IRR = 1.547 per point; *β* = 0.566, CI = [0.486, 0.647], p < 0.001, IRR = 1.762 per link) are associated with a high incidence rate ratio of speeding, both per point and per link. This is particularly true for speed limits not exceeding 50 mph, indicating that proximity to motorway junctions is generally linked to increased speeding behaviours and suggesting a spillover effect of vehicles exiting motorways onto lower speed surrounding roads.

Traffic signals (*β* = -0.569, CI = [-0.584, -0.554], p < 0.001, IRR = 0.566 per point; *β* = -0.191, CI = [-0.210, -0.171], p < 0.001, IRR = 0.826 per link) are negatively correlated with both speeding rates and incidents, demonstrating mitigating effects on speeding behaviours. They usually exhibit mitigating impacts on speeding propensity per point across varying speed limits, except for 60 mph. However, traffic signals are associated with an increase in speeding events per link on 20, and 60 mph roads, but with significantly fewer speeding incidents on 30, 40, and 50 mph roads. Speed cameras are negatively correlated with speeding rate per point (*β* = -0.183, CI = [-0.237, -0.129], p < 0.001, IRR = 0.833 per point). However, the presence of speed cameras (*β* = 0.371, CI = [0.294, 0.448], p < 0.001, IRR = 1.449 per link) is positively correlated with speeding incidents, particularly on 30 and 60 mph roads. As such, the results suggest that while cameras can reduce speeding propensity, they are located on roads with higher overall incidence of speeding. Any effect on speeding by cameras is likely to be hyperlocal, and not consistently extend to adjacent road links, particularly in British cities where the implementation of camera-based speed enforcement is not widespread.

### Road design can help control speeding

The effect of the basic design attributes of road links, such as direction, width, length, angular shape, and degree, are considered next. These characteristics are shown to have stronger impacts than engineering, producing positive and negative impacts on speeding outcomes.

Dual-directional roads are linked to lower speeding rates and fewer speeding incidents (*β* = -0.562, CI = [-0.575, -0.550], p < 0.001, IRR = 0.570 per point; *β* = -1.114, CI = [-1.131, -1.097], p < 0.001, IRR = 0.328 per link). These roads exhibit a mitigating influence across all speed limits, indicating that single-direction roads substantially contribute to an increase in speeding behaviour.

Wider roads are associated with a higher incidence of speeding pertaining to rate per point and count per link (*β* = 0.198, CI = [0.193, 0.202], p < 0.001, IRR = 1.219 per point; *β* = 0.757, CI = [0.750, 0.765], p < 0.001, IRR = 2.132 per link). The strongest predictive effect is observed on roads with a 60 mph speed limit, followed by notable impacts on 30, 20, 70, and 50 mph roads. Interestingly, width shows no significant correlation with speeding behaviours on 40 mph roads, and in fact may slightly mitigate speeding at this speed. Similarly, longer road links demonstrate a strong positive correlation with both speeding rates and speeding incidents (*β* = 0.327, CI = [0.322, 0.332], p < 0.001, IRR = 1.387 per point; *β* = 1.397, CI = [1.388, 1.406], p < 0.001, IRR = 4.043 per link), indicating that these links are associated with a significantly higher propensity and occurrence of speeding. This pattern persists across most speed limit categories, with link length exhibiting the strongest positive association with speeding behaviours, especially higher speed roads.

Road links with greater angular variation are negatively associated with speeding behaviours (*β* = -0.334, CI = [-0.339, -0.330], p < 0.001, IRR = 0.716 per point; *β* = -0.508, CI = [-0.514, -0.502], p < 0.001, IRR = 0.602 per link), suggesting that some angular curvature in road design may help mitigate speeding. This effect is present across all speed classes, although strongest on 50 and 60 mph roads, with a diminished impact on 20 mph roads.

Link degree connectivity is overall negatively associated with speeding rates and incidents (*β* = -0.070, CI = [-0.073, -0.066], p < 0.001, IRR = 0.933 per point; *β* = -0.112, CI = [-0.117, -0.107], p < 0.001, IRR = 0.894 per link). This effect is particularly evident on roads with speed limits of 20 and 30 mph. However, on roads with higher speed limits, individual link connectivity reverse such trend, indicating elevated speeding behaviours.

### Road network context may mitigate effects of engineered interventions

Network-based metrics are calculated and assessed for each road link across network distance bands, with design and engineering measures maintained within the model as controls. The metrics assessed include network connectivity, betweenness centrality, closeness centrality, average shortest path length, and diversion ratio.

Road network connectivity (*β* = -0.536, CI = [-0.541, -0.530], p < 0.001, IRR = 0.585 per point; *β* = -0.817, CI = [-0.824, -0.810], p < 0.001, IRR = 0.442 per link) exhibits a robust negative correlation with both speeding rates and speeding incidents, highlighting its substantial role in curbing speeding behaviour^6^. This finding indicates that well-connected and denser road networks significantly contribute to better compliance with speed limits. The mitigating effect of connectivity on speeding is consistently observed across various speed limits, with the impact being particularly pronounced on roads with lower speed limits, such as 20 and 30 mph.

Both betweenness centrality (*β* = 0.521, CI = [0.515, 0.527], p < 0.001, IRR = 1.683 per point; *β* = 1.074, CI = [1.065, 1.083], p < 0.001, IRR = 2.927 per link) and closeness centrality (*β* = 0.237, CI = [0.211, 0.263], p < 0.001, IRR = 1.267 per point; *β* = 0.395, CI = [0.358, 0.432], p < 0.001, IRR = 1.485 per link) exhibit an overall positive relationship with speeding behaviours both for rates and incidents, with the strongest effect at low speed roads of 20 and 30 mph, but a mitigating effect in 70 mph zones. Streets with high centrality generally provide accessible connections to other parts of the network, and as such, may be perceived by drivers to be higher speed roads. Where a contrast emerges between centrality and the posted speed limit—at 20 and 30 mph zones, and not 70 mph zones—a higher rate of speeding behaviour is observed. This indicates that roads of greater structural prominence, relative to their speed limit, are more vulnerable to speeding behaviours.

Average shortest path (*β* = 0.266, CI = [0.262, 0.270], p < 0.001, IRR = 1.305 per point; *β* = 0.337, CI = [0.331, 0.342], p < 0.001, IRR = 1.401 per link) is positively associated with an increase in speeding behaviours. This indicates that road segments characterised by higher average shortest path values—reflecting lesser proximity to other roads—tend to experience an elevated occurrence of speed limit violations. Notably, such positive relationships is particularly pronounced on lower speed roads. These results underscore the significant influence of less dense areas and inefficiency parts of the road networks on speeding.

In addition, the diversion ratio typically shows negative relationships with speeding behaviours, and its mitigating effects are most evident on roads with lower speed limits of 20 and 30 mph, as well as those with higher limits of 60 and 70 mph on a global network scale. Conversely, on roads with speed limits of 40 and 50 mph, diversion ratio is often associated with a higher propensity and frequency of speeding events across most network distances.

### Causal associations with road network and engineering measures

**Fig. 2:**
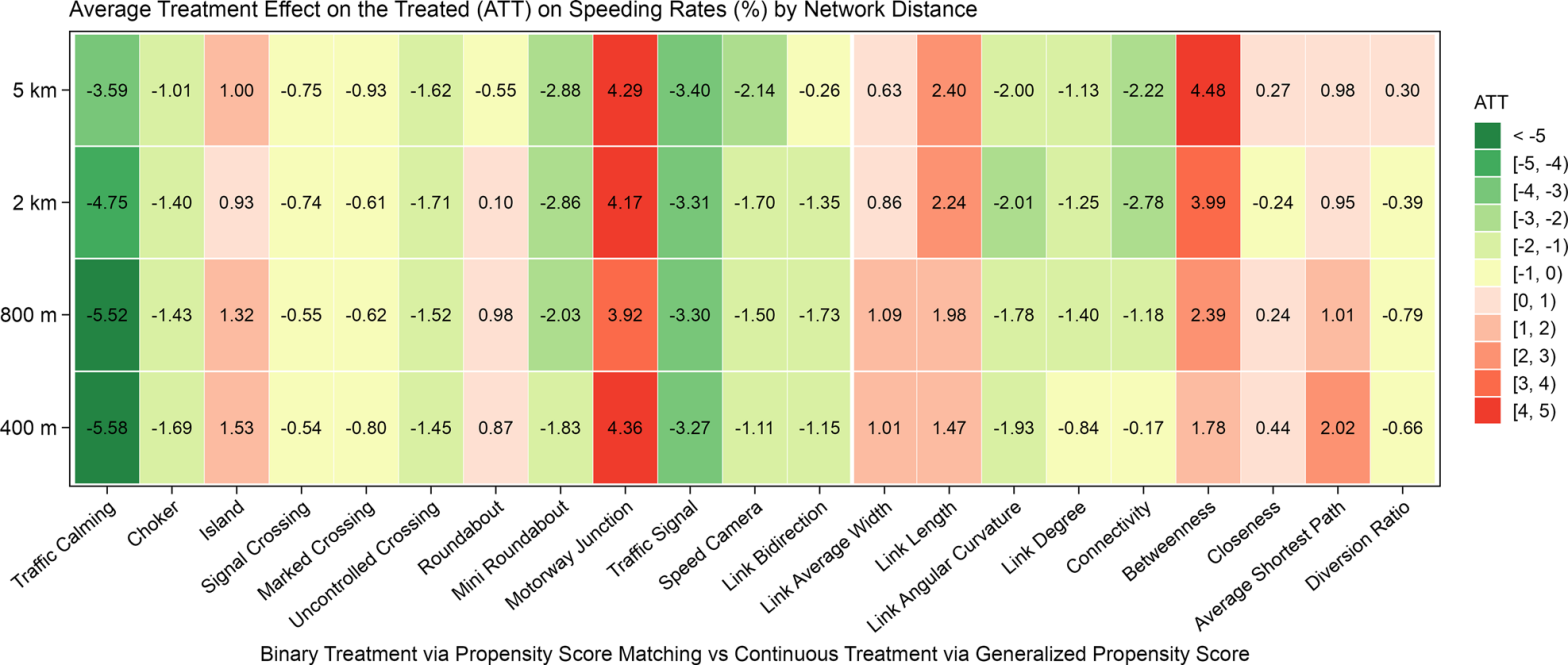
Colour grids show the Average Treatment Effect on the Treated (ATT) via differences of speeding rates (%) for treatment factors across four spatial scales, where positive values indicate increases in speeding percentage and negative values relate to reductions in speeding percentage; interpretation of values differ between binary (difference between mean of treatment group and control group) and continuous variables (difference between treatment average and one standard deviation increment from exposure-response function). Speed limits are reported in miles per hour (mph); 20, 30, 40, 50, 60, and 70 mph correspond approximately to 32, 48, 64, 80, 97, and 113 km/h, respectively.

The cross-sectional results above, derived from the negative binomial model, might introduce confounding bias in the associations between the independent variables and speeding behaviours. As such, we extend our analysis by incorporating causal models, including propensity score matching (PSM) and generalised propensity score (GPS) methods, to estimate the average treatment effect on the treated (ATT) individual variable while adjusting for a reasonable balance of other confounding factors. Robust causal results on all road links across network distance bands are interpreted as adjusted effects consistent with plausible causal mechanisms. The causal differences for speeding rates are shown in Fig. 2, the equivalent differences for speeding events are shown in Figure S2. Full elaboration of the modelling, incorporating a broader range of parameters, are shown in Table S17 to Table S20. Diagnostics for all causal models, including the robustness-to-unmeasured-confounding of E-values, are provided in Figure S3 and Figure S4 and Table S21 and Table S22.

The causal robustness analysis supports the trends identified in cross-sectional statistical modelling. Once more we observe that engineering interventions are associated with speeding rates and speeding events, while road design attributes, as well as the network metrics, exhibit significant influences on speeding behaviours.

The ATT measures via differences of outcomes enable direct extraction of the impact of each intervention on speeding behaviours both for speeding rates (see Fig. 2) and speeding events per link (see Figure S2). For instance, the impact of traffic calming interventions leads to a reduction of 4.75% in speeding rate and 9.09 speeding events on links in the 2 km model. In terms of both rates and events, this effect is comparable to achieving an increase of just one standard deviation from a baseline average when examining total connectivity within a 2 km network distance. Moreover, it is plausible that at an 800-metre network scale, the enhancement of network connectivity may exceed the hyper-local effects of traffic calming interventions in mitigating speeding events throughout the entire street network.

Engineering measures such as traffic signal, mini roundabout, and choker counteract speeding behaviours in both models. Crossings demonstrate a reduction in speeding rates, but are related with speeding occurrences. A similar trend is observed with speed cameras - likely an indication of their higher prevalence on high-flow roads. In contrast, traffic islands and motorway junctions are associated with elevated speeding behaviours. Wider and longer road segments again are related to speeding, while angular curving roads demonstrate a relationship with reduced speeding. Betweenness centrality shows a positive relationship with speeding behaviours, particularly when measured across larger network scales, while average shortest path exhibits a positive effect on local network scales.

## Discussion

It is easy to consider vehicular speeding a reckless act of non-compliance with the law, requiring strict oversight in the form of physical infrastructure and restrictions. But speeding in cities occurs within a wider context. Clearly the role of social context in shaping speeding behaviour is fundamental and fairly well understood - norms in driving behaviour are shaped and reinforced by the law, training, and public messaging^42,43^. But within the hyper-local setting, of where a driver perceives local conditions and selects a speed along a road, an important context is that of the road and its placement within the wider city^29,30^. These features are in part determined by road engineering, but also by physical geography, historic urban development, and transportation planning. Too often our consideration of the local context ends at the assessment of engineering features.

Through this study of speeding by goods vehicles in British cities, we observe that roads with lower speed limits often experience higher rates of speeding behaviour. Lower speed limits are assigned to ensure road safety, yet in specific contexts, they may possess similar geometric and contextual characteristics to higher speed counterparts. We suggest this perceived discrepancy challenges the *credibility* of posted speed limits in these contexts. To some extent these discrepancies are effectively countered by speed reduction infrastructure, but at least in the case of goods vehicles, in certain locations their effectiveness is mitigated by network positioning and road geometry. As a result, the effectiveness of engineered interventions are mixed - on some roads we observe a beneficial impact on speeding, on others, particularly on lower speed roads, their effectiveness is reduced.

The findings suggest a need for greater focus on road geometry and its location within the wider network in mitigating speeding. At the most local scale, we see that winding roads naturally discourage higher vehicle speeds, while longer and wider roads are strongly associated with a higher incidence of speeding. This effect persists across speed limit zones. These are characteristics of the local environment determined by geography and history of an environment, but are features can be identified, and mitigated, at a local scale.

Taking a broader view of the role of the street within the wider road network, we see that average shortest path indicator is associated with increases in speeding events, particularly at a local network scale. This finding suggests that speeding propensity increases when longer routes are imposed by the road network structure. These effects are particularly pronounced on roads with lower speed limits of 20 mph and 30 mph. This suggests that local natural barriers (e.g. parks, rivers) and road closures, including those associated with enhancing local road safety, may inadvertently increase speeding propensity on nearby roads. These findings also imply that a well-connected street network can be expected to see less speeding behaviour than an area with heterogeneous street design. The increased variability in street design may act to promote speeding behaviour on streets where opportunity allows.

Measures of centrality provide an indication of how important a road may be within the wider street network. We see that links with higher betweenness and closeness centrality tend to experience a greater frequency of speeding incidents, especially for closeness at a local scale, while betweenness at a more broader global scale. This trend is strongest on lower speed limit roads but diminishes at 40, 50, and 60 mph speed limits, and is absent on 70 mph roads. This trend further evidences the conflicts between speed limit setting and network characteristics. Roads with a large centrality may, by virtue of their broader structural features, promote a perception in drivers of a higher speed limit than that defined by traffic regulations. This is not an issue on high-speed roads, which are designed to be central to the road network, but is an issue where centrality is higher in lower speed areas. Such roads are central as a product of their placement within the wider city (e.g. a main street), perhaps as a facet of historic urban development more than present day function. Nevertheless, this does not suggest that enhancing network closeness correlates with an increase in speeding behaviours; rather, when strengthened at broader spatial scales, it can act as a deterrent to such behaviours.

The findings have several implications for how we consider urban road design and speeding interventions. While engineering interventions undoubtedly have a major role to play, it is evident that longer and wider roads provide affordance to drivers that higher speeds are expected, and by virtue of their design alone, create potentially dangerous conditions. Similarly, we observe speeding in contexts where the betweenness or closeness centrality is high, again potentially challenging the *credibility* of the speed limit posted. These findings suggest there are contexts where road structural and design characteristics may inadvertently promote speeding behaviour. These are factors that may not have been considered in the local engineering of these streets, but by virtue of their natural shape or historic connectivity, are perceived by drivers to enable a certain safe driving speed. Such contexts - where local requirements do not match the expectations created by the built environment - require differentiated consideration where targetting speeding behaviour. Tackling speeding in these contexts, as such, requires a shift in perspective from urban planners and traffic engineers.

The complex role that network configuration plays in shaping speeding as implications for current urban planning developments, particular where road closures are considered to increase neighbourhood walkability and air quality. The presence of junctions has a strong mitigating effect on speeding, and increases in the length of paths required to traverse an area appear to lead to more speeding incidents. As such, the closure of junctions may conversely promote speeding opportunities in some areas. Further work is needed here to provide a stronger cause and effect relationship, and any changes to the road network be considered in the round with the wider benefits of such schemes.

Further exploration is needed around the nature of street design in influencing the prevalence of speeding in different contexts. It is clear that physical measures imposed by road traffic calming schemes have some effect, particularly on 20 mph roads. However, there are implications of these findings are that carefully planned adjustments to street network structure may have greater cost benefit as an intervention on speeding. This is retained for further work.

There are broader considerations arising from this study for urban analytical study. The rise of large-scale data on human activity in cities has enabled a more granular focus on the spatial and temporal determinants of behaviour. But a study such as this demonstrates that the contextual factors influencing behaviour may in fact occur at multiple scales. While streets are where we experience the city, we do so in the knowledge of a wider spatial context. This global context, and its multifaceted social and engineering features, shaped by decades or centuries of urban development, influences perception of local places.

Network measures provide a way of measuring global structure of urban road networks. Often these measures are used to explain or predict macroscopic urban phenomena, such as activity and flow. But there appear to be opportunities to consider how these global structures influence local outcomes too. There are suggestions here that there are contexts where the design or designation of a local area conflicts with what implied by its global situation. Where a discrepancy between local and global inference occurs, where the expectation of a space contradicts the reality, we may observe unexpected or unanticipated consequences. In this case we have seen this in speeding behaviour of goods vehicles, but opportunities for considering this phenomena potentially extend more widely.

The findings from this study provide an indication of potential new considerations for transport and urban planners in mitigating speeding, but there are several major caveats to consider in addressing these findings. First, the data used in this study relates to goods vehicles only. This means these are the behaviours of professional drivers - potentially strengthening causality, given these drivers are less likely to be motivated to break speed limits, and may be penalised by employers for doing so - but will thus use particular routes and engage in practices that may not be applicable to wider vehicular behaviour. The effect of goods vehicle driving practices on speeding are difficult to determine, but can be expected to differ somewhat from other vehicles. Goods vehicles also exhibit certain physical characteristics that limit their capability to speed up or slow down in the same ways as all other vehicles.

The nature of this study - being a one month cross-sectional investigation - means we lack true causality around the role of each feature in influencing behaviour. There are several potential sources of unobserved confounders (e.g. police action, roadworks, land use variation, driver variation) that are not captured in the data. We lack coverage of data to enable a deeper exploration of temporal variation and the role of congestion in limiting speeding. The data used in the study relates to a single GPS points collected at 10-second intervals on the road link, rather than as a continuous episode at the trip-level, which may introduce potential bias. The modelling employed here does not fully address the potential for spillover effects (and spatial autocorrelation) arising from different interventions.

The findings presented in this study, as well as the limitations, point to the need for further work to uncover the relevance of context in influencing driver behaviour. It is a prompt to encourage transport planners to consider the wider context in understanding speeding problems. To get there, there remains a need to strengthen the evidence base, by assessing speeding in relation to a roads network context or geometry. This evidence base can be developed by broadening the types of data used to explore speeding, or through smaller scale site monitoring. While engineering interventions will always play a role in alleviating problems, adjustments to network configurations offer an alternative, and if accompanied as part of wider street design changes, be cost effective interventions.

## Methods

### GPS trajectory data

This study utilises a large dataset of vehicle GPS trajectories, supplied by Compass IoT, collected over the course of one month in October 2023 across the United Kingdom. The dataset includes more than 3.2 million trips recorded by over 930,000 vehicles, with each trip entry comprising detailed vectors, such as snapped coordinates, speed, and timestamps. The source is open data, available for download on the Healthy and Sustainable Places Data Service (HASP) repository at the following: 10.82147/010.

For each trip GPS coordinates are collected on a 10 second frequency, with each GPS point associated with a corresponding timestamp and speed, collected from the vehicle. For the purposes of this study, we use only data on trips generated by light good and heavy goods (LGV and HGV) vehicles. Although we lack trip purpose and driver attribute data, we may assume these trips relate to professional driving contexts (e.g. courier, delivery) and naturalistic speed data, and as such, instances of speeding behaviour may better exhibit differences in perception of speed limits and urban contexts, rather than other social determinants. The data, however, only encompass only those integrated into the Compass connected car system, and may not be fully representative of all vehicle journeys.

### Focus cities

The study covers the 13 largest city regions of Great Britain, those being Greater London (London), Greater Manchester (Manchester), Oxford, Cambridge, Cardiff, West of England, the West Midlands Combined Authority (West Midlands), the Liverpool City Region (Liverpool), the West Yorkshire Combined Authority (West Yorkshire), South Yorkshire, Tyne and Wear (Newcastle), Edinburgh, and Glasgow. Table 1 shows how link-level speeding rates varies across cities by speed limit group, the measure of speeding points and total GPS points is defined in measurement of speeding behaviour.

**Table 1:** Speeding rates (%) by speed limit group

	Total	20 Mph	30 Mph	40 Mph	50 Mph	60 Mph	70 Mph
London	7.83	13.26	4.9	5.48	9.87	5.59	3.08
Manchester	6.9	20.77	9.59	6.82	7.84	4.75	3.19
Oxford	7.4	10.79	4.02	5.78	9.98	0.0	0.6
Cambridge	8.16	15.3	5.61	4.14		0.0	2.41
Cardiff	6.95	14.0	4.8	6.94	18.96	0.07	5.18
West of England	10.2	25.66	12.97	10.18	8.99	0.56	5.21
West Midlands	7.14	24.2	9.87	6.96	12.85	0.95	0.73
Liverpool	9.2	23.59	12.27	7.03	8.39	4.61	4.38
West Yorkshire	6.72	23.63	10.02	6.99	11.49	0.52	3.17
South Yorkshire	5.46	29.01	12.36	7.41	8.12	0.48	2.68
Newcastle	8.51	23.21	11.89	5.79	10.46	0.3	1.84
Edinburgh	10.27	21.6	12.8	9.95	21.24	0.93	1.71
Glasgow	16.06	15.83	9.92	7.96	34.43	15.71	3.38

20 mph includes road links with posted speed limits at 5, 10, 15, and 20 mph. Speed limits are reported in miles per hour (mph); 20, 30, 40, 50, 60, and 70 mph correspond approximately to 32, 48, 64, 80, 97, and 113 km/h, respectively.

### Trajectory data processing

Before assessing speeding behaviour by calculating speeding events, several preprocessing steps were applied to the raw Compass GPS data. Due to a small offset between the GPS points and the road lines in the spatial data—likely caused by the spatial simplification of road geometries or biases inherent to location-based services—we first removed any points where the closest distance to the road links exceeded 10 metres. Next, we excluded all GPS points that were recorded with a speed of 0 mph from the dataset. Additionally, GPS trips containing only a single GPS point were discarded to ensure the availability of meaningful trip data.

Following these preprocessing steps, we applied a Hidden Markov Model (HMM)-based map-matching algorithm following the framework of^44^, implemented using the GoTrackIt Python package^45^. Emission probabilities were determined by the spatial proximity between GPS points and candidate road segments, while transition probabilities accounted for network connectivity, path distance consistency, and GPS heading continuity. Key parameters were adjusted to improve matching accuracy, including the GPS search buffer, the number of candidate road links retained, and the use of GPS heading information, enabling accurate alignment of GPS trajectories with the underlying road network.

### Highway road link data

Road links derived from the Ordnance Survey (OS) Highways dataset served as the spatial units for matching GPS trajectory points. The dataset provides a detailed and topologically consistent representation of the road network, comprising well-integrated links and nodes. Each road link represents an individual edge in the network and is uniquely identified by a Topographic Object Identifier (TOID). Links are delineated not only by network nodes such as junctions and intersections, but also by changes in geometry or road attributes, including road type and posted speed limit. Consequently, a continuous roadway may be subdivided into multiple links where its physical form or functional characteristics change—for example, where a straight segment transitions into a bend or where a minor path intersects and segments the carriageway. Each link therefore constitutes a discrete road segment with relatively homogeneous design and regulatory attributes, making it an appropriate unit for analysing speeding behaviour and link-level traffic characteristics. Following the HMM-based map-matching, most road links had GPS points assigned to them, enabling link-level analysis of speeding behaviour.

Each road link is characterised by attributes such as speed limits and road hierarchy. The speed limits are categorised into values of 5, 10, 15, 20, 30, 40, 50, 60, and 70 mph. The road hierarchy is classified into Motorway, primary A Road, A Road, B Road, Local Road, Minor Road, Secondary Access Road, Local Access Road, Restricted Secondary Access Road, and Restricted Local Access Road. Based on the data distribution, we reorganised the speed limits into groups of 20 mph (including 5, 10, 15, and 20 mph), 30, 40, 50, 60, and 70 mph. We regrouped the lower speed limits of 5, 10, and 15 mph into the category of 20 mph due to the small proportion of road links with these lower speed limits in the dataset. This adjustment ensures a more robust statistical analysis by consolidating categories with limited data points. All speed limits are reported in miles per hour (mph), consistent with traffic regulations in British cities. For ease of interpretation, 20, 30, 40, 50, 60, and 70 mph correspond approximately to 32, 48, 64, 80, 97, and 113 km/h, respectively. Similarly, the road hierarchies were grouped as Motorway, A Road (including primary A Road and A Road), B Road, Local Road, Minor Road, and Others (including various access roads such as Secondary Access, Local Access, and Restricted Access Roads). Despite the inherent data sparsity and minimal traffic loads characteristic of these lower-tier segments, all classifications were retained to preserve topological network integrity. This ensures the model accurately captures the critical spatial transition from high-velocity arterial transit to the low-volume local movement found at residential destinations.

The summary statistics on speeding behaviours include all road links with GPS points captured following the data preprocessing and filtering stages. The filtering stages are shown in Table 2. In total, there are 1,007,729 road links in the highways dataset. These road links are further differentiated by their road form attributes, including dual carriageway, single carriageway, track, traffic island link, roundabout, shared use carriageway, slip road, enclosed traffic area, guided busway, and lay-by. For our statistical analysis, we focused exclusively on dual and single carriageways, excluding other forms. This filtering resulted in 892,934 road links of interest. Additionally, 21,410 road links were missing the average width attribute, leading to a final total of 871,524 observations for our analysis. For number of road links by speed limit groups of 20, 30, 40, 50, 60, and 70 mph across British cities, there are 332,638, 479,095, 34,349, 7,597, 12,222, and 5,623 road links, respectively. It should be noted that the full OS Highways dataset is used to calculate network metrics in our analysis. Table S1 provides more detailed information on the GPS points, speeding points, and road links by speed limit across 13 British cities, and Figs. S5–S8 show the spatial distribution of speeding rates and speeding events in each city.

**Table 2:** Summary statistics of Compass GPS data

	Matched points	Unique trips	Points filtered	Percentage of points filtered	Points in modelling	Matched links with points assigned	Links filtered	Links in modelling
London	129,196,243	1,046,985	129,071,543	99.90%	118,321,296	345,287	296,056	253,409
Manchester	38,527,118	337,090	38,422,668	99.73%	35,190,390	175,692	130,501	115,542
Oxford	3,992,619	56,557	3,990,978	99.96%	3,714,943	8,908	8,376	7,383
Cambridge	1,112,628	42,236	1,107,969	99.58%	1,005,794	7,775	6,091	5,267
Cardiff	3,961,236	50,195	3,950,833	99.74%	3,468,509	18,885	14,238	12,003
West of England	18,926,098	164,596	18,897,915	99.85%	17,352,934	60,402	48,278	41,475
West Midlands	41,142,376	416,188	41,056,915	99.79%	37,461,592	158,774	129,478	111,598
Liverpool	23,572,348	261,785	23,515,798	99.76%	21,430,620	101,616	79,441	69,431
West Yorkshire	41,877,711	311,586	41,789,262	99.79%	38,685,062	157,483	119,035	105,597
South Yorkshire	25,509,616	187,580	25,463,831	99.82%	23,629,615	84,251	63,522	54,671
Newcastle	8,992,083	87,132	8,941,357	99.44%	7,961,628	77,449	54,508	44,512
Edinburgh	9,656,620	97,113	9,641,427	99.84%	8,754,424	27,932	22,578	19,305
Glasgow	15,071,730	205,689	15,054,063	99.88%	13,661,728	41,899	35,627	31,331
All cities	361,538,426	3,264,732	340,365,559	99.78%	330,638,535	1,266,353	1,007,729	871,524

Matched points is the GPS trajectory points assigned to road links after map-matching; Points filtered is points excluding links fewer than 5 assigned; Points in modelling are the GPS points finally matched with Single and Dual carriageways.

### Measurement of speeding behaviour

Prior studies have developed various approaches to define and measure speeding behaviours using naturalistic driving data such as speeding episode^5,36^. Given the inherent attributes of snapped GPS points recorded at 10-second intervals and the spatial unit of road links, we directly measure speeding behaviours by counting the number of speeding events (also referred to as speeding incidents or speeding instances) for each road link. This is calculated from snapped GPS points where the recorded speed exceeds the posted speed limit. The presence of a speeding point on a specific road link is regarded as a speeding event, which also accounts for continuous road traffic conditions. Speeding events are analysed for each city and further categorised by speed limits. The total number of speeding events for a given road link *l* is defined as

1 $${N}_{\mathrm{speeding}}(l)={\sum }_{i=1}^{{N}_{l}}I({v}_{i} > {S}_{l})$$

where, *N*_speeding_(*l*) denotes the total number of speeding events observed on road link *l*, calculated by summing over all GPS observations on that link. Here, *N*_*l*_ represents the total number of GPS points recorded on road link *l*, and *v*_*i*_ is the speed associated with the *i*-th GPS point. The term *S*_*l*_ refers to the posted speed limit for road link *l*. The indicator function *I*(*v*_*i*_ > *S*_*l*_) takes a value of 1 when the observed speed at the *i*-th GPS point exceeds the speed limit, and 0 otherwise, thereby counting only those instances where speeding occurs.

This formula quantifies the number of GPS points that exceed the speed limit on each road link, as a proxy for link-level speeding incidents or speeding events, thereby establishing a foundation for our analysis of speeding behaviour across cities, variations in speed limits, and statistical modelling.

### Road network and engineering measures

Road links derived from the OS Highways dataset constituted the road network utilised in this study. For each link, we extracted a set of structural and regulatory attributes, including posted speed limits, direction, average width, and length from OS Highways, along with angular curvature and link degree derived from Spatial Design Network Analysis (sDNA). Angular curvature represents the cumulative angular change along the link geometry, while link degree captures local link connectivity. These link-level attributes characterise the geometric and functional properties of each road link and were used as constant covariates in the analysis.

For road engineering measures, we first collect data on infrastructure elements such as traffic calming measures, crossings, junctions, traffic signals, and speed cameras from OpenStreetMap (OSM). Traffic calming, as identified by OSM tags, encompasses engineering measures and interventions designed to slow or reduce motor vehicle traffic, thereby enhancing the safety of pedestrians and cyclists. Examples of traffic calming features include humps, bumps, cushions, tables, chokers, and islands. Crossings refer to designated locations where pedestrians, cyclists, or equestrians can safely traverse the streets. Junctions denote locations where two or more roads converge or intersect, allowing vehicles to transition from one road to another. These features of the road infrastructure are extracted using OSMnx^46^.

Engineering features derived from OpenStreetMap (OSM) tags were harmonised into analytically consistent subcategories. Traffic calming measures, for example, are grouped into specific types such as humps, bumps, tables, cushions, chokers, and islands (e.g. pedestrian refuge islands). Due to their similarities in vertical deflection and the volunteer-contributed nature of the OSM data, we merge humps, bumps, tables, and cushions under a unified category of “traffic calming”. Chokers are typically horizontal curb extensions designed to restrict vehicle speeds and enhance pedestrian safety. Crossings are classified into signalised (light-controlled), marked (usually with zebra markings), unmarked (lacking markings or signals) and uncontrolled (crosswalks without lights, often with refuge islands). Our analysis focuses on signalised, marked, and uncontrolled crossings. In particular, islands are tagged in OSM within both traffic calming and crossing categories, and we retrieve tags for islands in both contexts. Junctions are categorised as roundabouts, mini-roundabouts, circular junctions, turning circles, and motorway junctions. Due to their similarity, roundabouts and circular junctions are combined under “roundabouts”, and turning circles are excluded. Finally, traffic signals and speed cameras are extracted as stand-alone categories, as they lack further subdivisions in OSM tags.

To assess the availability of road engineering features at the link level, we initially identify the snap vertex of the road engineering features to their nearest road links. Subsequently, we convert our road links, which have snapped engineering measures, into an undirected multigraph utilising NetworkX. For each focal link, we measure the presence of road engineering features within 50 metres. This measurement is calculated for each focal link, taking into account both the start and end nodes to ensure precise access to the road engineering features. The availability of these engineering features is defined as a binary variable for each focal link, rather than as counts or density factors over larger network distances. The presence of engineering factors within 50 metres can be regarded as local effects for the focal link, incorporating acceleration and deceleration associated with the infrastructure, and is reasonably reflect drivers’ perception^18,19^.

In addition, space syntax metrics for each road link are measured using the Spatial Design Network Analysis (sDNA) tool. The key network indicators selected for analysis include total connectivity (sum of number of links ends connected at each junction), betweenness centrality, closeness centrality (i.e., the reciprocal of farness in sDNA), mean geodesic length (i.e. average shortest path), and diversion ratio. In the sDNA framework, a higher connectivity signifies an increased number of road links (i.e., total links) and street intersections (i.e., total junctions) accessible from the focal link within a specified network distance, indicating enhanced network integration and density. Preliminary tests of the model revealed that connectivity exhibits substantial correlation and collinearity with network quantity, number of links, total length, and junctions in sDNA metrics. Betweenness centrality, or choice frequency, quantifies how often a road link is used as part of the shortest path between other links, highlighting its importance in the network. Closeness centrality is a measure of how “close” a link is to all other links within specific network distance. A link with high closeness centrality can quickly access other parts of the street networks. The average shortest path distance calculates the path distance from a given link to all other links in a certain network distance. A lower average shortest path value signifies improved movement efficiency between nearby locations, while a higher value indicates that road links are less clustered within the road network. Lastly, the diversion ratio is the average path distance to crow-flight distance over all links within a specified radius. This metric indicates how much longer the travel routes are compared to the ideal straight-line path within a specified network distance. Other critical metrics in sDNA framework were not included, such as link-level sinuosity (i.e., the line length divided by distance as the crow flies between its endpoints, that is similar to diversion ratio but for a single line only), network distance-level angular distance (i.e., total angular curvature on all links in the radius, which is correlated with total connectivity, and network quantity), and network distance-level mean crow flight (i.e., the mean of the crow flight distance between each origin and all links within the radius, which is correlated with mean geodesic length).

Using sDNA tool in QGIS, we calculate link angular curvature, link degree and network distance-adjusted metrics for each road link by setting parameters that include a vehicle radius metric, length-based weighting, and the enforcement of a one-way restriction. In particular, network distance-adjusted metrics including connectivity, betweenness, closeness, average shortest path, and diversion ratio are assessed within network radii of 400, 800, 2000, and 5000 metres, and adjusted consistently within the same model. We selected 2000 metres as our primary model result in order to assess the influence of network scale on vehicle mobility. The 400 and 800-metre radii correspond to measurements at the local street network scale, while the 5000-metre radii provides a global view of network integration at broader scales.

### Negative binomial models

A negative binomial mixed effects model has been employed to investigate the relationship between speeding events and road link characteristics. This modelling approach facilitates the consideration of both fixed and random effects. In our analysis, speed limits were incorporated as fixed effects, while British cities were designated as random effects. The fixed effect addresses variations in speeding behaviours associated with different speed limits, as road links with varying posted speed limits are expected to exhibit distinct speeding patterns and traffic conditions. The random effect captures the differences among British cities, which may possess underlying factors not explicitly included in the model, such as local transportation policies and regional contexts.

The negative binomial model is well suited for overdispersed count data—where the variance exceeds the mean—at the road-link level. This model allows zeros to arise from the count process rather than from a separately modelled zero-generating mechanism. To account for heterogeneity in both traffic exposure and GPS sampling intensity across road links, we firstly estimated exposure-adjusted negative binomial models by including the logarithm of the total number of GPS observations on each link as an offset. By modelling speeding events relative to observed GPS volume, this specification reduces bias arising from uneven vehicle coverage across the road network and estimates the expected rate of speeding events per GPS point. The offset in negative binomial model adjusts for both traffic exposure and GPS sampling intensity. Accordingly, incidence rate ratios from the exposure-adjusted models reflect relative changes in speeding rates conditional on observed GPS sampling, rather than absolute changes in event counts. The model is specified as follows:

2 $$\log \left(E({Y}_{ij})\right)=\log ({\mathrm{GPS}}_{ij})+{\beta }_{0}+{\beta }_{1}{X}_{1ij}+{\beta }_{2}{X}_{2ij}+{u}_{j}$$

Equivalently, the model can be expressed as a rate model:

3 $$\log \left(\frac{E({Y}_{ij})}{{\mathrm{GPS}}_{ij}}\right)={\beta }_{0}+{\beta }_{1}{X}_{1ij}+{\beta }_{2}{X}_{2ij}+{u}_{j}$$

where, *Y*_*ij*_ represents the observed count of speeding events for road link *i* in city *j*, while *E*(*Y*_*ij*_) denotes its expected count. The term GPS_*ij*_ refers to the total number of GPS observations recorded on that road link, and $\log ({\mathrm{GPS}}_{ij})$ is included as an offset with its coefficient constrained to one, allowing the model to be interpreted in terms of rates. The intercept is given by *β*_0_, while *β*_1_*X*_1*ij*_ captures the fixed effect of posted speed limits and *β*_2_*X*_2*ij*_ represents additional link-level and network covariates. The term ${u}_{j} \sim N(0,{\sigma }_{u}^{2})$ is a city-level random intercept that accounts for unobserved heterogeneity across cities. Under this specification, *Y*_*ij*_ is assumed to follow a negative binomial distribution, and the model can equivalently be expressed as a rate model by normalizing the expected count by GPS_*ij*_.

Binary variables, including engineering features and road direction, were coded as indicator variables taking values of zero or one. All continuous variables were standardised by mean-centering and scaling by their standard deviation using the scale() function, resulting in variables with mean zero and unit variance. This transformation facilitates model convergence and enables meaningful comparison of coefficient magnitudes across continuous predictors within the same model. In the exposure-adjusted negative binomial models with a GPS offset, coefficients for binary variables represent the relative difference in speeding rates—that is, exposure-normalised speeding events per GPS observation—between the presence and absence of a given feature, conditional on observed GPS exposure. Coefficients for standardised continuous variables reflect the expected change in the speeding rate associated with a one-standard-deviation increase in the predictor. For categorical variables, such as posted speed limit categories, coefficients are interpreted relative to a designated reference group.

To aid interpretation, results are reported as incidence rate ratios (IRRs), obtained by exponentiating the estimated coefficients from the negative binomial models. In the exposure-adjusted models, IRRs quantify the multiplicative change in the expected rate of speeding events per GPS observation associated with a one-unit change in the predictor, holding other variables constant. An IRR equal to 1 indicates no association, values greater than 1 indicate higher speeding rates, and values less than 1 indicate lower speeding rates relative to the reference condition. Moreover, we also modelled the raw speeding count negative binomial model as a supplementary reference for link-level speeding burden, showing the IRR in Fig. S1. Throughout the paper, speeding rate refers to exposure-normalised speeding events per GPS observation, whereas speeding incident refers to the total number of observed speeding events on a road link.

### Causal robustness models

To reduce confounding in this cross-sectional observational setting, we applied propensity score-based adjustment methods for both binary and continuous explanatory variables, while recognising that these methods can only account for observed covariates. Accordingly, results are interpreted as adjusted effects consistent with plausible causal mechanisms, rather than fully identified causal effects.

For binary treatment variables (e.g. presence of traffic calming, crossing, camera, or link direction), propensity score matching (PSM) was implemented to estimate the average treatment effect on the treated (ATT). We estimated a separate propensity score model using logistic regression for each binary treatment variable. The propensity score represents the conditional probability that a road link received the focal intervention given observed covariates, including other engineering features, road geometry attributes, surrounding network structure metrics, and speed limit group. The focal treatment variable was excluded from the covariate set to avoid post-treatment adjustment. Nearest-neighbour 1:1 matching with a calliper of 0.2 standard deviations of the logit of the propensity score was applied to enforce overlap and common support. Covariate balance was assessed using standardised mean differences, with values below 0.10 taken as evidence of adequate balance.

For continuous treatment variables, such as road geometry and network connectivity measures, we applied the generalized propensity score (GPS) framework^47,48^. The GPS was estimated by modelling the conditional distribution of the standardized treatment given observed covariates. The conditional treatment model was estimated using gradient boosting decision trees (XGBoost), allowing flexible capture of nonlinear and high-dimensional relationships. Trimming was applied to the tails of both the treatment and GPS distributions to ensure adequate overlap, with thresholds iteratively adjusted based on balance diagnostics while retaining sufficient effective sample size. GPS-derived counterfactual weights were then applied to construct a pseudo-population in which covariates were approximately independent of treatment intensity. Covariate balance was assessed using absolute treatment-covariate correlations, with values below 0.10 indicating adequate balance.

For binary treatments, the ATT was estimated as the mean difference in speeding outcomes—measured by speeding rate in percentage—between matched treated and control road links; for continuous treatments, effects were summarised using the estimated exposure-response function, focusing on the expected change in speeding rate associated with a one-standard-deviation increase in the treatment variable relative to its mean (see Fig. 2). The ATT for speeding events is shown in Figure S2. Full elaboration of the modelling for ATT via the difference in speeding outcomes, incorporating a broader range of parameters, is shown in Table S17 to Table S20. All analyses rely on standard identifying assumptions for propensity score methods, including conditional unconfoundedness, sufficient overlap, and the absence of interference between road links, which may be imperfectly satisfied in a single cross-sectional design.

To further assess robustness to unmeasured confounding, we conducted a sensitivity analysis using E-values. Following propensity score adjustment (PSM for binary treatments and GPS for continuous treatments), we fitted post-adjustment negative binomial mixed-effects models separately for each treatment variable, incorporating matching or GPS-derived weights and city-level random intercepts. For binary treatments, the exponentiated treatment coefficient yields an incidence rate ratio (IRR) corresponding to the average treatment effect on the treated (ATT). For continuous treatments, the IRR as ATT represents the multiplicative change in speeding associated with a one-standard-deviation increase in the standardised exposure. Sensitivity causal models were estimated for both speeding rate (with a GPS point-exposure offset) and raw speeding count outcomes (see Figure S3 and Figure S4).

E-values were computed from these IRRs and their corresponding confidence intervals on the risk-ratio scale. The E-value quantifies the minimum strength of association that an unmeasured confounder would need to have with both the treatment and the outcome, conditional on measured covariates, to fully explain away the observed effect. These sensitivity analyses are interpreted as heuristic robustness diagnostics rather than sharp bounds on causal effects. By combining propensity score-based confounding adjustment with post-adjustment outcome modelling, this approach separates confounding control from effect estimation and enables sensitivity analysis on a well-defined multiplicative effect scale. All sensitivity causal analysis results are presented in Figure S3 and Figure S4, and Table S21 and Table S22.

## Supplementary information

Supplementary information

## Data Availability

The source is open data, available for download on the Healthy and Sustainable Places Data Service (HASP) repository at the following: 10.82147/010.

## Code availability

The code used to process these data and analyses is available at the following URL: https://github.com/geolongc/speeding_GPS_UK.

## References

[1] Wilmot, C. G. & Khanal, M. Effect of Speed limits on speed and safety: A review. *Transp. Rev.* **19**, 315–329 (1999).

[2] Aljanahi, A. A. M., Rhodes, A. H. & Metcalfe, A. V. Speed, speed limits and road traffic accidents under free flow conditions. *Accid. Anal. Prev.* **31**, 161–168 (1999). 10084631 10.1016/s0001-4575(98)00058-x

[3] Williams, A. F., Kyrychenko, S. Y. & Retting, R. A. Characteristics of speeders. *J. Saf. Res.* **37**, 227–232 (2006). 10.1016/j.jsr.2006.04.001 16872632

[4] Elliott, M. A. & Thomson, J. A. The social cognitive determinants of offending drivers’ speeding behaviour. *Accid. Anal. Prev.* **42**, 1595–1605 (2010). 20728608 10.1016/j.aap.2010.03.018

[5] Richard, C. M., Lee, J., Atkins, R. & Brown, J. L. Using SHRP2 naturalistic driving data to examine driver speeding behavior. *J. Saf. Res.* **73**, 271–281 (2020). 10.1016/j.jsr.2020.03.008 32563403

[6] Khaddar, S., Pathivada, B. K. & Perumal, V. Modeling over speeding behavior of vehicles using a random parameter negative binomial approach: A case study of Mumbai, India. *Transportation Res. Interdiscip. Perspect.* **18**, 100790 (2023).

[7] Fondzenyuy, S. K. et al. The Impact of Speed Limit Change on Emissions: A Systematic Review of Literature. *Sustainability* **16**, 7712 (2024).

[8] Tapp, A., Nancarrow, C. & Davis, A. Support and compliance with 20 mph speed limits in Great Britain. *Transportation Res. Part F: Traffic Psychol. Behav.* **31**, 36–53 (2015).

[9] Elvik, R. Speed Limits, Enforcement, and Health Consequences. *Annu. Rev. Public Health* **33**, 225–238 (2012). 22224882 10.1146/annurev-publhealth-031811-124634

[10] Soole, D. W., Watson, B. C. & Fleiter, J. J. Effects of average speed enforcement on speed compliance and crashes: A review of the literature. *Accid. Anal. Prev.* **54**, 46–56 (2013). 23474237 10.1016/j.aap.2013.01.018

[11] Antic, B., Pešic, D., Vujanic, M. & Lipovac, K. The influence of speed bumps heights to the decrease of the vehicle speed – Belgrade experience. *Saf. Sci.* **57**, 303–312 (2013).

[12] Fwa, T. F. & Tan, L. S. Geometric Characterization of Road Humps for Speed-Control Design. *J. Transportation Eng.* **118**, 593–598 (1992).

[13] Gitelman, V., Carmel, R., Pesahov, F. & Chen, S. Changes in road-user behaviors following the installation of raised pedestrian crosswalks combined with preceding speed humps, on urban arterials. *Transportation Res. Part F: Traffic Psychol. Behav.* **46**, 356–372 (2017).

[14] Damsere-Derry, J. et al. Evaluation of the effectiveness of traffic calming measures on vehicle speeds and pedestrian injury severity in Ghana. *Traffic Inj. Prev.* **20**, 336–342 (2019). 31033340 10.1080/15389588.2019.1581925 PMC7141770

[15] Zaidel, D., Hakkert, A. S. & Pistiner, A. H. The use of road humps for moderating speeds on Urban streets. *Accid. Anal. Prev.* **24**, 45–56 (1992). 1547013 10.1016/0001-4575(92)90071-p

[16] Jägerbrand, A. K., Johansson, M. & Laike, T. Speed Responses to Speed Humps as Affected by Time of Day and Light Conditions on a Residential Road with Light-Emitting Diode (LED) Road Lighting. *Safety* **4**, 10 (2018).

[17] Yeo, J., Lee, J., Cho, J., Kim, D.-K. & Jang, K. Effects of speed humps on vehicle speed and pedestrian crashes in South Korea. *J. Saf. Res.* **75**, 78–86 (2020). 10.1016/j.jsr.2020.08.003 33334495

[18] Agerholm, N., Knudsen, D. & Variyeswaran, K. Speed-calming measures and their effect on driving speed – Test of a new technique measuring speeds based on GNSS data. *Transportation Res. Part F: Traffic Psychol. Behav.* **46**, 263–270 (2017).

[19] Pau, M. & Angius, S. Do speed bumps really decrease traffic speed? An Italian experience. *Accid. Anal. Prev.* **33**, 585–597 (2001). 11491239 10.1016/s0001-4575(00)00070-1

[20] Salau, T. A. O., Adeyefa, A. O. & Oke, S. A. Vehicle speed control using road bumps. *Transport* **19**, 130–136 (2004).

[21] Kveladze, I. & Agerholm, N. Visual analysis of speed bumps using floating car dataset. *J. Locat. Based Serv.* **12**, 119–139 (2018).

[22] Lee, G., Joo, S., Oh, C. & Choi, K. An evaluation framework for traffic calming measures in residential areas. *Transportation Res. Part D: Transp. Environ.* **25**, 68–76 (2013).

[23] Gonzalo-Orden, H., Rojo, M., Pérez-Acebo, H. & Linares, A. Traffic Calming Measures and their Effect on the Variation of Speed. *Effic., Safe Intell. Transp. Sel. Pap. XII Conf. Transp. Eng., Valencia (Spain) 7-9 June* **18**, 349–356 (2016).

[24] Afghari, A. P., Haque, M. M. & Washington, S. Applying fractional split model to examine the effects of roadway geometric and traffic characteristics on speeding behavior. *Traffic Inj. Prev.* **19**, 860–866 (2018). 30644760 10.1080/15389588.2018.1509208

[25] Bella, F. & Silvestri, M. Effects of safety measures on driver’s speed behavior at pedestrian crossings. *Accid. Anal. Prev.* **83**, 111–124 (2015). 26253423 10.1016/j.aap.2015.07.016

[26] Moreno, A. T. & García, A. Use of speed profile as surrogate measure: Effect of traffic calming devices on crosstown road safety performance. *Accid. Anal. Prev.* **61**, 23–32 (2013). 23177903 10.1016/j.aap.2012.10.013

[27] Gonzalo-Orden, H., Pérez-Acebo, H., Unamunzaga, A. L. & Arce, M. R. Effects of traffic calming measures in different urban areas. *XIII Conf. Transp. Eng., CIT2018* **33**, 83–90 (2018).

[28] Ghasemzadeh, A. & Ahmed, M. M. Quantifying regional heterogeneity effect on drivers’ speeding behavior using SHRP2 naturalistic driving data: A multilevel modeling approach. *Transportation Res. Part C: Emerg. Technol.* **106**, 29–40 (2019).

[29] Lee, Y. M., Chong, S. Y., Goonting, K. & Sheppard, E. The effect of speed limit credibility on drivers’ speed choice. *Transportation Res. Part F: Traffic Psychol. Behav.* **45**, 43–53 (2017).

[30] Yao, Y., Carsten, O., Hibberd, D. & Li, P. Exploring the relationship between risk perception, speed limit credibility and speed limit compliance. *Transportation Res. Part F: Traffic Psychol. Behav.* **62**, 575–586 (2019).

[31] Huang, Y., Sun, D. J. & Tang, J. Taxi driver speeding: Who, when, where and how? A comparative study between Shanghai and New York City. *Traffic Inj. Prev.* **19**, 311–316 (2018). 29045160 10.1080/15389588.2017.1391382

[32] Yokoo, T. & Levinson, D. Measures of speeding from a GPS-based travel behavior survey. *Traffic Inj. Prev.* **20**, 158–163 (2019). 30888884 10.1080/15389588.2018.1543873

[33] Abdel-Aty, M., Ugan, J. & Islam, Z. Exploring the influence of drivers’ visual surroundings on speeding behavior. *Accid. Anal. Prev.* **198**, 107479 (2024). 38245952 10.1016/j.aap.2024.107479

[34] Cai, Q., Abdel-Aty, M., Mahmoud, N., Ugan, J. & Al-Omari, M. M. A. Developing a grouped random parameter beta model to analyze drivers’ speeding behavior on urban and suburban arterials with probe speed data. *Accid. Anal. Prev.* **161**, 106386 (2021). 34481159 10.1016/j.aap.2021.106386

[35] Perez, M. A., Sears, E., Valente, J. T., Huang, W. & Sudweeks, J. Factors modifying the likelihood of speeding behaviors based on naturalistic driving data. *Accid. Anal. Prev.* **159**, 106267 (2021). 34186469 10.1016/j.aap.2021.106267

[36] Kong, X., Das, S., Jha, K. & Zhang, Y. Understanding speeding behavior from naturalistic driving data: Applying classification based association rule mining. *Accid. Anal. Prev.* **144**, 105620 (2020). 32570086 10.1016/j.aap.2020.105620

[37] Dalton, N. S. C.*Synergy, inteligibility and revelation in neighbourhood places*. Doctoral, UCL (University College London) https://discovery.ucl.ac.uk/id/eprint/1334117/ (2011). Publication Title: Doctoral thesis, UCL (University College London).

[38] Tatler, B. W. & Land, M. F. Vision and the representation of the surroundings in spatial memory. *Philos. Trans. R. Soc. B: Biol. Sci.* **366**, 596–610 (2011). 10.1098/rstb.2010.0188 PMC3030831 21242146

[39] Droin, A., Wurm, M. & Taubenböck, H. The Individual Walkable Neighborhood - Evaluating people-centered spatial units focusing on urban density. *Computers, Environ. Urban Syst.* **99**, 101893 (2023).

[40] Thompson Sargoni, O. & Manley, E. Neighbourhood-level pedestrian navigation using the construal level theory. *Environment and Planning B: Urban Analytics and City Science* **50** https://trid.trb.org/View/2122456 (2023).

[41] Kaparias, I., Bell, M., Biagioli, T., Bellezza, L. & Mount, B. Behavioural analysis of interactions between pedestrians and vehicles in street designs with elements of shared space. *Transportation Res. Part F: Traffic Psychol. Behav.* **30**, 115–127 (2015).

[42] Elliott, M. A., Armitage, C. J. & Baughan, C. J. Drivers’ compliance with speed limits: an application of the theory of planned behavior. *J. Appl. Psychol.* **88**, 964–972 (2003). 14516256 10.1037/0021-9010.88.5.964

[43] Wallén Warner, H. & Åberg, L. Drivers’ beliefs about exceeding the speed limits. *Transportation Res. Part F: Traffic Psychol. Behav.* **11**, 376–389 (2008).

[44] Newson, P. & Krumm, J. Hidden Markov map matching through noise and sparseness 336–343 10.1145/1653771.1653818 (2009).

[45] Tang, K. Gotrackit. https://github.com/zdsjjtTLG/TrackIt (2023). Accessed December 20, 2023.

[46] Boeing, G. OSMnx: New methods for acquiring, constructing, analyzing, and visualizing complex street networks. *Computers, Environ. Urban Syst.* **65**, 126–139 (2017).

[47] Wu, X., Mealli, F., Kioumourtzoglou, M.-A., Dominici, F. & Braun, D. Matching on Generalized Propensity Scores with Continuous Exposures. *Journal of the American Statistical Association*10.1080/01621459.2022.2144737 (2024). Publisher: Taylor & Francis. 10.1080/01621459.2022.2144737 PMC10958667 38524247

[48] Khoshnevis, N., Wu, X. & Braun, D. CausalGPS: An R Package for Causal Inference With Continuous Exposures (2023). http://arxiv.org/abs/2310.00561. ArXiv:2310.00561 [stat].

