# Supplementary material for: Geographic determinants of goods vehicle speeding in British cities
Source: NPJ Sustain Mobil Transp. 2026 Jun 3;3(1):38. doi: 10.1038/s44333-026-00106-3 (PMC13233316; doi:10.1038/s44333-026-00106-3)
Supplement: Supplementary file 1 — Supplementary information [file 44333_2026_106_MOESM1_ESM.pdf]

Supplementary Information

In the supplementary information, we present details for road link counts, total GPS points, and speeding points by speed limit, maps for speeding rates (%) and speeding events in road links across British cities, and supplementary statistical methods and results.

Supplementary information for road links, GPS points, and speeding events by speed limit in British cities

Table S1 Speeding events and road links by speed limit in British cities

Total points<br>Percent(%)<br>Speeding events<br>Percent(%)<br>Link count<br>Percent(%)	Total	20 Mph	30 Mph	40 Mph	50 Mph	60 Mph	70 Mph
London	118321296	33540011	44491799	15721247	13225655	1169652	10172932
	100	28.35	37.6	13.29	11.18	0.99	8.6
	9267597	4540560	2182272	860949	1305345	65439	313032
	100	48.99	23.55	9.29	14.09	0.71	3.38
	263075	144383	109921	5880	2153	402	336
	100	54.88	41.78	2.24	0.82	0.15	0.13
Manchester	35190309	1988507	12201539	3091523	1162686	616064	16130071
	100	5.65	34.67	8.79	3.3	1.75	45.84
	2428917	413100	1170342	210971	91144	29274	514086
	100	17.01	48.18	8.69	3.75	1.21	21.17
	117584	43566	67800	3734	631	742	1111
	100	37.05	57.66	3.18	0.54	0.63	0.94
Oxford	3714943	1488944	1174686	127541	581739	56449	285584
	100	40.08	31.62	3.43	15.66	1.52	7.69
	274944	160628	47200	7368	58030	0	1718
	100	58.42	17.17	2.68	21.11	0	0.62
	7630	6318	1015	84	114	46	53
	100	82.8	13.3	1.1	1.49	0.6	0.69
Cambridge	1005794	306115	538651	69211		3674	88143
	100	30.44	53.55	6.88		0.37	8.76
	82033	46838	30210	2863		0	2122
	100	57.10	36.83	3.49		0	2.59
	5381	3858	1411	91		12	9
	100	71.7	26.22	1.69		0.22	0.17
Cardiff	3468509	166144	1001716	402783	327548	32097	1538221
	100	4.79	28.88	11.61	9.44	0.93	44.35
	241079	23260	48096	27960	62098	24	79641
	100	9.65	19.95	11.60	25.76	0.01	33.04
	12192	2589	8766	303	95	256	183
	100	21.24	71.9	2.49	0.78	2.1	1.5
West of England	17352934	1907289	4433678	2592503	1304869	1008248	6106347
	100	10.99	25.55	14.94	7.52	5.81	35.19
	1769513	489329	575235	263790	117358	5632	318169
	100	27.65	32.51	14.91	6.63	0.32	17.98
	42557	22638	14569	2075	574	2325	376
	100	53.19	34.23	4.88	1.35	5.46	0.88
West Midlands	37461592	1796133	15306186	5864867	1766415	416035	12311956
	100	4.79	40.86	15.66	4.72	1.11	32.87
	2674793	434674	1511094	408475	226932	3955	89663
	100	16.25	56.49	15.27	8.48	0.15	3.35
	114221	18421	88516	5673	781	346	484
	100	16.13	77.5	4.97	0.68	0.3	0.42
Liverpool	21430620	1341731	8235624	3696994	610078	1256790	6289403
	100	6.26	38.43	17.25	2.85	5.86	29.35
	1971589	316514	1010836	259982	51200	57878	275179
	100	16.05	51.27	13.19	2.60	2.94	13.96
	70435	27407	38339	2997	380	809	503
	100	38.91	54.43	4.25	0.54	1.15	0.71
West Yorkshire	38685062	1495379	11393316	4574270	1728737	1251314	18242046
	100	3.87	29.45	11.82	4.47	3.23	47.16
	2597756	353354	1141149	319886	198701	6450	578216
	100	13.60	43.93	12.31	7.65	0.25	22.26
	107639	30177	66746	5822	1038	2979	877
	100	28.04	62.01	5.41	0.96	2.77	0.81
South Yorkshire	23629615	346458	4333109	2758890	1094450	2021846	13074862
	100	1.47	18.34	11.68	4.63	8.56	55.33
	1289514	100502	535445	204360	88843	9701	350663
	100	7.79	41.52	15.85	6.89	0.75	27.19
	55446	7375	38534	4880	706	3408	543
	100	13.3	69.5	8.8	1.27	6.15	0.98
Newcastle	7961628	657383	2590069	767855	1223020	355236	2368065
	100	8.26	32.53	9.64	15.36	4.46	29.74
	677586	152584	307932	44460	127975	1058	43577
	100	22.52	45.45	6.56	18.89	0.16	6.43
	45255	16021	24856	1866	761	843	908
	100	35.4	54.92	4.12	1.68	1.86	2.01
Edinburgh	8754424	2133474	1543827	1147975	307671	55548	3565929
	100	24.37	17.63	13.11	3.51	0.63	40.73
	899431	460807	197663	114279	65358	515	60809
	100	51.23	21.98	12.71	7.27	0.06	6.76
	19585	14633	3665	723	114	252	198
	100	74.72	18.71	3.69	0.58	1.29	1.01
Glasgow	13661728	673811	6129993	672077	3628321	732033	1825493
	100	4.93	44.87	4.92	26.56	5.36	13.36
	2194105	106673	607994	53502	1249323	114984	61629
	100	4.86	27.71	2.44	56.94	5.24	2.81
	31934	4522	26163	637	377	131	104
	100	14.16	81.93	1.99	1.18	0.41	0.33

Note: Road links in Cambridge do not have a posted speed limit at 50 mph

Supplementary link-level speeding count by speed limit in British cities

Table S2 Speeding events by speed limit group, per link

	Total	20 Mph	30 Mph	40 Mph	50 Mph	60 Mph	70 Mph
London	35.23	31.45	19.85	146.42	606.29	162.78	931.64
Manchester	20.66	9.48	17.26	56.5	144.44	39.45	462.72
Oxford	36.03	25.42	46.5	87.71	509.04	0.00	32.42
Cambridge	15.24	12.14	21.41	31.46		0.00	235.78
Cardiff	19.77	8.98	5.49	92.28	653.66	0.09	435.2
West of England	41.58	21.62	39.48	127.13	204.46	2.42	846.19
West Midlands	23.42	23.6	17.07	72	290.57	11.43	185.25
Liverpool	27.99	11.55	26.37	86.75	134.74	71.54	547.08
West Yorkshire	24.13	11.71	17.1	54.94	191.43	2.17	659.31
South Yorkshire	23.26	13.63	13.9	41.88	125.84	2.85	645.79
Newcastle	14.97	9.52	12.39	23.83	168.17	1.26	47.99
Edinburgh	45.92	31.49	53.93	158.06	573.32	2.04	307.12
Glasgow	68.71	23.59	23.24	83.99	3313.85	877.74	592.59

Note: 20 mph includes road links with posted speed limits at 5, 10, 15, and 20 mph. Speed limits are reported in miles per hour (mph); 20, 30, 40, 50, 60, and 70 mph correspond approximately to 32, 48, 64, 80, 97, and 113 km/h, respectively.

Supplementary Model Results

Negative binomial model using raw speeding counts

The study also incorporates a raw speeding counts model as a reference to model the expected speeding events per road link. Even if the expected speeding rate is identical, roads with more traffic volume represented by sampled total GPS points can naturally be expected to have more speeding activity. However, traffic volume, road characteristics, and speeding propensity are jointly determined, which can undermine such assumptions. For instance, collinearity exists between total GPS points and other covariates such as link length and road type. This raw count model is not interpreted causally but is used to assess the robustness of spatial patterns in overall speeding burden. The mixed-effects negative binomial model for raw speeding events is articulated as follows:

$$\log(E(Y_{ij})) = \beta_0 + \beta_1 X_{1ij} + \beta_2 X_{2ij} + u_j \tag{1}$$

where, Y_{ij} denotes the observed count of speeding events for road link i in city j, and $E(Y_{ij})$ represents the expected number of speeding events, with $\log(E(Y_{ij}))$ being its logarithmic transformation. The intercept is given by β_0, while $\beta_1 X_{1ij}$ captures the fixed effect of speed limits and $\beta_2 X_{2ij}$ represents additional covariates. The term $u_j \sim N(0, \sigma_u^2)$ is a city-level random effect accounting for unobserved heterogeneity across cities. Under this model, Y_{ij} is assumed to follow a negative binomial distribution.

Binary variables, including engineering features and road direction, were coded as indicator variables taking values of zero or one. All continuous variables were standardised by mean-centering and scaling by their standard deviation using the scale() function, resulting in variables with mean zero and unit variance. This transformation facilitates model convergence and enables meaningful comparison of coefficient magnitudes across continuous predictors within the same model. Coefficients from the raw-count models represent differences in the expected number of speeding events per road link, capturing variation in overall speeding incidence that reflects the combined influence of road characteristics, traffic volume, and GPS sampling intensity rather than exposure-normalised speeding propensity.

To aid interpretation, results are reported as incidence rate ratios (IRRs), obtained by exponentiating the estimated coefficients from the negative binomial models. IRRs from the raw-count models represent multiplicative differences in the expected number of speeding events per road link, reflecting combined effects of road characteristics, traffic volume, and sampling intensity rather than exposure-normalised speeding propensity. An IRR equal to 1 indicates no association, values greater than 1 indicate higher speeding occurrence, and values less than 1 indicate lower occurrence relative to the reference condition (see Figure S1).

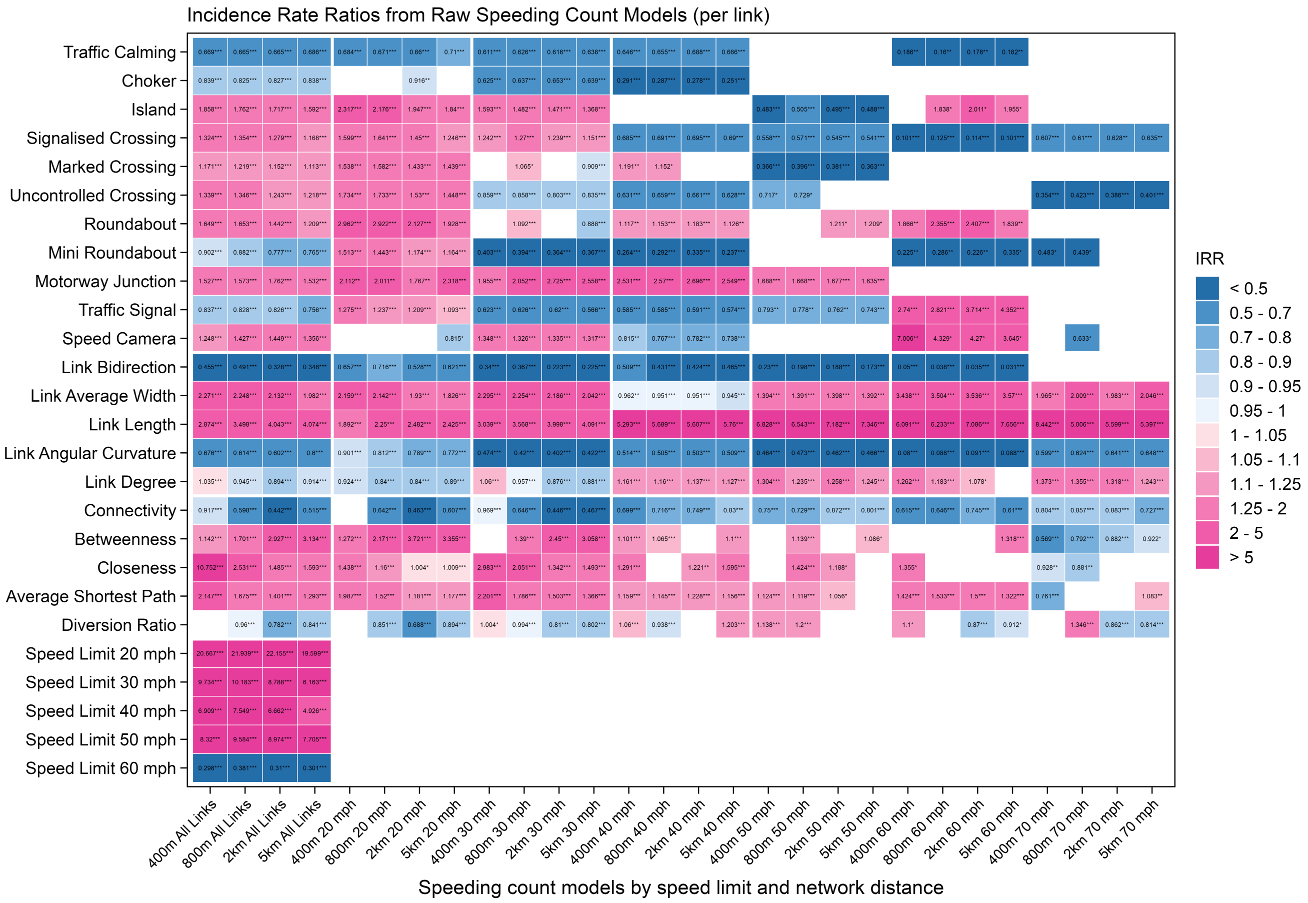

Fig. S1 Colour grids show the Incidence Rate Ratio for independent variables across raw count negative binomial models for road links with varying speed limits and network distances. Scores above 1 indicate positive association with speeding counts per link, scores lower than 1 indicate negative associations with speeding. Network-based independent variables including connectivity, betweenness, closeness, average shortest path, and diversion ratio are adjusted for distance thresholds at 400, 800, 2k, and 5k metres. Speed limits are reported in miles per hour (mph); 20, 30, 40, 50, 60, and 70 mph correspond approximately to 32, 48, 64, 80, 97, and 113 km/h, respectively.

Causal model

Moreover, in causal PSM method for binary treatments, the ATT was estimated as the mean difference in speeding outcomes—measured by raw speeding counts—between matched treated and control road links; for continuous treatments in GPS method, effects were summarised using the estimated exposure–response function, focusing on the expected change in speeding associated with a one–standard-deviation increase in the treatment variable relative to its mean (see Figure S2). All analyses rely on standard identifying assumptions for propensity score methods, including conditional unconfoundedness, sufficient overlap, and the absence of interference between road links, which may be imperfectly satisfied in a single cross-sectional design.

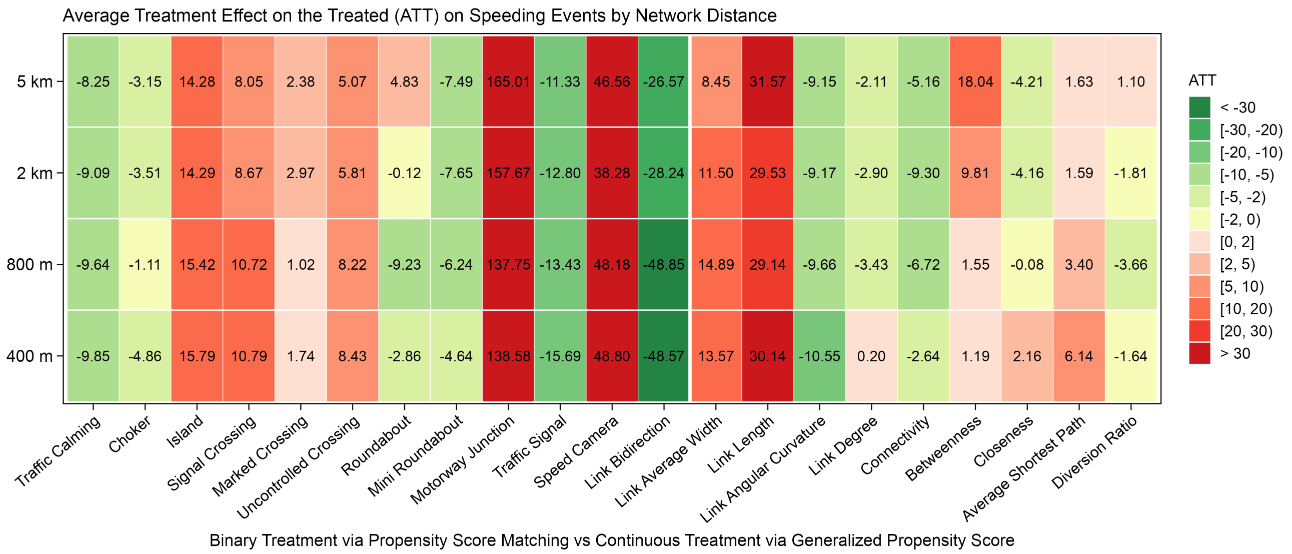

Fig. S2 Colour grids show the Average Treatment Effect on the Treated (ATT) via differences of speeding events for treatment factors across four spatial scales, where positive values indicate increases in speeding events and negative values relate to reductions in speeding events; interpretation of values differs between binary (difference between mean of treatment group and control group) and continuous variables (difference between treatment average and one standard deviation increment from exposure-response function). Speed limits are reported in miles per hour (mph); 20, 30, 40, 50, 60, and 70 mph correspond approximately to 32, 48, 64, 80, 97, and 113 km/h, respectively.

Sensitivity causal model

To further assess robustness to unmeasured confounding, we conducted a sensitivity analysis using E-values. Following propensity score adjustment (PSM for binary treatments and GPS for continuous treatments), we fitted post-adjustment negative binomial mixed-effects models separately for each treatment variable, incorporating matching or GPS-derived weights and city-level random intercepts. For binary treatments, the exponentiated treatment coefficient yields an incidence rate ratio (IRR) corresponding to the average treatment effect on the treated (ATT). For continuous treatments, the IRR represents the multiplicative change in speeding associated with a one–standard-deviation increase in the standardised exposure. Sensitivity causal models were estimated for both speeding rate (with a GPS point-exposure offset) and raw speeding count outcomes. All sensitivity causal analysis results are presented in Figure S3 and Figure S4, and Table S21 and Table S22.

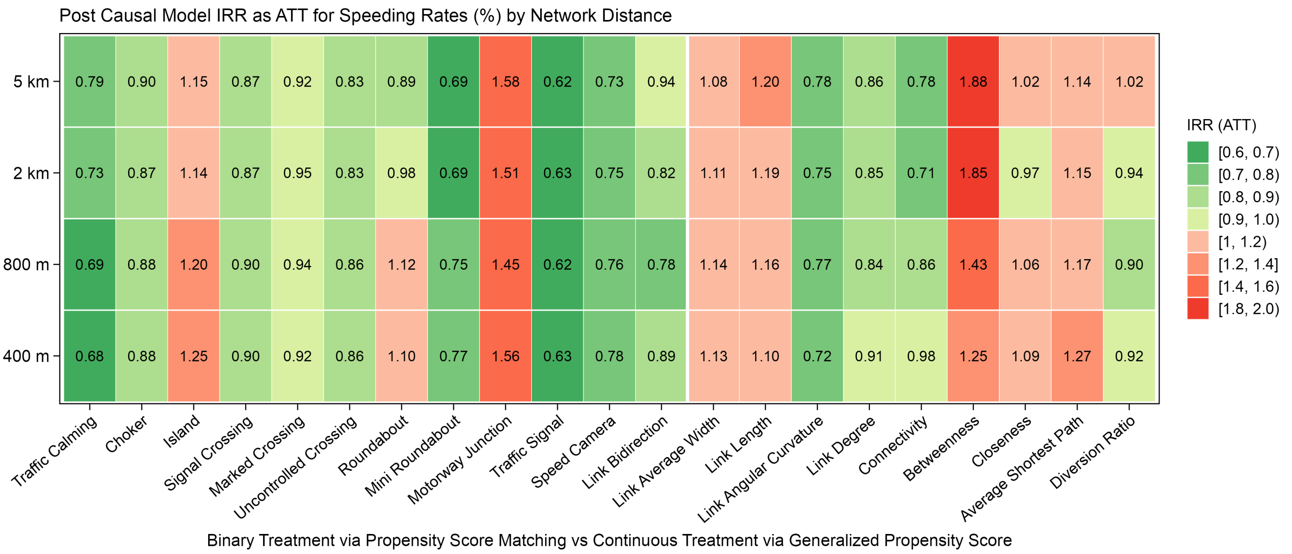

Fig. S3 Reported estimates are incidence rate ratios (IRR) as ATT obtained from post-adjustment negative binomial mixed-effects models fitted after propensity score adjustment. Binary treatments were adjusted using propensity score matching (PSM), and continuous treatments using generalized propensity score (GPS) weighting. All post-adjustment models incorporate matching or GPS-derived weights and city-level random intercepts. For binary treatments, the IRR represents the average treatment effect on the treated (ATT). For continuous treatments, the IRR corresponds to the multiplicative change in speeding associated with a one–standard-deviation increase in the standardized exposure. Results are reported across 400 m, 800 m, 2 km, and 5 km distance bands.

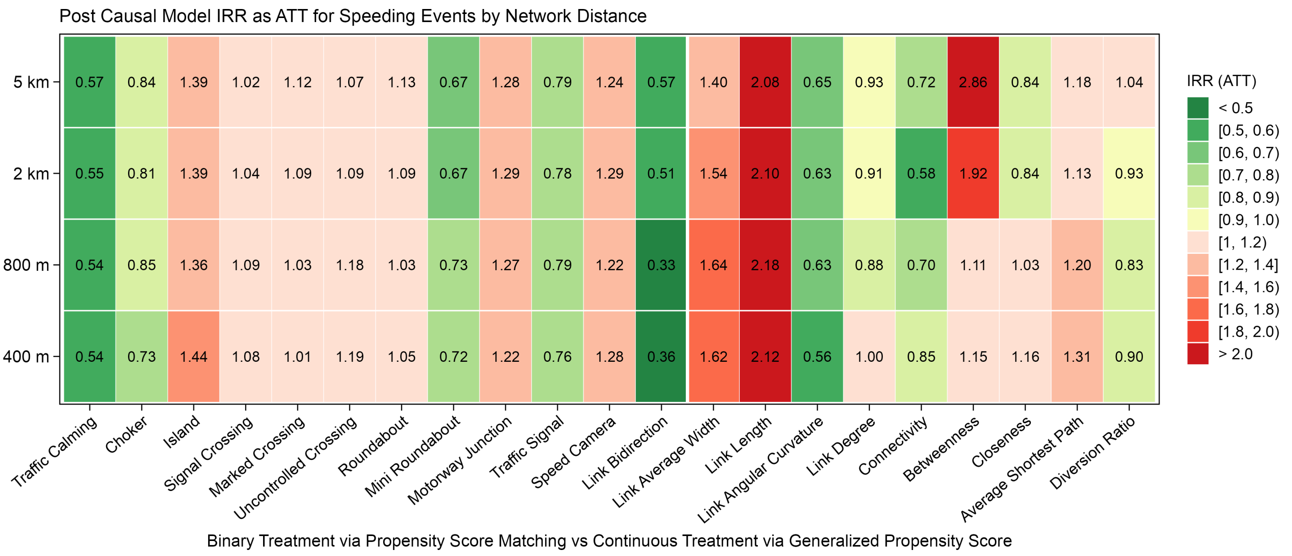

Fig. S4 Reported estimates are incidence rate ratios (IRR) as ATT obtained from post-adjustment negative binomial mixed-effects models fitted after propensity score adjustment. Binary treatments were adjusted using propensity score matching (PSM), and continuous treatments using generalized propensity score (GPS) weighting. All post-adjustment models incorporate matching or GPS-derived weights and city-level random intercepts. For binary treatments, the IRR represents the average treatment effect on the treated (ATT). For continuous treatments, the IRR corresponds to the multiplicative change in speeding associated with a one–standard-deviation increase in the standardized exposure. Results are reported across 400 m, 800 m, 2 km, and 5 km distance bands.

Supplementary Figures

Speeding rates (%) of road links in British cities

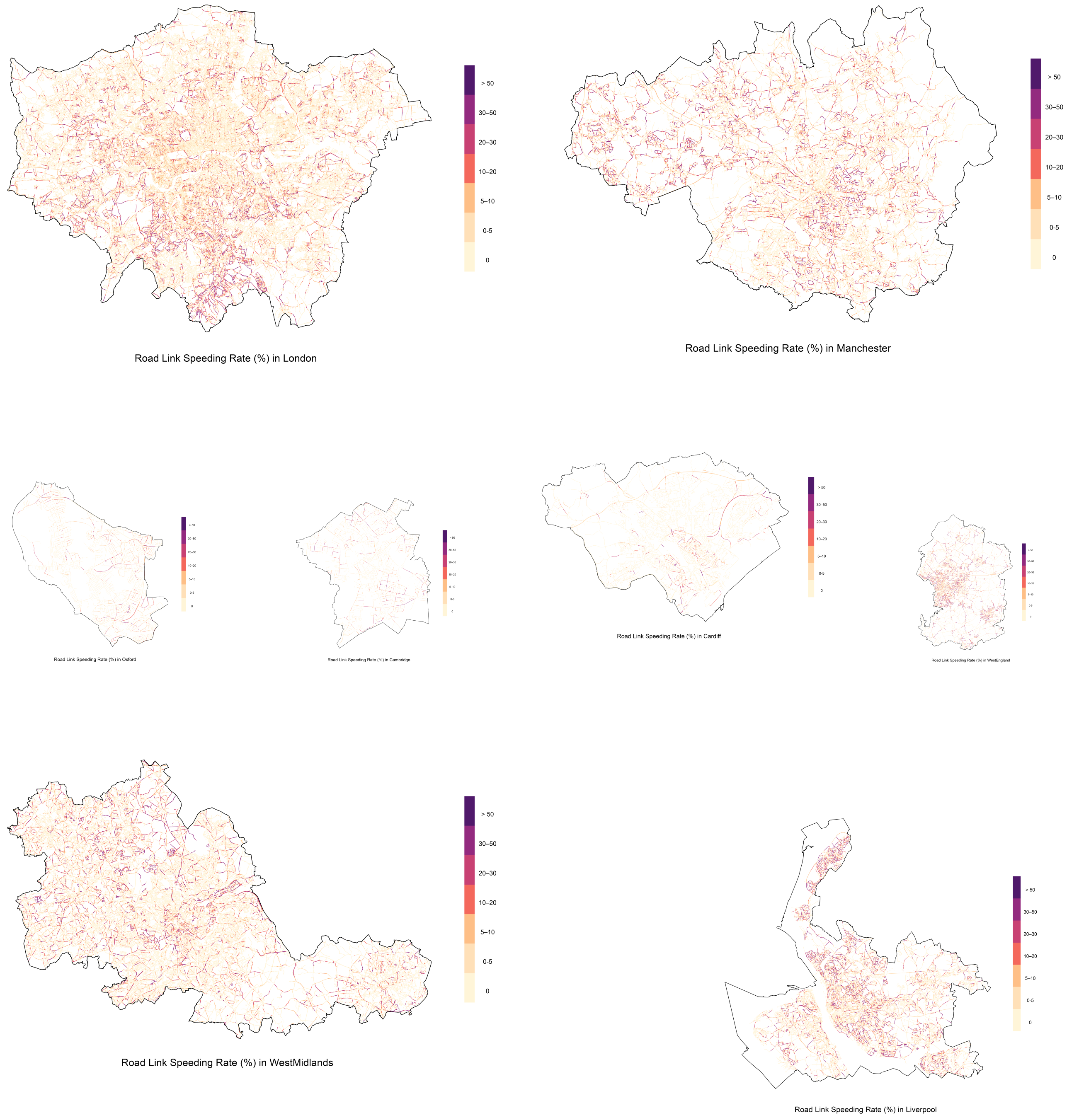

Fig. S5 Speeding rates (%) at road link level in British cities. Note that road mapping only includes road links with matched GPS point more than 5. There are 871524 road links used in plots. For the rate bands in percent of 0, (0,5], (5-10], (10-20], (20-30], (30-50], (50, 100], there are 420295, 164492, 81463, 87815, 41932, 45731, 29796 road links, respectively, in British cities

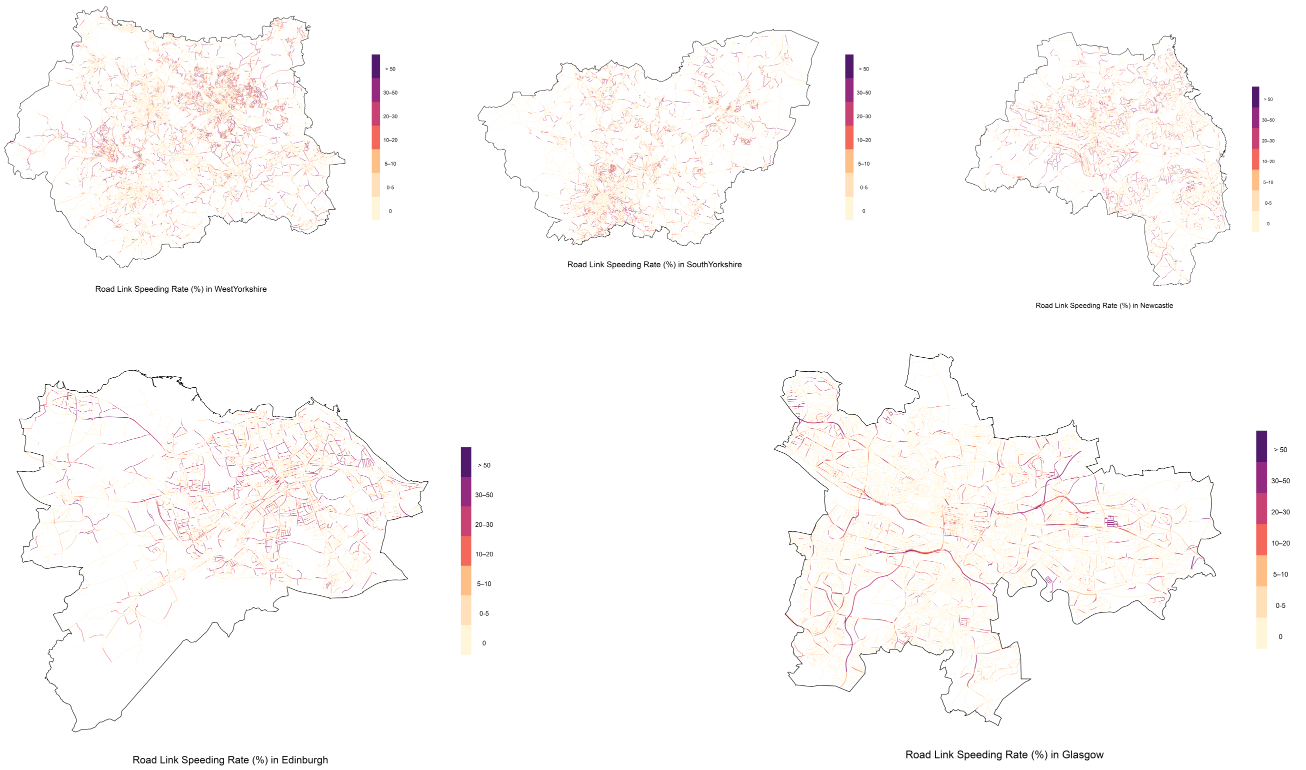

Fig. S6 Speeding rates (%) at road link level in British cities. Note that road mapping only includes road links with matched GPS point more than 5. There are 871524 road links used in plots. For the rate bands in percent of 0, (0,5], (5-10], (10-20], (20-30], (30-50], (50, 100], there are 420295, 164492, 81463, 87815, 41932, 45731, 29796 road links, respectively, in British cities

Speeding events on road links in British cities

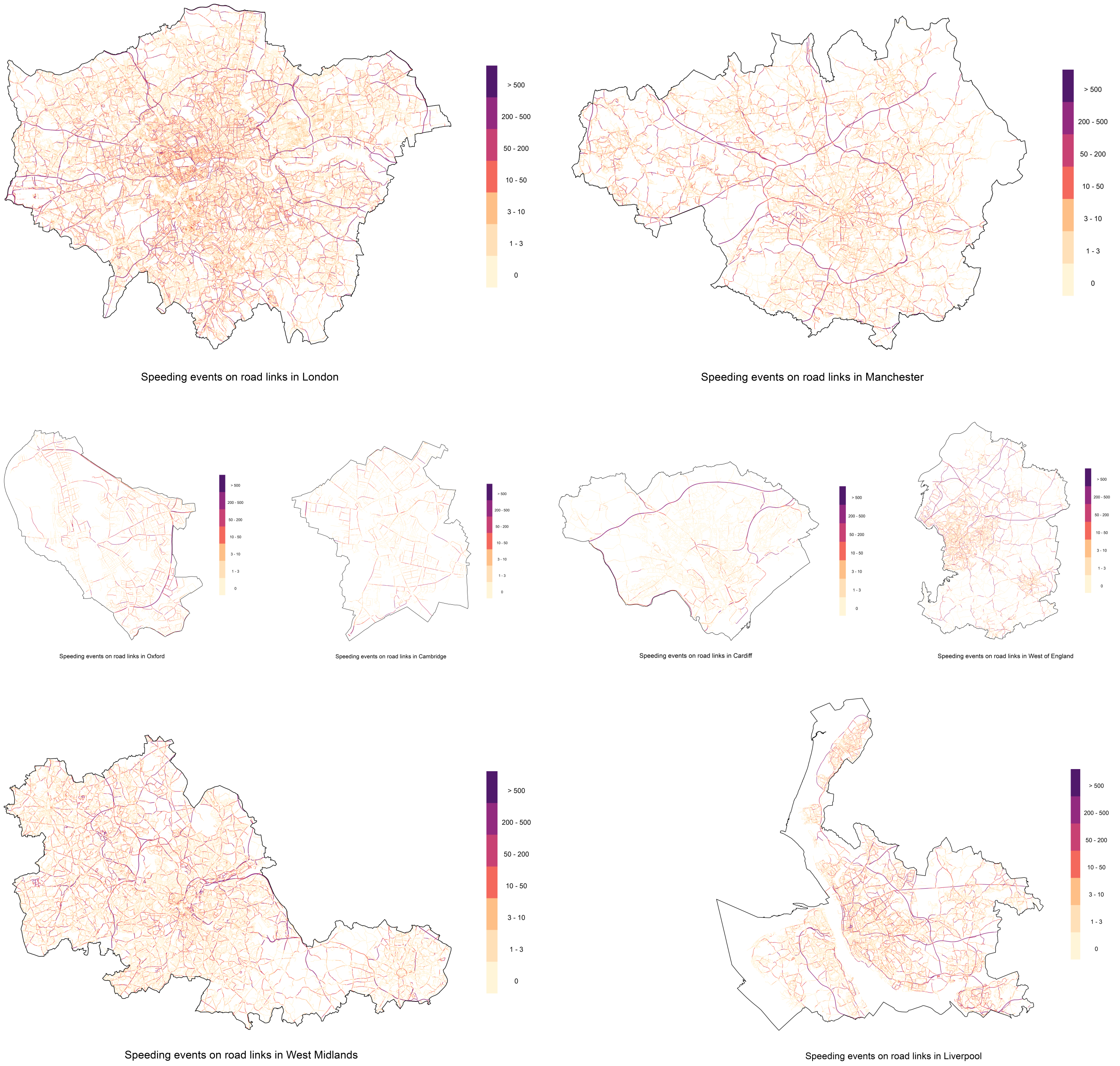

Fig. S7 Speeding events at road link level in British cities. Note that road mapping only includes road links with matched GPS point more than 5. There are 871524 road links used in plots. For the bands of 0, [1,3), [3-10), [10-50), [50-200), [200-500), [500, +), there are 420252, 122854, 123341, 125870, 55687, 14908, 8612 road links, respectively, in British cities

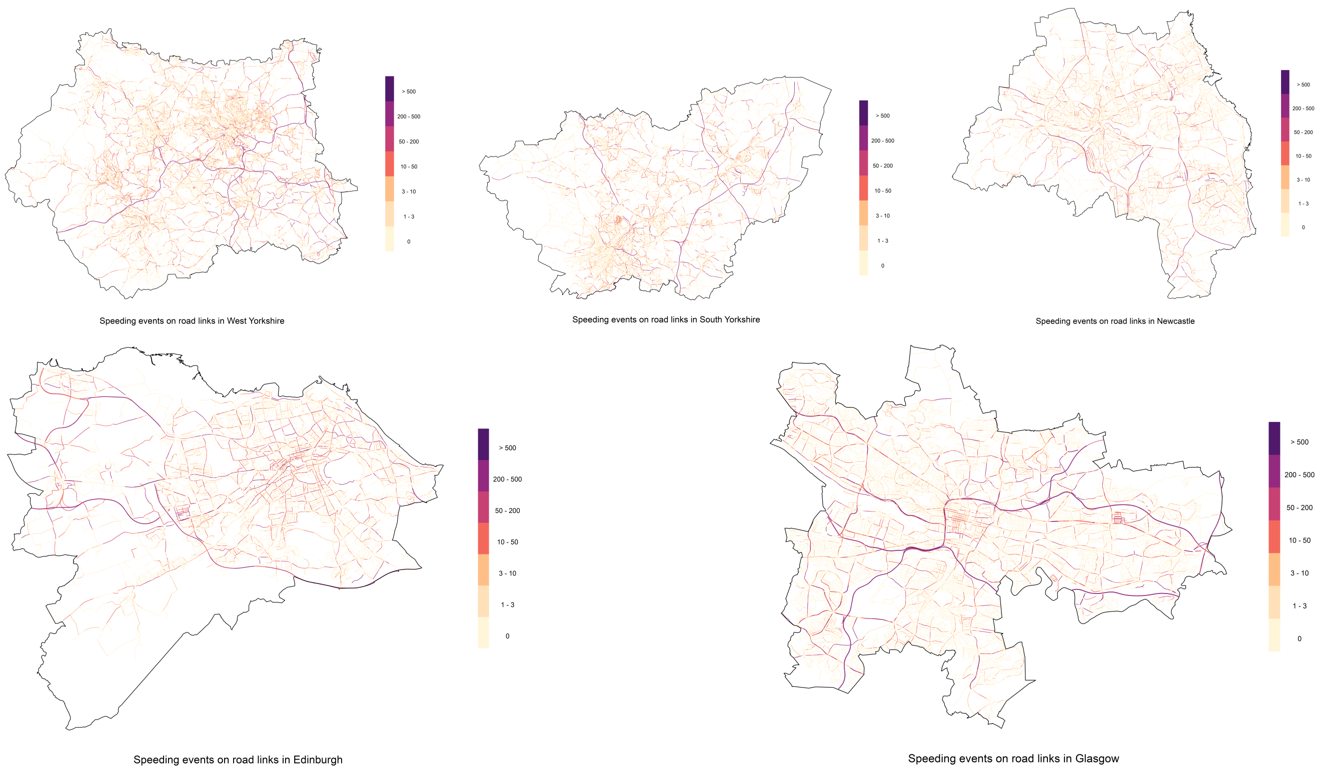

Fig. S8 Speeding events at road link level in British cities. Note that road mapping only includes road links with matched GPS point more than 5. There are 871524 road links used in plots. For the bands of 0, [1,3), [3-10), [10-50), [50-200), [200-500), [500, +), there are 420252, 122854, 123341, 125870, 55687, 14908, 8612 road links, respectively, in British cities

Supplementary Tables

Exposure-adjusted negative binomial rate model

Table S3 Exposure-adjusted negative binomial count model results for speeding events per GPS point on all road links, with network metrics measured at different network distances (400 m, 800 m, 2 km, and 5 km)

	Speeding events per GPS point (Coefficients, 95% CI, p-value, IRR, 95% CI)			
Independent variables	50m_400m	50m_800m	50m_2km	50m_5km
Traffic Calming	-0.368 (-0.380, -0.356), 0.000***, 0.692 (0.684, 0.700)	-0.353 (-0.365, -0.341), 0.000***, 0.703 (0.694, 0.711)	-0.321 (-0.333, -0.309), 0.000***, 0.726 (0.717, 0.734)	-0.295 (-0.307, -0.283), 0.000***, 0.744 (0.736, 0.753)
Choker	-0.163 (-0.204, -0.121), 0.000***, 0.850 (0.815, 0.886)	-0.162 (-0.204, -0.120), 0.000***, 0.850 (0.816, 0.887)	-0.155 (-0.197, -0.114), 0.000***, 0.856 (0.821, 0.893)	-0.139 (-0.180, -0.097), 0.000***, 0.870 (0.835, 0.907)
Island	0.305 (0.289, 0.321), 0.000***, 1.357 (1.335, 1.378)	0.262 (0.246, 0.277), 0.000***, 1.299 (1.279, 1.320)	0.212 (0.196, 0.228), 0.000***, 1.236 (1.217, 1.256)	0.163 (0.147, 0.178), 0.000***, 1.177 (1.158, 1.195)
Signalised Crossing	-0.083 (-0.098, -0.067), 0.000***, 0.921 (0.907, 0.935)	-0.073 (-0.088, -0.057), 0.000***, 0.930 (0.916, 0.944)	-0.104 (-0.120, -0.089), 0.000***, 0.901 (0.887, 0.915)	-0.147 (-0.162, -0.132), 0.000***, 0.863 (0.850, 0.877)
Marked Crossing	-0.115 (-0.139, -0.090), 0.000***, 0.892 (0.870, 0.914)	-0.098 (-0.122, -0.073), 0.000***, 0.907 (0.885, 0.929)	-0.132 (-0.157, -0.108), 0.000***, 0.876 (0.855, 0.897)	-0.158 (-0.182, -0.134), 0.000***, 0.854 (0.834, 0.875)
Uncontrolled Crossing	-0.127 (-0.145, -0.109), 0.000***, 0.881 (0.865, 0.896)	-0.130 (-0.148, -0.113), 0.000***, 0.878 (0.862, 0.893)	-0.191 (-0.208, -0.173), 0.000***, 0.826 (0.812, 0.841)	-0.202 (-0.220, -0.185), 0.000***, 0.817 (0.803, 0.831)
Roundabout	0.091 (0.069, 0.114), 0.000***, 1.095 (1.071, 1.120)	0.086 (0.064, 0.109), 0.000***, 1.090 (1.066, 1.115)	-0.010 (-0.032, 0.013), 0.401, 0.991 (0.969, 1.013)	-0.121 (-0.143, -0.099), 0.000***, 0.886 (0.867, 0.906)
Mini Roundabout	-0.364 (-0.388, -0.340), 0.000***, 0.695 (0.678, 0.712)	-0.384 (-0.408, -0.360), 0.000***, 0.681 (0.665, 0.698)	-0.447 (-0.472, -0.423), 0.000***, 0.639 (0.624, 0.655)	-0.464 (-0.488, -0.439), 0.000***, 0.629 (0.614, 0.644)
Motorway Junction	0.346 (0.290, 0.403), 0.000***, 1.414 (1.336, 1.496)	0.354 (0.297, 0.410), 0.000***, 1.424 (1.346, 1.507)	0.437 (0.381, 0.492), 0.000***, 1.547 (1.463, 1.636)	0.420 (0.364, 0.476), 0.000***, 1.522 (1.439, 1.609)
Traffic Signal	-0.566 (-0.581, -0.552), 0.000***, 0.568 (0.559, 0.576)	-0.567 (-0.581, -0.552), 0.000***, 0.567 (0.559, 0.576)	-0.569 (-0.584, -0.554), 0.000***, 0.566 (0.558, 0.574)	-0.600 (-0.615, -0.586), 0.000***, 0.549 (0.541, 0.557)
Speed Camera	-0.187 (-0.242, -0.132), 0.000***, 0.830 (0.785, 0.876)	-0.172 (-0.227, -0.118), 0.000***, 0.842 (0.797, 0.889)	-0.183 (-0.237, -0.129), 0.000***, 0.833 (0.789, 0.879)	-0.214 (-0.268, -0.160), 0.000***, 0.807 (0.765, 0.852)
Link Bidirection	-0.411 (-0.424, -0.399), 0.000***, 0.663 (0.655, 0.671)	-0.364 (-0.377, -0.352), 0.000***, 0.695 (0.686, 0.703)	-0.562 (-0.575, -0.550), 0.000***, 0.570 (0.563, 0.577)	-0.615 (-0.628, -0.603), 0.000***, 0.540 (0.534, 0.547)
Link Average Width	0.228 (0.223, 0.232), 0.000***, 1.256 (1.250, 1.262)	0.219 (0.214, 0.224), 0.000***, 1.245 (1.239, 1.251)	0.198 (0.193, 0.202), 0.000***, 1.219 (1.213, 1.224)	0.177 (0.172, 0.182), 0.000***, 1.194 (1.188, 1.199)
Link Length	0.132 (0.126, 0.138), 0.000***, 1.141 (1.135, 1.148)	0.233 (0.228, 0.238), 0.000***, 1.262 (1.255, 1.269)	0.327 (0.322, 0.332), 0.000***, 1.387 (1.380, 1.394)	0.347 (0.342, 0.352), 0.000***, 1.415 (1.408, 1.422)
Link Angular Curvature	-0.227 (-0.232, -0.222), 0.000***, 0.797 (0.793, 0.801)	-0.280 (-0.285, -0.276), 0.000***, 0.755 (0.752, 0.759)	-0.334 (-0.339, -0.330), 0.000***, 0.716 (0.712, 0.719)	-0.351 (-0.356, -0.346), 0.000***, 0.704 (0.701, 0.707)
Link Degree	-0.022 (-0.027, -0.018), 0.000***, 0.978 (0.974, 0.982)	-0.056 (-0.060, -0.052), 0.000***, 0.946 (0.942, 0.949)	-0.070 (-0.073, -0.066), 0.000***, 0.933 (0.929, 0.936)	-0.071 (-0.075, -0.067), 0.000***, 0.931 (0.928, 0.935)
Connectivity	-0.192 (-0.198, -0.185), 0.000***, 0.825 (0.820, 0.831)	-0.407 (-0.413, -0.400), 0.000***, 0.666 (0.662, 0.670)	-0.536 (-0.541, -0.530), 0.000***, 0.585 (0.582, 0.588)	-0.481 (-0.485, -0.476), 0.000***, 0.618 (0.616, 0.621)
Betweenness	0.192 (0.184, 0.199), 0.000***, 1.211 (1.202, 1.220)	0.347 (0.340, 0.355), 0.000***, 1.415 (1.405, 1.426)	0.521 (0.515, 0.527), 0.000***, 1.683 (1.673, 1.693)	0.511 (0.506, 0.517), 0.000***, 1.668 (1.659, 1.677)
Closeness	1.127 (1.091, 1.164), 0.000***, 3.088 (2.976, 3.204)	0.525 (0.481, 0.570), 0.000***, 1.691 (1.617, 1.768)	0.237 (0.211, 0.263), 0.000***, 1.267 (1.235, 1.300)	0.206 (0.181, 0.231), 0.000***, 1.229 (1.198, 1.260)
Average Shortest Path	0.477 (0.471, 0.483), 0.000***, 1.611 (1.601, 1.621)	0.368 (0.363, 0.372), 0.000***, 1.444 (1.437, 1.451)	0.266 (0.262, 0.270), 0.000***, 1.305 (1.300, 1.310)	0.208 (0.204, 0.212), 0.000***, 1.231 (1.226, 1.236)
Diversion Ratio	0.001 (-0.004, 0.005), 0.841, 1.001 (0.996, 1.006)	-0.016 (-0.021, -0.011), 0.000***, 0.984 (0.979, 0.989)	-0.105 (-0.107, -0.102), 0.000***, 0.901 (0.898, 0.903)	-0.096 (-0.100, -0.092), 0.000***, 0.908 (0.905, 0.912)
Speed Limit 20 mph	3.752 (3.705, 3.798), 0.000***, 42.594 (40.651, 44.630)	3.840 (3.793, 3.887), 0.000***, 46.528 (44.397, 48.762)	3.971 (3.923, 4.018), 0.000***, 53.020 (50.560, 55.601)	3.992 (3.945, 4.039), 0.000***, 54.174 (51.700, 56.768)
Speed Limit 30 mph	2.525 (2.479, 2.571), 0.000***, 12.491 (11.932, 13.076)	2.586 (2.541, 2.632), 0.000***, 13.283 (12.687, 13.908)	2.598 (2.551, 2.644), 0.000***, 13.433 (12.822, 14.072)	2.504 (2.458, 2.550), 0.000***, 12.230 (11.683, 12.802)
Speed Limit 40 mph	1.790 (1.743, 1.837), 0.000***, 5.987 (5.713, 6.276)	1.861 (1.814, 1.908), 0.000***, 6.432 (6.136, 6.742)	1.835 (1.787, 1.882), 0.000***, 6.265 (5.973, 6.570)	1.758 (1.711, 1.804), 0.000***, 5.798 (5.533, 6.076)
Speed Limit 50 mph	1.932 (1.876, 1.988), 0.000***, 6.903 (6.530, 7.297)	2.023 (1.968, 2.079), 0.000***, 7.562 (7.154, 7.994)	1.983 (1.927, 2.039), 0.000***, 7.266 (6.872, 7.683)	2.003 (1.948, 2.058), 0.000***, 7.412 (7.016, 7.831)
Speed Limit 60 mph	-0.655 (-0.712, -0.597), 0.000***, 0.520 (0.491, 0.550)	-0.594 (-0.651, -0.537), 0.000***, 0.552 (0.522, 0.584)	-0.770 (-0.828, -0.712), 0.000***, 0.463 (0.437, 0.491)	-0.745 (-0.801, -0.688), 0.000***, 0.475 (0.449, 0.503)
Random effect - City	0.12	0.13	0.13	0.13
ICC	0.04	0.04	0.05	0.05
Marginal R^2 / Conditional R^2	0.413 / 0.439	0.267 / 0.299	0.237 / 0.271	0.237 / 0.273

Note: This table reports results from exposure-adjusted negative binomial count models of speeding events aggregated at the road-link level for all road links. Models include the logarithm of the total number of GPS points observed on each link as an offset, thereby estimating the rate of speeding events per GPS observation. Models are estimated for all road links and stratified by posted speed limit. Network metrics (connectivity, betweenness, closeness, average shortest path, and diversion ratio) are measured at alternative network distance thresholds of 400 m, 800 m, 2 km, and 5 km, while all other covariates remain unchanged across model specifications. Results are reported as incidence rate ratios (IRR) with 95% confidence intervals and p-values. The reference category for speed-limit fixed effects is 70 mph.

Table S4 Exposure-adjusted negative binomial count model results for speeding events per GPS point on 20 mph road links, with network metrics measured at different network distances (400 m, 800 m, 2 km, and 5 km)

	Speeding events per GPS point (Coefficients, 95% CI, p-value, IRR, 95% CI)			
Independent variables	50m_400m	50m_800m	50m_2km	50m_5km
Traffic Calming	-0.300 (-0.313, -0.287), 0.000***, 0.741 (0.732, 0.751)	-0.292 (-0.306, -0.279), 0.000***, 0.746 (0.737, 0.756)	-0.274 (-0.286, -0.261), 0.000***, 0.761 (0.751, 0.771)	-0.224 (-0.237, -0.211), 0.000***, 0.800 (0.789, 0.810)
Choker	-0.058 (-0.103, -0.013), 0.012*, 0.944 (0.902, 0.987)	-0.064 (-0.109, -0.019), 0.006**, 0.938 (0.897, 0.982)	-0.103 (-0.147, -0.059), 0.000***, 0.902 (0.863, 0.943)	-0.074 (-0.118, -0.030), 0.001**, 0.929 (0.889, 0.971)
Island	0.415 (0.392, 0.438), 0.000***, 1.514 (1.480, 1.549)	0.376 (0.353, 0.398), 0.000***, 1.456 (1.423, 1.490)	0.247 (0.225, 0.270), 0.000***, 1.281 (1.252, 1.310)	0.192 (0.170, 0.215), 0.000***, 1.212 (1.185, 1.239)
Signalised Crossing	-0.033 (-0.060, -0.006), 0.015*, 0.968 (0.942, 0.994)	-0.013 (-0.040, 0.013), 0.323, 0.987 (0.961, 1.013)	-0.078 (-0.105, -0.051), 0.000***, 0.925 (0.900, 0.950)	-0.159 (-0.186, -0.132), 0.000***, 0.853 (0.830, 0.876)
Marked Crossing	0.045 (0.011, 0.079), 0.009**, 1.046 (1.011, 1.082)	0.053 (0.019, 0.087), 0.002**, 1.054 (1.019, 1.091)	0.002 (-0.031, 0.036), 0.889, 1.002 (0.969, 1.037)	-0.003 (-0.036, 0.031), 0.871, 0.997 (0.964, 1.031)
Uncontrolled Crossing	0.002 (-0.020, 0.023), 0.890, 1.002 (0.980, 1.024)	-0.006 (-0.028, 0.016), 0.588, 0.994 (0.972, 1.016)	-0.092 (-0.114, -0.071), 0.000***, 0.912 (0.892, 0.932)	-0.118 (-0.139, -0.096), 0.000***, 0.889 (0.870, 0.908)
Roundabout	0.643 (0.597, 0.689), 0.000***, 1.902 (1.816, 1.991)	0.657 (0.611, 0.703), 0.000***, 1.929 (1.842, 2.020)	0.404 (0.360, 0.449), 0.000***, 1.499 (1.433, 1.567)	0.283 (0.239, 0.328), 0.000***, 1.327 (1.270, 1.388)
Mini Roundabout	0.059 (0.028, 0.091), 0.000***, 1.061 (1.028, 1.096)	0.032 (0.000, 0.064), 0.048*, 1.033 (1.000, 1.067)	-0.091 (-0.123, -0.060), 0.000***, 0.913 (0.884, 0.942)	-0.097 (-0.128, -0.065), 0.000***, 0.908 (0.880, 0.937)
Motorway Junction	0.631 (0.320, 0.942), 0.000***, 1.880 (1.378, 2.566)	0.560 (0.250, 0.870), 0.000***, 1.750 (1.284, 2.386)	0.422 (0.120, 0.724), 0.006**, 1.525 (1.127, 2.063)	0.526 (0.223, 0.830), 0.001***, 1.693 (1.250, 2.292)
Traffic Signal	-0.255 (-0.280, -0.229), 0.000***, 0.775 (0.756, 0.795)	-0.264 (-0.290, -0.238), 0.000***, 0.768 (0.748, 0.788)	-0.307 (-0.333, -0.281), 0.000***, 0.735 (0.717, 0.755)	-0.338 (-0.364, -0.312), 0.000***, 0.713 (0.695, 0.732)
Speed Camera	-0.219 (-0.348, -0.089), 0.001***, 0.804 (0.706, 0.915)	-0.263 (-0.393, -0.134), 0.000***, 0.768 (0.675, 0.875)	-0.240 (-0.367, -0.113), 0.000***, 0.787 (0.693, 0.893)	-0.236 (-0.365, -0.108), 0.000***, 0.789 (0.694, 0.898)
Link Bidirection	-0.011 (-0.030, 0.008), 0.267, 0.989 (0.970, 1.008)	0.017 (-0.003, 0.036), 0.091, 1.017 (0.997, 1.037)	-0.152 (-0.172, -0.132), 0.000***, 0.859 (0.842, 0.876)	-0.082 (-0.100, -0.063), 0.000***, 0.922 (0.904, 0.939)
Link Average Width	0.252 (0.245, 0.258), 0.000***, 1.286 (1.278, 1.295)	0.246 (0.239, 0.252), 0.000***, 1.278 (1.270, 1.287)	0.208 (0.201, 0.214), 0.000***, 1.231 (1.223, 1.239)	0.196 (0.189, 0.202), 0.000***, 1.216 (1.209, 1.224)
Link Length	0.037 (0.030, 0.044), 0.000***, 1.038 (1.031, 1.045)	0.134 (0.127, 0.141), 0.000***, 1.143 (1.136, 1.151)	0.219 (0.212, 0.225), 0.000***, 1.245 (1.237, 1.253)	0.223 (0.216, 0.229), 0.000***, 1.249 (1.242, 1.257)
Link Angular Curvature	-0.054 (-0.061, -0.047), 0.000***, 0.948 (0.941, 0.954)	-0.121 (-0.128, -0.115), 0.000***, 0.886 (0.880, 0.892)	-0.176 (-0.182, -0.169), 0.000***, 0.839 (0.834, 0.844)	-0.216 (-0.222, -0.210), 0.000***, 0.806 (0.801, 0.811)
Link Degree	-0.088 (-0.095, -0.082), 0.000***, 0.915 (0.910, 0.921)	-0.129 (-0.135, -0.123), 0.000***, 0.879 (0.874, 0.884)	-0.120 (-0.126, -0.114), 0.000***, 0.887 (0.882, 0.892)	-0.097 (-0.102, -0.091), 0.000***, 0.908 (0.903, 0.913)
Connectivity	-0.102 (-0.112, -0.092), 0.000***, 0.903 (0.894, 0.912)	-0.306 (-0.316, -0.296), 0.000***, 0.736 (0.729, 0.744)	-0.501 (-0.508, -0.493), 0.000***, 0.606 (0.602, 0.611)	-0.440 (-0.447, -0.433), 0.000***, 0.644 (0.640, 0.648)
Betweenness	0.240 (0.229, 0.252), 0.000***, 1.272 (1.257, 1.287)	0.434 (0.423, 0.446), 0.000***, 1.544 (1.526, 1.562)	0.630 (0.621, 0.639), 0.000***, 1.877 (1.861, 1.894)	0.541 (0.534, 0.548), 0.000***, 1.718 (1.706, 1.730)
Closeness	0.234 (0.225, 0.244), 0.000***, 1.264 (1.252, 1.276)	0.197 (0.187, 0.208), 0.000***, 1.218 (1.205, 1.231)	0.230 (0.204, 0.255), 0.000***, 1.258 (1.227, 1.291)	0.016 (0.002, 0.030), 0.025*, 1.016 (1.002, 1.030)
Average Shortest Path	0.458 (0.449, 0.466), 0.000***, 1.580 (1.567, 1.594)	0.342 (0.334, 0.349), 0.000***, 1.407 (1.397, 1.418)	0.183 (0.176, 0.189), 0.000***, 1.200 (1.193, 1.208)	0.152 (0.146, 0.157), 0.000***, 1.164 (1.157, 1.170)
Diversion Ratio	-0.002 (-0.010, 0.005), 0.491, 0.998 (0.991, 1.005)	-0.041 (-0.053, -0.029), 0.000***, 0.960 (0.948, 0.972)	-0.216 (-0.225, -0.206), 0.000***, 0.806 (0.799, 0.814)	-0.116 (-0.123, -0.109), 0.000***, 0.890 (0.885, 0.896)
Random effect - City	0.12	0.12	0.12	0.13
ICC	0.05	0.05	0.05	0.05
Marginal R^2 / Conditional R^2	0.133 / 0.175	0.132 / 0.175	0.189 / 0.228	0.159 / 0.203

Note: This table reports results from exposure-adjusted negative binomial count models of speeding events aggregated at the road-link level for roads with a posted speed limit of 20 mph. Models include the logarithm of the total number of GPS points observed on each link as an offset, thereby estimating the rate of speeding events per GPS observation. Network metrics (connectivity, betweenness, closeness, average shortest path, and diversion ratio) are measured at alternative network distance thresholds of 400 m, 800 m, 2 km, and 5 km, while all other covariates remain unchanged across model specifications. Results are reported as incidence rate ratios (IRR) with 95% confidence intervals and p-values.

Table S5 Exposure-adjusted negative binomial count model results for speeding events per GPS point on 30 mph road links, with network metrics measured at different network distances (400 m, 800 m, 2 km, and 5 km)

	Speeding events per GPS point (Coefficients, 95% CI, p-value, IRR, 95% CI)			
Independent variables	50m_400m	50m_800m	50m_2km	50m_5km
Traffic Calming	-0.502 (-0.525, -0.480), 0.000***, 0.605 (0.592, 0.619)	-0.479 (-0.502, -0.456), 0.000***, 0.620 (0.606, 0.634)	-0.468 (-0.490, -0.445), 0.000***, 0.627 (0.612, 0.641)	-0.459 (-0.482, -0.437), 0.000***, 0.632 (0.617, 0.646)
Choker	-0.413 (-0.495, -0.331), 0.000***, 0.662 (0.610, 0.718)	-0.384 (-0.466, -0.302), 0.000***, 0.681 (0.627, 0.739)	-0.343 (-0.425, -0.261), 0.000***, 0.710 (0.654, 0.771)	-0.337 (-0.419, -0.254), 0.000***, 0.714 (0.657, 0.776)
Island	0.259 (0.236, 0.282), 0.000***, 1.296 (1.267, 1.325)	0.209 (0.186, 0.231), 0.000***, 1.232 (1.205, 1.260)	0.175 (0.152, 0.197), 0.000***, 1.191 (1.165, 1.218)	0.132 (0.110, 0.155), 0.000***, 1.141 (1.116, 1.167)
Signalised Crossing	-0.066 (-0.087, -0.046), 0.000***, 0.936 (0.917, 0.955)	-0.055 (-0.075, -0.035), 0.000***, 0.946 (0.927, 0.966)	-0.072 (-0.093, -0.052), 0.000***, 0.930 (0.911, 0.949)	-0.105 (-0.126, -0.085), 0.000***, 0.900 (0.881, 0.919)
Marked Crossing	-0.234 (-0.268, -0.199), 0.000***, 0.792 (0.765, 0.820)	-0.193 (-0.227, -0.158), 0.000***, 0.825 (0.797, 0.854)	-0.215 (-0.250, -0.180), 0.000***, 0.806 (0.779, 0.835)	-0.294 (-0.329, -0.259), 0.000***, 0.745 (0.720, 0.772)
Uncontrolled Crossing	-0.329 (-0.357, -0.301), 0.000***, 0.720 (0.700, 0.740)	-0.326 (-0.354, -0.298), 0.000***, 0.722 (0.702, 0.743)	-0.357 (-0.385, -0.329), 0.000***, 0.700 (0.680, 0.719)	-0.363 (-0.391, -0.335), 0.000***, 0.696 (0.677, 0.715)
Roundabout	-0.413 (-0.444, -0.382), 0.000***, 0.662 (0.641, 0.682)	-0.362 (-0.393, -0.331), 0.000***, 0.696 (0.675, 0.718)	-0.446 (-0.477, -0.416), 0.000***, 0.640 (0.621, 0.660)	-0.538 (-0.569, -0.508), 0.000***, 0.584 (0.566, 0.602)
Mini Roundabout	-0.999 (-1.035, -0.962), 0.000***, 0.368 (0.355, 0.382)	-1.006 (-1.042, -0.969), 0.000***, 0.366 (0.353, 0.379)	-1.047 (-1.084, -1.011), 0.000***, 0.351 (0.338, 0.364)	-1.063 (-1.099, -1.026), 0.000***, 0.346 (0.333, 0.358)
Motorway Junction	0.444 (0.322, 0.565), 0.000***, 1.558 (1.380, 1.760)	0.430 (0.309, 0.552), 0.000***, 1.538 (1.362, 1.736)	0.598 (0.478, 0.719), 0.000***, 1.819 (1.612, 2.052)	0.676 (0.555, 0.797), 0.000***, 1.966 (1.741, 2.220)
Traffic Signal	-0.771 (-0.790, -0.751), 0.000***, 0.463 (0.454, 0.472)	-0.757 (-0.777, -0.738), 0.000***, 0.469 (0.460, 0.478)	-0.762 (-0.782, -0.742), 0.000***, 0.467 (0.458, 0.476)	-0.813 (-0.833, -0.793), 0.000***, 0.443 (0.435, 0.452)
Speed Camera	-0.166 (-0.240, -0.093), 0.000***, 0.847 (0.787, 0.911)	-0.169 (-0.243, -0.096), 0.000***, 0.844 (0.784, 0.909)	-0.168 (-0.241, -0.095), 0.000***, 0.845 (0.786, 0.910)	-0.180 (-0.253, -0.106), 0.000***, 0.836 (0.776, 0.899)
Link Bidirection	-0.597 (-0.615, -0.579), 0.000***, 0.551 (0.541, 0.561)	-0.572 (-0.589, -0.554), 0.000***, 0.565 (0.555, 0.575)	-0.862 (-0.880, -0.843), 0.000***, 0.422 (0.415, 0.430)	-0.939 (-0.957, -0.921), 0.000***, 0.391 (0.384, 0.398)
Link Average Width	0.233 (0.226, 0.240), 0.000***, 1.262 (1.253, 1.271)	0.221 (0.214, 0.228), 0.000***, 1.247 (1.239, 1.256)	0.211 (0.205, 0.218), 0.000***, 1.235 (1.227, 1.244)	0.183 (0.177, 0.190), 0.000***, 1.201 (1.193, 1.209)
Link Length	0.187 (0.180, 0.194), 0.000***, 1.206 (1.197, 1.215)	0.282 (0.275, 0.289), 0.000***, 1.326 (1.316, 1.335)	0.370 (0.363, 0.377), 0.000***, 1.448 (1.438, 1.458)	0.405 (0.398, 0.412), 0.000***, 1.499 (1.489, 1.509)
Link Angular Curvature	-0.392 (-0.401, -0.383), 0.000***, 0.676 (0.670, 0.682)	-0.471 (-0.480, -0.463), 0.000***, 0.624 (0.619, 0.630)	-0.534 (-0.542, -0.525), 0.000***, 0.586 (0.581, 0.591)	-0.542 (-0.551, -0.534), 0.000***, 0.581 (0.576, 0.586)
Link Degree	0.003 (-0.003, 0.010), 0.290, 1.003 (0.997, 1.010)	-0.051 (-0.057, -0.045), 0.000***, 0.950 (0.945, 0.956)	-0.104 (-0.109, -0.098), 0.000***, 0.901 (0.896, 0.907)	-0.110 (-0.115, -0.104), 0.000***, 0.896 (0.891, 0.901)
Connectivity	-0.168 (-0.177, -0.158), 0.000***, 0.846 (0.838, 0.854)	-0.378 (-0.387, -0.368), 0.000***, 0.685 (0.679, 0.692)	-0.549 (-0.557, -0.541), 0.000***, 0.577 (0.573, 0.582)	-0.522 (-0.528, -0.515), 0.000***, 0.594 (0.590, 0.598)
Betweenness	0.083 (0.072, 0.093), 0.000***, 1.086 (1.075, 1.098)	0.195 (0.184, 0.205), 0.000***, 1.215 (1.202, 1.228)	0.415 (0.406, 0.424), 0.000***, 1.514 (1.501, 1.528)	0.497 (0.489, 0.505), 0.000***, 1.644 (1.631, 1.657)
Closeness	0.692 (0.670, 0.714), 0.000***, 1.998 (1.955, 2.042)	0.495 (0.444, 0.546), 0.000***, 1.641 (1.559, 1.727)	0.182 (0.153, 0.212), 0.000***, 1.200 (1.165, 1.236)	0.163 (0.135, 0.191), 0.000***, 1.177 (1.145, 1.211)
Average Shortest Path	0.538 (0.529, 0.546), 0.000***, 1.712 (1.698, 1.727)	0.444 (0.437, 0.451), 0.000***, 1.559 (1.548, 1.570)	0.332 (0.326, 0.337), 0.000***, 1.393 (1.385, 1.401)	0.249 (0.244, 0.255), 0.000***, 1.283 (1.276, 1.290)
Diversion Ratio	0.002 (-0.002, 0.006), 0.258, 1.002 (0.998, 1.006)	-0.011 (-0.013, -0.010), 0.000***, 0.989 (0.987, 0.990)	-0.116 (-0.121, -0.112), 0.000***, 0.890 (0.886, 0.894)	-0.117 (-0.123, -0.110), 0.000***, 0.890 (0.884, 0.895)
Random effect - City	0.19	0.19	0.19	0.18
ICC	0.06	0.06	0.06	0.06
Marginal R^2 / Conditional R^2	0.247 / 0.292	0.211 / 0.259	0.183 / 0.233	0.191 / 0.236

Note: This table reports results from exposure-adjusted negative binomial count models of speeding events aggregated at the road-link level for roads with a posted speed limit of 30 mph. Models include the logarithm of the total number of GPS points observed on each link as an offset, thereby estimating the rate of speeding events per GPS observation. Network metrics (connectivity, betweenness, closeness, average shortest path, and diversion ratio) are measured at alternative network distance thresholds of 400 m, 800 m, 2 km, and 5 km, while all other covariates remain unchanged across model specifications. Results are reported as incidence rate ratios (IRR) with 95% confidence intervals and p-values.

Table S6 Exposure-adjusted negative binomial count model results for speeding events per GPS point on 40 mph road links, with network metrics measured at different network distances (400 m, 800 m, 2 km, and 5 km)

	Speeding events per GPS point (Coefficients, 95% CI, p-value, IRR, 95% CI)			
Independent variables	50m_400m	50m_800m	50m_2km	50m_5km
Traffic Calming	-0.231 (-0.362, -0.101), 0.001***, 0.794 (0.696, 0.904)	-0.221 (-0.352, -0.090), 0.001***, 0.802 (0.704, 0.914)	-0.197 (-0.329, -0.066), 0.003**, 0.821 (0.720, 0.937)	-0.237 (-0.370, -0.105), 0.000***, 0.789 (0.691, 0.900)
Choker	-0.604 (-1.057, -0.150), 0.009**, 0.547 (0.347, 0.861)	-0.541 (-0.999, -0.082), 0.021*, 0.582 (0.368, 0.921)	-0.576 (-1.038, -0.114), 0.015*, 0.562 (0.354, 0.892)	-0.592 (-1.055, -0.128), 0.012*, 0.553 (0.348, 0.880)
Island	-0.047 (-0.110, 0.016), 0.140, 0.954 (0.896, 1.016)	-0.094 (-0.157, -0.032), 0.003**, 0.910 (0.855, 0.969)	-0.112 (-0.175, -0.049), 0.000***, 0.894 (0.840, 0.952)	-0.144 (-0.208, -0.081), 0.000***, 0.866 (0.813, 0.922)
Signalised Crossing	-0.348 (-0.397, -0.299), 0.000***, 0.706 (0.672, 0.742)	-0.327 (-0.376, -0.278), 0.000***, 0.721 (0.686, 0.758)	-0.335 (-0.384, -0.286), 0.000***, 0.715 (0.681, 0.752)	-0.336 (-0.385, -0.286), 0.000***, 0.715 (0.680, 0.751)
Marked Crossing	0.056 (-0.057, 0.170), 0.333, 1.058 (0.944, 1.185)	0.043 (-0.070, 0.157), 0.458, 1.044 (0.932, 1.169)	0.052 (-0.061, 0.166), 0.367, 1.054 (0.940, 1.181)	0.034 (-0.081, 0.148), 0.563, 1.034 (0.922, 1.160)
Uncontrolled Crossing	-0.417 (-0.510, -0.325), 0.000***, 0.659 (0.601, 0.723)	-0.366 (-0.458, -0.273), 0.000***, 0.694 (0.632, 0.761)	-0.370 (-0.462, -0.278), 0.000***, 0.691 (0.630, 0.758)	-0.402 (-0.495, -0.309), 0.000***, 0.669 (0.609, 0.734)
Roundabout	-0.078 (-0.138, -0.018), 0.011*, 0.925 (0.871, 0.982)	-0.072 (-0.133, -0.012), 0.019*, 0.930 (0.876, 0.988)	-0.033 (-0.093, 0.028), 0.292, 0.968 (0.911, 1.028)	-0.103 (-0.164, -0.042), 0.001***, 0.902 (0.849, 0.959)
Mini Roundabout	-0.727 (-1.015, -0.439), 0.000***, 0.483 (0.362, 0.645)	-0.646 (-0.932, -0.359), 0.000***, 0.524 (0.394, 0.698)	-0.670 (-0.956, -0.383), 0.000***, 0.512 (0.384, 0.682)	-0.823 (-1.111, -0.534), 0.000***, 0.439 (0.329, 0.586)
Motorway Junction	0.675 (0.563, 0.787), 0.000***, 1.965 (1.756, 2.198)	0.732 (0.619, 0.844), 0.000***, 2.079 (1.858, 2.327)	0.799 (0.686, 0.912), 0.000***, 2.222 (1.985, 2.488)	0.791 (0.677, 0.905), 0.000***, 2.206 (1.968, 2.472)
Traffic Signal	-0.857 (-0.906, -0.809), 0.000***, 0.424 (0.404, 0.445)	-0.866 (-0.915, -0.818), 0.000***, 0.420 (0.401, 0.441)	-0.855 (-0.903, -0.806), 0.000***, 0.425 (0.405, 0.446)	-0.858 (-0.908, -0.809), 0.000***, 0.424 (0.404, 0.445)
Speed Camera	-0.518 (-0.635, -0.401), 0.000***, 0.596 (0.530, 0.670)	-0.582 (-0.700, -0.464), 0.000***, 0.559 (0.497, 0.629)	-0.571 (-0.690, -0.453), 0.000***, 0.565 (0.502, 0.636)	-0.614 (-0.734, -0.494), 0.000***, 0.541 (0.480, 0.610)
Link Bidirection	-0.360 (-0.401, -0.318), 0.000***, 0.698 (0.669, 0.728)	-0.454 (-0.495, -0.412), 0.000***, 0.635 (0.609, 0.662)	-0.483 (-0.529, -0.437), 0.000***, 0.617 (0.589, 0.646)	-0.483 (-0.531, -0.436), 0.000***, 0.617 (0.588, 0.647)
Link Average Width	-0.111 (-0.130, -0.092), 0.000***, 0.895 (0.878, 0.912)	-0.119 (-0.138, -0.100), 0.000***, 0.888 (0.871, 0.905)	-0.130 (-0.149, -0.111), 0.000***, 0.878 (0.862, 0.895)	-0.132 (-0.150, -0.113), 0.000***, 0.876 (0.860, 0.893)
Link Length	0.415 (0.392, 0.439), 0.000***, 1.515 (1.479, 1.552)	0.489 (0.466, 0.513), 0.000***, 1.631 (1.593, 1.670)	0.493 (0.470, 0.516), 0.000***, 1.637 (1.600, 1.676)	0.527 (0.504, 0.550), 0.000***, 1.694 (1.656, 1.733)
Link Angular Curvature	-0.425 (-0.448, -0.401), 0.000***, 0.654 (0.639, 0.669)	-0.440 (-0.462, -0.418), 0.000***, 0.644 (0.630, 0.658)	-0.453 (-0.475, -0.431), 0.000***, 0.636 (0.622, 0.650)	-0.463 (-0.484, -0.441), 0.000***, 0.630 (0.616, 0.643)
Link Degree	0.193 (0.174, 0.212), 0.000***, 1.213 (1.191, 1.236)	0.193 (0.174, 0.211), 0.000***, 1.213 (1.190, 1.235)	0.185 (0.167, 0.202), 0.000***, 1.203 (1.182, 1.224)	0.161 (0.143, 0.179), 0.000***, 1.175 (1.154, 1.196)
Connectivity	-0.371 (-0.393, -0.349), 0.000***, 0.690 (0.675, 0.705)	-0.355 (-0.377, -0.333), 0.000***, 0.701 (0.686, 0.717)	-0.183 (-0.207, -0.158), 0.000***, 0.833 (0.813, 0.854)	-0.159 (-0.187, -0.132), 0.000***, 0.853 (0.830, 0.877)
Betweenness	0.188 (0.164, 0.211), 0.000***, 1.206 (1.179, 1.235)	0.095 (0.071, 0.119), 0.000***, 1.100 (1.074, 1.126)	-0.116 (-0.140, -0.092), 0.000***, 0.891 (0.869, 0.912)	-0.029 (-0.054, -0.004), 0.023*, 0.972 (0.948, 0.996)
Closeness	0.208 (0.123, 0.294), 0.000***, 1.231 (1.130, 1.342)	0.030 (-0.005, 0.065), 0.097, 1.030 (0.995, 1.067)	0.272 (0.166, 0.377), 0.000***, 1.312 (1.181, 1.458)	0.289 (0.176, 0.401), 0.000***, 1.335 (1.193, 1.493)
Average Shortest Path	0.164 (0.144, 0.184), 0.000***, 1.178 (1.155, 1.202)	0.150 (0.132, 0.168), 0.000***, 1.162 (1.142, 1.183)	0.243 (0.225, 0.260), 0.000***, 1.274 (1.253, 1.297)	0.156 (0.139, 0.172), 0.000***, 1.168 (1.150, 1.188)
Diversion Ratio	0.043 (0.026, 0.060), 0.000***, 1.044 (1.026, 1.062)	-0.042 (-0.057, -0.027), 0.000***, 0.959 (0.945, 0.974)	-0.008 (-0.027, 0.011), 0.413, 0.992 (0.974, 1.011)	0.089 (0.069, 0.110), 0.000***, 1.093 (1.071, 1.116)
Random effect - City	0.06	0.06	0.06	0.05
ICC	0.02	0.02	0.02	0.02
Marginal R^2 / Conditional R^2	0.212 / 0.228	0.197 / 0.212	0.207 / 0.223	0.199 / 0.212

Note: This table reports results from exposure-adjusted negative binomial count models of speeding events aggregated at the road-link level for roads with a posted speed limit of 40 mph. Models include the logarithm of the total number of GPS points observed on each link as an offset, thereby estimating the rate of speeding events per GPS observation. Network metrics (connectivity, betweenness, closeness, average shortest path, and diversion ratio) are measured at alternative network distance thresholds of 400 m, 800 m, 2 km, and 5 km, while all other covariates remain unchanged across model specifications. Results are reported as incidence rate ratios (IRR) with 95% confidence intervals and p-values.

Table S7 Exposure-adjusted negative binomial count model results for speeding events per GPS point on 50 mph road links, with network metrics measured at different network distances (400 m, 800 m, 2 km, and 5 km)

	Speeding events per GPS point (Coefficients, 95% CI, p-value, IRR, 95% CI)			
Independent variables	50m_400m	50m_800m	50m_2km	50m_5km
Traffic Calming	-0.025 (-0.423, 0.372), 0.901, 0.975 (0.655, 1.451)	0.006 (-0.392, 0.405), 0.975, 1.007 (0.675, 1.500)	0.034 (-0.364, 0.432), 0.866, 1.035 (0.695, 1.541)	-0.077 (-0.474, 0.321), 0.705, 0.926 (0.622, 1.378)
Choker	-1.006 (-2.122, 0.111), 0.078, 0.366 (0.120, 1.117)	-1.133 (-2.245, -0.020), 0.046*, 0.322 (0.106, 0.980)	-1.109 (-2.227, 0.009), 0.052, 0.330 (0.108, 1.009)	-1.195 (-2.314, -0.075), 0.036*, 0.303 (0.099, 0.927)
Island	-0.722 (-0.898, -0.547), 0.000***, 0.486 (0.408, 0.579)	-0.714 (-0.891, -0.538), 0.000***, 0.490 (0.410, 0.584)	-0.706 (-0.883, -0.529), 0.000***, 0.494 (0.414, 0.589)	-0.702 (-0.880, -0.524), 0.000***, 0.496 (0.415, 0.592)
Signalised Crossing	-0.489 (-0.637, -0.341), 0.000***, 0.613 (0.529, 0.711)	-0.481 (-0.628, -0.333), 0.000***, 0.618 (0.533, 0.717)	-0.508 (-0.658, -0.358), 0.000***, 0.602 (0.518, 0.699)	-0.508 (-0.658, -0.358), 0.000***, 0.602 (0.518, 0.699)
Marked Crossing	-0.460 (-0.845, -0.074), 0.019*, 0.631 (0.429, 0.928)	-0.431 (-0.813, -0.048), 0.027*, 0.650 (0.444, 0.953)	-0.570 (-0.937, -0.203), 0.002**, 0.566 (0.392, 0.816)	-0.598 (-0.964, -0.231), 0.001**, 0.550 (0.381, 0.794)
Uncontrolled Crossing	-0.161 (-0.418, 0.095), 0.218, 0.851 (0.658, 1.100)	-0.134 (-0.390, 0.121), 0.302, 0.874 (0.677, 1.129)	-0.192 (-0.446, 0.062), 0.138, 0.825 (0.640, 1.064)	-0.174 (-0.428, 0.079), 0.178, 0.840 (0.652, 1.082)
Roundabout	0.093 (-0.027, 0.213), 0.128, 1.098 (0.974, 1.237)	0.096 (-0.024, 0.216), 0.117, 1.100 (0.976, 1.240)	0.168 (0.048, 0.289), 0.006**, 1.183 (1.049, 1.334)	0.158 (0.038, 0.279), 0.010*, 1.172 (1.038, 1.322)
Mini Roundabout	-0.042 (-0.506, 0.422), 0.860, 0.959 (0.603, 1.525)	-0.062 (-0.527, 0.404), 0.795, 0.940 (0.591, 1.497)	-0.030 (-0.496, 0.435), 0.898, 0.970 (0.609, 1.545)	0.028 (-0.438, 0.494), 0.907, 1.028 (0.645, 1.639)
Motorway Junction	0.195 (0.065, 0.324), 0.003**, 1.215 (1.067, 1.383)	0.199 (0.069, 0.328), 0.003**, 1.220 (1.071, 1.389)	0.243 (0.112, 0.374), 0.000***, 1.275 (1.119, 1.453)	0.229 (0.098, 0.360), 0.001***, 1.257 (1.103, 1.433)
Traffic Signal	-0.480 (-0.605, -0.354), 0.000***, 0.619 (0.546, 0.702)	-0.458 (-0.584, -0.332), 0.000***, 0.632 (0.558, 0.717)	-0.489 (-0.617, -0.361), 0.000***, 0.613 (0.540, 0.697)	-0.522 (-0.650, -0.395), 0.000***, 0.593 (0.522, 0.674)
Speed Camera	-0.088 (-0.371, 0.196), 0.544, 0.916 (0.690, 1.216)	-0.103 (-0.386, 0.181), 0.479, 0.902 (0.679, 1.199)	-0.049 (-0.334, 0.236), 0.735, 0.952 (0.716, 1.266)	-0.072 (-0.357, 0.213), 0.621, 0.931 (0.700, 1.238)
Link Bidirection	-1.023 (-1.129, -0.917), 0.000***, 0.359 (0.323, 0.400)	-1.144 (-1.248, -1.040), 0.000***, 0.318 (0.287, 0.353)	-1.130 (-1.240, -1.021), 0.000***, 0.323 (0.289, 0.360)	-1.192 (-1.309, -1.076), 0.000***, 0.304 (0.270, 0.341)
Link Average Width	0.072 (0.028, 0.115), 0.001**, 1.075 (1.029, 1.122)	0.065 (0.021, 0.108), 0.004**, 1.067 (1.021, 1.114)	0.071 (0.028, 0.115), 0.001**, 1.074 (1.028, 1.122)	0.069 (0.025, 0.113), 0.002**, 1.072 (1.026, 1.119)
Link Length	0.511 (0.452, 0.569), 0.000***, 1.666 (1.571, 1.767)	0.526 (0.472, 0.581), 0.000***, 1.693 (1.603, 1.787)	0.579 (0.526, 0.633), 0.000***, 1.785 (1.692, 1.883)	0.594 (0.541, 0.647), 0.000***, 1.812 (1.718, 1.910)
Link Angular Curvature	-0.569 (-0.620, -0.518), 0.000***, 0.566 (0.538, 0.596)	-0.539 (-0.591, -0.488), 0.000***, 0.583 (0.554, 0.614)	-0.569 (-0.620, -0.518), 0.000***, 0.566 (0.538, 0.595)	-0.573 (-0.624, -0.522), 0.000***, 0.564 (0.536, 0.593)
Link Degree	0.283 (0.242, 0.324), 0.000***, 1.327 (1.274, 1.383)	0.239 (0.197, 0.281), 0.000***, 1.270 (1.218, 1.325)	0.250 (0.211, 0.288), 0.000***, 1.283 (1.235, 1.334)	0.229 (0.191, 0.268), 0.000***, 1.257 (1.210, 1.307)
Connectivity	-0.217 (-0.264, -0.171), 0.000***, 0.805 (0.768, 0.843)	-0.261 (-0.308, -0.214), 0.000***, 0.770 (0.735, 0.808)	-0.082 (-0.135, -0.029), 0.003**, 0.921 (0.873, 0.972)	-0.161 (-0.224, -0.097), 0.000***, 0.852 (0.799, 0.907)
Betweenness	-0.017 (-0.069, 0.034), 0.506, 0.983 (0.934, 1.034)	0.115 (0.065, 0.165), 0.000***, 1.122 (1.067, 1.180)	-0.112 (-0.159, -0.065), 0.000***, 0.894 (0.853, 0.937)	-0.009 (-0.060, 0.042), 0.719, 0.991 (0.942, 1.042)
Closeness	0.133 (0.062, 0.203), 0.000***, 1.142 (1.064, 1.225)	0.183 (0.082, 0.283), 0.000***, 1.200 (1.085, 1.328)	0.130 (0.044, 0.216), 0.003**, 1.139 (1.045, 1.241)	0.058 (0.007, 0.109), 0.025*, 1.060 (1.007, 1.115)
Average Shortest Path	0.164 (0.117, 0.211), 0.000***, 1.179 (1.125, 1.235)	0.112 (0.070, 0.154), 0.000***, 1.119 (1.072, 1.167)	0.082 (0.046, 0.119), 0.000***, 1.086 (1.047, 1.126)	-0.003 (-0.038, 0.032), 0.871, 0.997 (0.962, 1.033)
Diversion Ratio	0.062 (0.024, 0.100), 0.001**, 1.064 (1.025, 1.105)	0.068 (0.030, 0.106), 0.001***, 1.070 (1.030, 1.112)	-0.019 (-0.061, 0.023), 0.374, 0.981 (0.941, 1.023)	0.045 (-0.001, 0.092), 0.054, 1.047 (0.999, 1.096)
Random effect - City	0.17	0.15	0.14	0.13
ICC	0.06	0.06	0.05	0.05
Marginal R^2 / Conditional R^2	0.248 / 0.295	0.248 / 0.290	0.236 / 0.276	0.233 / 0.271

Note: This table reports results from exposure-adjusted negative binomial count models of speeding events aggregated at the road-link level for roads with a posted speed limit of 50 mph. Models include the logarithm of the total number of GPS points observed on each link as an offset, thereby estimating the rate of speeding events per GPS observation. Network metrics (connectivity, betweenness, closeness, average shortest path, and diversion ratio) are measured at alternative network distance thresholds of 400 m, 800 m, 2 km, and 5 km, while all other covariates remain unchanged across model specifications. Results are reported as incidence rate ratios (IRR) with 95% confidence intervals and p-values.

Table S8 Exposure-adjusted negative binomial count model results for speeding events per GPS point on 60 mph road links, with network metrics measured at different network distances (400 m, 800 m, 2 km, and 5 km)

	Speeding events per GPS point (Coefficients, 95% CI, p-value, IRR, 95% CI)			
Independent variables	50m_400m	50m_800m	50m_2km	50m_5km
Traffic Calming	-1.530 (-2.367, -0.693), 0.000***, 0.217 (0.094, 0.500)	-1.729 (-2.617, -0.841), 0.000***, 0.177 (0.073, 0.431)	-1.551 (-2.411, -0.690), 0.000***, 0.212 (0.090, 0.502)	-1.494 (-2.338, -0.649), 0.001***, 0.225 (0.097, 0.522)
Choker	/	/	/	/
Island	-0.459 (-0.877, -0.041), 0.031*, 0.632 (0.416, 0.959)	-0.333 (-0.755, 0.090), 0.123, 0.717 (0.470, 1.094)	-0.323 (-0.751, 0.104), 0.138, 0.724 (0.472, 1.110)	-0.514 (-0.939, -0.088), 0.018*, 0.598 (0.391, 0.916)
Signalised Crossing	-0.932 (-1.348, -0.517), 0.000***, 0.394 (0.260, 0.596)	-0.861 (-1.273, -0.449), 0.000***, 0.423 (0.280, 0.638)	-0.819 (-1.229, -0.409), 0.000***, 0.441 (0.293, 0.664)	-0.902 (-1.321, -0.484), 0.000***, 0.406 (0.267, 0.617)
Marked Crossing	/	/	/	/
Uncontrolled Crossing	-0.807 (-1.661, 0.046), 0.064, 0.446 (0.190, 1.047)	-0.612 (-1.464, 0.240), 0.159, 0.542 (0.231, 1.271)	-0.442 (-1.280, 0.395), 0.301, 0.642 (0.278, 1.485)	-0.575 (-1.424, 0.273), 0.184, 0.562 (0.241, 1.314)
Roundabout	-0.373 (-0.650, -0.097), 0.008**, 0.688 (0.522, 0.908)	-0.146 (-0.421, 0.128), 0.296, 0.864 (0.657, 1.137)	-0.062 (-0.340, 0.217), 0.665, 0.940 (0.712, 1.242)	-0.189 (-0.472, 0.094), 0.191, 0.828 (0.624, 1.099)
Mini Roundabout	-0.738 (-1.417, -0.059), 0.033*, 0.478 (0.242, 0.943)	-0.553 (-1.240, 0.134), 0.115, 0.575 (0.289, 1.143)	-0.715 (-1.403, -0.027), 0.042*, 0.489 (0.246, 0.973)	-0.578 (-1.251, 0.095), 0.092, 0.561 (0.286, 1.099)
Motorway Junction	0.038 (-0.301, 0.377), 0.827, 1.038 (0.740, 1.457)	-0.023 (-0.359, 0.313), 0.895, 0.978 (0.698, 1.368)	0.216 (-0.123, 0.554), 0.212, 1.241 (0.884, 1.741)	0.211 (-0.133, 0.554), 0.230, 1.234 (0.875, 1.740)
Traffic Signal	0.375 (0.061, 0.690), 0.019*, 1.456 (1.063, 1.994)	0.524 (0.214, 0.835), 0.001***, 1.689 (1.239, 2.304)	0.597 (0.286, 0.909), 0.000***, 1.817 (1.331, 2.482)	0.629 (0.314, 0.943), 0.000***, 1.875 (1.369, 2.568)
Speed Camera	0.914 (0.037, 1.791), 0.041*, 2.494 (1.037, 5.994)	0.460 (-0.391, 1.311), 0.290, 1.584 (0.676, 3.708)	0.379 (-0.477, 1.234), 0.386, 1.460 (0.621, 3.436)	0.296 (-0.570, 1.163), 0.503, 1.345 (0.565, 3.199)
Link Bidirection	-1.939 (-2.165, -1.713), 0.000***, 0.144 (0.115, 0.180)	-2.184 (-2.407, -1.961), 0.000***, 0.113 (0.090, 0.141)	-2.267 (-2.499, -2.036), 0.000***, 0.104 (0.082, 0.131)	-2.337 (-2.581, -2.093), 0.000***, 0.097 (0.076, 0.123)
Link Average Width	0.348 (0.285, 0.411), 0.000***, 1.416 (1.329, 1.509)	0.402 (0.338, 0.465), 0.000***, 1.494 (1.402, 1.593)	0.387 (0.323, 0.451), 0.000***, 1.473 (1.381, 1.570)	0.370 (0.306, 0.434), 0.000***, 1.448 (1.359, 1.543)
Link Length	0.509 (0.425, 0.593), 0.000***, 1.663 (1.529, 1.809)	0.527 (0.458, 0.597), 0.000***, 1.694 (1.580, 1.816)	0.631 (0.563, 0.699), 0.000***, 1.880 (1.756, 2.011)	0.687 (0.620, 0.754), 0.000***, 1.988 (1.859, 2.126)
Link Angular Curvature	-1.614 (-1.740, -1.487), 0.000***, 0.199 (0.175, 0.226)	-1.506 (-1.633, -1.379), 0.000***, 0.222 (0.195, 0.252)	-1.510 (-1.636, -1.384), 0.000***, 0.221 (0.195, 0.251)	-1.564 (-1.691, -1.437), 0.000***, 0.209 (0.184, 0.238)
Link Degree	0.185 (0.132, 0.238), 0.000***, 1.203 (1.141, 1.269)	0.114 (0.059, 0.168), 0.000***, 1.121 (1.061, 1.183)	0.081 (0.030, 0.133), 0.002**, 1.085 (1.030, 1.142)	0.065 (0.016, 0.114), 0.009**, 1.068 (1.017, 1.121)
Connectivity	-0.358 (-0.434, -0.283), 0.000***, 0.699 (0.648, 0.754)	-0.432 (-0.503, -0.362), 0.000***, 0.649 (0.605, 0.696)	-0.275 (-0.337, -0.212), 0.000***, 0.760 (0.714, 0.809)	-0.345 (-0.423, -0.268), 0.000***, 0.708 (0.655, 0.765)
Betweenness	-0.025 (-0.105, 0.054), 0.529, 0.975 (0.901, 1.055)	0.066 (-0.009, 0.141), 0.085, 1.068 (0.991, 1.151)	0.018 (-0.050, 0.086), 0.608, 1.018 (0.951, 1.090)	0.109 (0.041, 0.176), 0.002**, 1.115 (1.042, 1.193)
Closeness	0.070 (-0.003, 0.144), 0.062, 1.073 (0.997, 1.155)	0.063 (0.003, 0.122), 0.039*, 1.065 (1.003, 1.130)	0.077 (0.018, 0.136), 0.011*, 1.080 (1.018, 1.146)	0.049 (-0.005, 0.103), 0.078, 1.050 (0.995, 1.109)
Average Shortest Path	0.236 (0.160, 0.312), 0.000***, 1.266 (1.173, 1.366)	0.317 (0.263, 0.371), 0.000***, 1.373 (1.300, 1.449)	0.311 (0.261, 0.361), 0.000***, 1.364 (1.298, 1.434)	0.216 (0.172, 0.259), 0.000***, 1.241 (1.187, 1.296)
Diversion Ratio	0.026 (-0.031, 0.083), 0.372, 1.026 (0.969, 1.086)	-0.040 (-0.081, 0.001), 0.056, 0.961 (0.922, 1.001)	-0.123 (-0.165, -0.081), 0.000***, 0.884 (0.848, 0.922)	-0.064 (-0.115, -0.013), 0.015*, 0.938 (0.892, 0.988)
Random effect - City	0.48	0.29	0.46	0.50
ICC	0.10	0.06	0.10	0.11
Marginal R^2 / Conditional R^2	0.395 / 0.458	0.415 / 0.452	0.391 / 0.452	0.385 / 0.451

Note: This table reports results from exposure-adjusted negative binomial count models of speeding events aggregated at the road-link level for roads with a posted speed limit of 60 mph. Models include the logarithm of the total number of GPS points observed on each link as an offset, thereby estimating the rate of speeding events per GPS observation. Network metrics (connectivity, betweenness, closeness, average shortest path, and diversion ratio) are measured at alternative network distance thresholds of 400 m, 800 m, 2 km, and 5 km, while all other covariates remain unchanged across model specifications. Results are reported as incidence rate ratios (IRR) with 95% confidence intervals and p-values.

Table S9 Exposure-adjusted negative binomial count model results for speeding events per GPS point on 70 mph road links, with network metrics measured at different network distances (400 m, 800 m, 2 km, and 5 km)

	Speeding events per GPS point (Coefficients, 95% CI, p-value, IRR, 95% CI)			
Independent variables	50m_400m	50m_800m	50m_2km	50m_5km
Traffic Calming	-0.389 (-1.025, 0.248), 0.231, 0.678 (0.359, 1.281)	-0.318 (-0.955, 0.318), 0.327, 0.727 (0.385, 1.375)	-0.117 (-0.753, 0.519), 0.719, 0.890 (0.471, 1.681)	-0.096 (-0.731, 0.539), 0.767, 0.908 (0.481, 1.715)
Choker	/	/	/	/
Island	-0.064 (-0.420, 0.292), 0.725, 0.938 (0.657, 1.340)	-0.051 (-0.408, 0.306), 0.780, 0.950 (0.665, 1.358)	-0.024 (-0.383, 0.334), 0.895, 0.976 (0.682, 1.397)	0.070 (-0.289, 0.429), 0.703, 1.072 (0.749, 1.536)
Signalised Crossing	-0.537 (-0.781, -0.293), 0.000***, 0.584 (0.458, 0.746)	-0.531 (-0.776, -0.285), 0.000***, 0.588 (0.460, 0.752)	-0.503 (-0.749, -0.256), 0.000***, 0.605 (0.473, 0.774)	-0.528 (-0.774, -0.281), 0.000***, 0.590 (0.461, 0.755)
Marked Crossing	-0.071 (-0.685, 0.543), 0.820, 0.931 (0.504, 1.721)	0.011 (-0.605, 0.627), 0.972, 1.011 (0.546, 1.871)	0.134 (-0.482, 0.751), 0.669, 1.144 (0.617, 2.119)	0.143 (-0.476, 0.761), 0.651, 1.153 (0.621, 2.140)
Uncontrolled Crossing	-0.849 (-1.241, -0.458), 0.000***, 0.428 (0.289, 0.633)	-0.814 (-1.211, -0.417), 0.000***, 0.443 (0.298, 0.659)	-0.750 (-1.146, -0.354), 0.000***, 0.473 (0.318, 0.702)	-0.741 (-1.136, -0.345), 0.000***, 0.477 (0.321, 0.708)
Roundabout	0.087 (-0.027, 0.202), 0.134, 1.091 (0.973, 1.224)	0.061 (-0.052, 0.175), 0.289, 1.063 (0.949, 1.191)	0.012 (-0.102, 0.126), 0.837, 1.012 (0.903, 1.135)	0.031 (-0.082, 0.144), 0.589, 1.032 (0.921, 1.155)
Mini Roundabout	-0.621 (-1.233, -0.009), 0.047*, 0.537 (0.291, 0.991)	-0.614 (-1.226, -0.002), 0.049*, 0.541 (0.293, 0.998)	-0.491 (-1.098, 0.115), 0.113, 0.612 (0.334, 1.122)	-0.496 (-1.099, 0.108), 0.107, 0.609 (0.333, 1.114)
Motorway Junction	-0.111 (-0.212, -0.009), 0.033*, 0.895 (0.809, 0.991)	-0.101 (-0.202, -0.001), 0.049*, 0.904 (0.817, 0.999)	-0.095 (-0.195, 0.006), 0.065, 0.910 (0.823, 1.006)	-0.095 (-0.196, 0.005), 0.063, 0.909 (0.822, 1.005)
Traffic Signal	-0.220 (-0.344, -0.096), 0.001***, 0.803 (0.709, 0.909)	-0.199 (-0.324, -0.074), 0.002**, 0.820 (0.723, 0.929)	-0.173 (-0.298, -0.048), 0.007**, 0.841 (0.743, 0.953)	-0.144 (-0.268, -0.019), 0.024*, 0.866 (0.765, 0.981)
Speed Camera	-0.361 (-0.728, 0.007), 0.054, 0.697 (0.483, 1.007)	-0.373 (-0.743, -0.004), 0.048*, 0.688 (0.476, 0.996)	-0.311 (-0.680, 0.058), 0.099, 0.733 (0.507, 1.060)	-0.303 (-0.673, 0.067), 0.108, 0.739 (0.510, 1.069)
Link Bidirection	/	/	/	/
Link Average Width	0.349 (0.299, 0.399), 0.000***, 1.418 (1.348, 1.491)	0.351 (0.301, 0.401), 0.000***, 1.421 (1.351, 1.494)	0.360 (0.309, 0.411), 0.000***, 1.433 (1.363, 1.508)	0.369 (0.318, 0.420), 0.000***, 1.446 (1.374, 1.522)
Link Length	0.606 (0.522, 0.689), 0.000***, 1.832 (1.686, 1.992)	0.431 (0.373, 0.489), 0.000***, 1.538 (1.452, 1.630)	0.419 (0.358, 0.481), 0.000***, 1.521 (1.430, 1.617)	0.438 (0.380, 0.496), 0.000***, 1.549 (1.462, 1.642)
Link Angular Curvature	-0.438 (-0.489, -0.388), 0.000***, 0.645 (0.613, 0.678)	-0.422 (-0.472, -0.372), 0.000***, 0.656 (0.624, 0.690)	-0.415 (-0.465, -0.365), 0.000***, 0.660 (0.628, 0.694)	-0.413 (-0.463, -0.363), 0.000***, 0.662 (0.629, 0.696)
Link Degree	0.230 (0.188, 0.273), 0.000***, 1.259 (1.207, 1.314)	0.247 (0.201, 0.293), 0.000***, 1.280 (1.222, 1.341)	0.263 (0.220, 0.306), 0.000***, 1.301 (1.246, 1.358)	0.218 (0.178, 0.258), 0.000***, 1.243 (1.195, 1.294)
Connectivity	-0.083 (-0.133, -0.032), 0.001**, 0.921 (0.875, 0.968)	-0.089 (-0.138, -0.040), 0.000***, 0.915 (0.871, 0.961)	-0.065 (-0.119, -0.011), 0.017*, 0.937 (0.888, 0.989)	-0.110 (-0.175, -0.046), 0.001***, 0.896 (0.839, 0.955)
Betweenness	-0.196 (-0.252, -0.141), 0.000***, 0.822 (0.777, 0.869)	-0.079 (-0.133, -0.025), 0.004**, 0.924 (0.875, 0.975)	-0.141 (-0.197, -0.084), 0.000***, 0.869 (0.821, 0.919)	-0.138 (-0.198, -0.078), 0.000***, 0.871 (0.820, 0.925)
Closeness	0.023 (-0.019, 0.065), 0.279, 1.023 (0.981, 1.067)	-0.059 (-0.130, 0.011), 0.100, 0.942 (0.878, 1.012)	-0.039 (-0.102, 0.025), 0.233, 0.962 (0.903, 1.025)	-0.031 (-0.097, 0.036), 0.362, 0.970 (0.907, 1.036)
Average Shortest Path	-0.001 (-0.072, 0.069), 0.969, 0.999 (0.931, 1.071)	0.004 (-0.042, 0.050), 0.871, 1.004 (0.959, 1.051)	-0.009 (-0.050, 0.033), 0.679, 0.991 (0.951, 1.033)	0.028 (-0.014, 0.070), 0.197, 1.028 (0.986, 1.072)
Diversion Ratio	0.110 (-0.031, 0.252), 0.127, 1.117 (0.969, 1.286)	0.072 (0.022, 0.122), 0.005**, 1.075 (1.022, 1.130)	-0.012 (-0.056, 0.033), 0.607, 0.988 (0.946, 1.033)	-0.041 (-0.081, -0.002), 0.041*, 0.960 (0.923, 0.998)
Random effect - City	0.31	0.30	0.29	0.30
ICC	0.08	0.08	0.07	0.08
Marginal R^2 / Conditional R^2	0.113 / 0.182	0.111 / 0.180	0.115 / 0.181	0.122 / 0.189

Note: This table reports results from exposure-adjusted negative binomial count models of speeding events aggregated at the road-link level for roads with a posted speed limit of 70 mph. Models include the logarithm of the total number of GPS points observed on each link as an offset, thereby estimating the rate of speeding events per GPS observation. Network metrics (connectivity, betweenness, closeness, average shortest path, and diversion ratio) are measured at alternative network distance thresholds of 400 m, 800 m, 2 km, and 5 km, while all other covariates remain unchanged across model specifications. Results are reported as incidence rate ratios (IRR) with 95% confidence intervals and p-values.

Raw negative binomial count model

Table S10 Negative binomial count model results for speeding events per road link on all road links, with network metrics measured at different network distances (400 m, 800 m, 2 km, and 5 km)

	Speeding events per road link (Coefficients, 95% CI, p-value, IRR, 95% CI)			
Independent variables	50m_400m	50m_800m	50m_2km	50m_5km
Traffic Calming	-0.401 (-0.418, -0.385), 0.000***, 0.669 (0.659, 0.680)	-0.408 (-0.424, -0.392), 0.000***, 0.665 (0.654, 0.676)	-0.408 (-0.424, -0.392), 0.000***, 0.665 (0.655, 0.676)	-0.377 (-0.393, -0.361), 0.000***, 0.686 (0.675, 0.697)
Choker	-0.176 (-0.234, -0.118), 0.000***, 0.839 (0.792, 0.889)	-0.193 (-0.251, -0.134), 0.000***, 0.825 (0.778, 0.874)	-0.190 (-0.247, -0.133), 0.000***, 0.827 (0.781, 0.875)	-0.177 (-0.233, -0.122), 0.000***, 0.838 (0.792, 0.886)
Island	0.620 (0.597, 0.642), 0.000***, 1.858 (1.817, 1.900)	0.566 (0.544, 0.589), 0.000***, 1.762 (1.723, 1.801)	0.540 (0.519, 0.562), 0.000***, 1.717 (1.680, 1.754)	0.465 (0.443, 0.486), 0.000***, 1.592 (1.558, 1.626)
Signalised Crossing	0.281 (0.260, 0.301), 0.000***, 1.324 (1.297, 1.351)	0.303 (0.283, 0.324), 0.000***, 1.354 (1.327, 1.382)	0.246 (0.226, 0.266), 0.000***, 1.279 (1.253, 1.305)	0.155 (0.135, 0.175), 0.000***, 1.168 (1.144, 1.192)
Marked Crossing	0.158 (0.124, 0.191), 0.000***, 1.171 (1.132, 1.211)	0.198 (0.164, 0.232), 0.000***, 1.219 (1.179, 1.261)	0.142 (0.109, 0.175), 0.000***, 1.152 (1.115, 1.191)	0.107 (0.075, 0.139), 0.000***, 1.113 (1.077, 1.150)
Uncontrolled Crossing	0.292 (0.267, 0.317), 0.000***, 1.339 (1.306, 1.373)	0.297 (0.272, 0.322), 0.000***, 1.346 (1.313, 1.380)	0.217 (0.193, 0.242), 0.000***, 1.243 (1.213, 1.273)	0.197 (0.173, 0.221), 0.000***, 1.218 (1.189, 1.247)
Roundabout	0.500 (0.468, 0.532), 0.000***, 1.649 (1.597, 1.702)	0.503 (0.471, 0.534), 0.000***, 1.653 (1.602, 1.706)	0.366 (0.335, 0.397), 0.000***, 1.442 (1.398, 1.487)	0.190 (0.160, 0.220), 0.000***, 1.209 (1.174, 1.246)
Mini Roundabout	-0.103 (-0.137, -0.069), 0.000***, 0.902 (0.872, 0.934)	-0.126 (-0.160, -0.092), 0.000***, 0.882 (0.852, 0.912)	-0.252 (-0.285, -0.219), 0.000***, 0.777 (0.752, 0.803)	-0.268 (-0.301, -0.235), 0.000***, 0.765 (0.740, 0.790)
Motorway Junction	0.424 (0.341, 0.506), 0.000***, 1.527 (1.406, 1.659)	0.453 (0.370, 0.536), 0.000***, 1.573 (1.448, 1.709)	0.566 (0.486, 0.647), 0.000***, 1.762 (1.626, 1.910)	0.426 (0.347, 0.505), 0.000***, 1.532 (1.415, 1.658)
Traffic Signal	-0.178 (-0.197, -0.159), 0.000***, 0.837 (0.821, 0.853)	-0.188 (-0.208, -0.169), 0.000***, 0.828 (0.813, 0.845)	-0.191 (-0.210, -0.171), 0.000***, 0.826 (0.811, 0.842)	-0.279 (-0.299, -0.260), 0.000***, 0.756 (0.742, 0.771)
Speed Camera	0.222 (0.143, 0.300), 0.000***, 1.248 (1.154, 1.350)	0.356 (0.276, 0.435), 0.000***, 1.427 (1.318, 1.545)	0.371 (0.294, 0.448), 0.000***, 1.449 (1.342, 1.565)	0.305 (0.229, 0.380), 0.000***, 1.356 (1.258, 1.462)
Link Bidirection	-0.787 (-0.804, -0.770), 0.000***, 0.455 (0.447, 0.463)	-0.711 (-0.728, -0.694), 0.000***, 0.491 (0.483, 0.499)	-1.114 (-1.131, -1.097), 0.000***, 0.328 (0.323, 0.334)	-1.054 (-1.071, -1.038), 0.000***, 0.348 (0.343, 0.354)
Link Average Width	0.820 (0.812, 0.828), 0.000***, 2.271 (2.253, 2.289)	0.810 (0.802, 0.818), 0.000***, 2.248 (2.230, 2.266)	0.757 (0.750, 0.765), 0.000***, 2.132 (2.117, 2.148)	0.684 (0.677, 0.691), 0.000***, 1.982 (1.967, 1.997)
Link Length	1.056 (1.045, 1.066), 0.000***, 2.874 (2.844, 2.904)	1.252 (1.242, 1.262), 0.000***, 3.498 (3.464, 3.533)	1.397 (1.388, 1.406), 0.000***, 4.043 (4.006, 4.081)	1.405 (1.396, 1.414), 0.000***, 4.074 (4.037, 4.111)
Link Angular Curvature	-0.391 (-0.398, -0.385), 0.000***, 0.676 (0.672, 0.681)	-0.488 (-0.494, -0.482), 0.000***, 0.614 (0.610, 0.618)	-0.508 (-0.514, -0.502), 0.000***, 0.602 (0.598, 0.605)	-0.510 (-0.516, -0.504), 0.000***, 0.600 (0.597, 0.604)
Link Degree	0.034 (0.028, 0.040), 0.000***, 1.035 (1.029, 1.041)	-0.056 (-0.062, -0.051), 0.000***, 0.945 (0.940, 0.951)	-0.112 (-0.117, -0.107), 0.000***, 0.894 (0.889, 0.899)	-0.090 (-0.095, -0.085), 0.000***, 0.914 (0.909, 0.918)
Connectivity	-0.086 (-0.096, -0.076), 0.000***, 0.917 (0.908, 0.927)	-0.514 (-0.523, -0.505), 0.000***, 0.598 (0.593, 0.603)	-0.817 (-0.824, -0.810), 0.000***, 0.442 (0.439, 0.445)	-0.664 (-0.670, -0.657), 0.000***, 0.515 (0.512, 0.518)
Betweenness	0.133 (0.122, 0.144), 0.000***, 1.142 (1.129, 1.155)	0.531 (0.521, 0.542), 0.000***, 1.701 (1.683, 1.720)	1.074 (1.065, 1.083), 0.000***, 2.927 (2.900, 2.955)	1.142 (1.134, 1.151), 0.000***, 3.134 (3.109, 3.160)
Closeness	2.375 (2.317, 2.433), 0.000***, 10.752 (10.150, 11.390)	0.929 (0.859, 0.998), 0.000***, 2.531 (2.361, 2.713)	0.395 (0.358, 0.432), 0.000***, 1.485 (1.431, 1.541)	0.466 (0.422, 0.509), 0.000***, 1.593 (1.526, 1.664)
Average Shortest Path	0.764 (0.755, 0.773), 0.000***, 2.147 (2.128, 2.165)	0.516 (0.509, 0.522), 0.000***, 1.675 (1.663, 1.686)	0.337 (0.331, 0.342), 0.000***, 1.401 (1.393, 1.408)	0.257 (0.252, 0.262), 0.000***, 1.293 (1.286, 1.299)
Diversion Ratio	0.007 (-0.009, 0.022), 0.388, 1.007 (0.991, 1.022)	-0.040 (-0.045, -0.035), 0.000***, 0.960 (0.956, 0.965)	-0.246 (-0.251, -0.242), 0.000***, 0.782 (0.778, 0.785)	-0.173 (-0.178, -0.168), 0.000***, 0.841 (0.837, 0.845)
Speed Limit 20 mph	3.029 (2.961, 3.096), 0.000***, 20.667 (19.313, 22.115)	3.088 (3.020, 3.156), 0.000***, 21.939 (20.500, 23.478)	3.098 (3.031, 3.165), 0.000***, 22.155 (20.719, 23.691)	2.975 (2.910, 3.041), 0.000***, 19.599 (18.351, 20.931)
Speed Limit 30 mph	2.276 (2.209, 2.342), 0.000***, 9.734 (9.109, 10.402)	2.321 (2.254, 2.387), 0.000***, 10.183 (9.529, 10.882)	2.173 (2.108, 2.239), 0.000***, 8.788 (8.231, 9.383)	1.819 (1.754, 1.883), 0.000***, 6.163 (5.780, 6.572)
Speed Limit 40 mph	1.933 (1.864, 2.002), 0.000***, 6.909 (6.450, 7.401)	2.021 (1.953, 2.090), 0.000***, 7.549 (7.048, 8.085)	1.896 (1.829, 1.964), 0.000***, 6.662 (6.227, 7.127)	1.594 (1.528, 1.661), 0.000***, 4.926 (4.610, 5.263)
Speed Limit 50 mph	2.119 (2.037, 2.200), 0.000***, 8.320 (7.666, 9.029)	2.260 (2.178, 2.342), 0.000***, 9.584 (8.833, 10.399)	2.194 (2.115, 2.274), 0.000***, 8.974 (8.286, 9.720)	2.042 (1.964, 2.120), 0.000***, 7.705 (7.125, 8.332)
Speed Limit 60 mph	-1.210 (-1.290, -1.131), 0.000***, 0.298 (0.275, 0.323)	-0.966 (-1.045, -0.887), 0.000***, 0.381 (0.352, 0.412)	-1.171 (-1.249, -1.094), 0.000***, 0.310 (0.287, 0.335)	-1.201 (-1.277, -1.125), 0.000***, 0.301 (0.279, 0.325)
Random effect - City	0.17	0.17	0.16	0.15
ICC	0.09	0.08	0.08	0.08
Marginal R^2 / Conditional R^2	0.801 / 0.818	0.635 / 0.665	0.632 / 0.662	0.655 / 0.682

Note: This table reports results from negative binomial count models of observed speeding events aggregated at the road-link level. Models are estimated for all road links and stratified by posted speed limit. The dependent variable is the total number of speeding events per link. Network metrics (connectivity, betweenness, closeness, average shortest path, and diversion ratio) are measured at alternative network distance thresholds of 400 m, 800 m, 2 km, and 5 km, while all other covariates remain unchanged across model specifications. No adjustment is made for traffic exposure or GPS observation intensity. Results are reported as incidence rate ratios (IRR) with 95% confidence intervals and p-values. The reference category for speed-limit fixed effects is 70 mph.

Table S11 Negative binomial count model results for speeding events per road link on 20 mph roads, with network metrics measured at different network distances (400 m, 800 m, 2 km, and 5 km)

	Speeding events per road link (Coefficients, 95% CI, p-value, IRR, 95% CI)			
Independent variables	50m_400m	50m_800m	50m_2km	50m_5km
Traffic Calming	-0.379 (-0.397, -0.361), 0.000***, 0.684 (0.672, 0.697)	-0.399 (-0.417, -0.381), 0.000***, 0.671 (0.659, 0.683)	-0.416 (-0.433, -0.399), 0.000***, 0.660 (0.648, 0.671)	-0.342 (-0.359, -0.325), 0.000***, 0.710 (0.698, 0.722)
Choker	-0.013 (-0.077, 0.051), 0.684, 0.987 (0.926, 1.052)	-0.047 (-0.110, 0.017), 0.152, 0.954 (0.896, 1.017)	-0.087 (-0.148, -0.027), 0.005**, 0.916 (0.863, 0.973)	-0.050 (-0.109, 0.009), 0.099, 0.951 (0.897, 1.009)
Island	0.840 (0.807, 0.873), 0.000***, 2.317 (2.242, 2.394)	0.778 (0.745, 0.810), 0.000***, 2.176 (2.107, 2.248)	0.666 (0.636, 0.697), 0.000***, 1.947 (1.888, 2.008)	0.610 (0.579, 0.640), 0.000***, 1.840 (1.785, 1.896)
Signalised Crossing	0.469 (0.434, 0.505), 0.000***, 1.599 (1.543, 1.657)	0.496 (0.460, 0.531), 0.000***, 1.641 (1.584, 1.701)	0.371 (0.337, 0.406), 0.000***, 1.450 (1.400, 1.501)	0.220 (0.184, 0.255), 0.000***, 1.246 (1.202, 1.291)
Marked Crossing	0.431 (0.382, 0.479), 0.000***, 1.538 (1.466, 1.615)	0.459 (0.410, 0.507), 0.000***, 1.582 (1.507, 1.660)	0.360 (0.314, 0.406), 0.000***, 1.433 (1.369, 1.501)	0.364 (0.319, 0.409), 0.000***, 1.439 (1.376, 1.506)
Uncontrolled Crossing	0.550 (0.519, 0.582), 0.000***, 1.734 (1.681, 1.789)	0.550 (0.519, 0.581), 0.000***, 1.733 (1.680, 1.788)	0.425 (0.396, 0.455), 0.000***, 1.530 (1.486, 1.576)	0.370 (0.341, 0.399), 0.000***, 1.448 (1.406, 1.491)
Roundabout	1.086 (1.020, 1.152), 0.000***, 2.962 (2.772, 3.166)	1.072 (1.006, 1.138), 0.000***, 2.922 (2.735, 3.122)	0.755 (0.693, 0.817), 0.000***, 2.127 (1.999, 2.263)	0.656 (0.596, 0.717), 0.000***, 1.928 (1.814, 2.048)
Mini Roundabout	0.414 (0.368, 0.460), 0.000***, 1.513 (1.444, 1.584)	0.367 (0.321, 0.413), 0.000***, 1.443 (1.378, 1.511)	0.160 (0.117, 0.204), 0.000***, 1.174 (1.124, 1.226)	0.152 (0.109, 0.195), 0.000***, 1.164 (1.115, 1.215)
Motorway Junction	0.748 (0.293, 1.202), 0.001**, 2.112 (1.341, 3.326)	0.699 (0.245, 1.153), 0.003**, 2.011 (1.278, 3.166)	0.569 (0.142, 0.997), 0.009**, 1.767 (1.152, 2.710)	0.841 (0.418, 1.263), 0.000***, 2.318 (1.520, 3.536)
Traffic Signal	0.243 (0.209, 0.277), 0.000***, 1.275 (1.233, 1.320)	0.213 (0.179, 0.247), 0.000***, 1.237 (1.196, 1.280)	0.190 (0.156, 0.223), 0.000***, 1.209 (1.169, 1.250)	0.089 (0.055, 0.123), 0.000***, 1.093 (1.056, 1.131)
Speed Camera	0.056 (-0.127, 0.239), 0.548, 1.058 (0.881, 1.270)	-0.005 (-0.187, 0.178), 0.958, 0.995 (0.829, 1.194)	0.097 (-0.075, 0.269), 0.267, 1.102 (0.928, 1.309)	-0.205 (-0.376, -0.034), 0.019*, 0.815 (0.687, 0.966)
Link Bidirection	-0.420 (-0.447, -0.392), 0.000***, 0.657 (0.640, 0.675)	-0.334 (-0.361, -0.307), 0.000***, 0.716 (0.697, 0.736)	-0.639 (-0.666, -0.612), 0.000***, 0.528 (0.514, 0.542)	-0.476 (-0.501, -0.451), 0.000***, 0.621 (0.606, 0.637)
Link Average Width	0.770 (0.759, 0.780), 0.000***, 2.159 (2.137, 2.181)	0.762 (0.751, 0.772), 0.000***, 2.142 (2.120, 2.164)	0.658 (0.648, 0.667), 0.000***, 1.930 (1.913, 1.948)	0.602 (0.593, 0.612), 0.000***, 1.826 (1.810, 1.843)
Link Length	0.638 (0.625, 0.650), 0.000***, 1.892 (1.869, 1.915)	0.811 (0.799, 0.822), 0.000***, 2.250 (2.223, 2.276)	0.909 (0.898, 0.919), 0.000***, 2.482 (2.456, 2.508)	0.886 (0.876, 0.896), 0.000***, 2.425 (2.401, 2.450)
Link Angular Curvature	-0.105 (-0.115, -0.095), 0.000***, 0.901 (0.892, 0.910)	-0.208 (-0.218, -0.199), 0.000***, 0.812 (0.804, 0.820)	-0.237 (-0.246, -0.229), 0.000***, 0.789 (0.782, 0.796)	-0.259 (-0.267, -0.251), 0.000***, 0.772 (0.766, 0.778)
Link Degree	-0.079 (-0.089, -0.070), 0.000***, 0.924 (0.915, 0.933)	-0.174 (-0.183, -0.165), 0.000***, 0.840 (0.833, 0.848)	-0.174 (-0.182, -0.167), 0.000***, 0.840 (0.833, 0.846)	-0.117 (-0.124, -0.109), 0.000***, 0.890 (0.883, 0.897)
Connectivity	0.006 (-0.009, 0.022), 0.419, 1.006 (0.991, 1.022)	-0.443 (-0.458, -0.429), 0.000***, 0.642 (0.633, 0.651)	-0.771 (-0.780, -0.761), 0.000***, 0.463 (0.458, 0.467)	-0.499 (-0.508, -0.490), 0.000***, 0.607 (0.602, 0.612)
Betweenness	0.241 (0.223, 0.259), 0.000***, 1.272 (1.250, 1.295)	0.775 (0.757, 0.793), 0.000***, 2.171 (2.132, 2.209)	1.314 (1.301, 1.327), 0.000***, 3.721 (3.674, 3.768)	1.210 (1.199, 1.222), 0.000***, 3.355 (3.317, 3.393)
Closeness	0.363 (0.350, 0.377), 0.000***, 1.438 (1.418, 1.458)	0.148 (0.134, 0.162), 0.000***, 1.160 (1.143, 1.176)	0.004 (0.000, 0.007), 0.033*, 1.004 (1.000, 1.007)	0.009 (0.004, 0.013), 0.000***, 1.009 (1.004, 1.013)
Average Shortest Path	0.687 (0.674, 0.699), 0.000***, 1.987 (1.963, 2.011)	0.419 (0.408, 0.429), 0.000***, 1.520 (1.504, 1.536)	0.167 (0.159, 0.174), 0.000***, 1.181 (1.172, 1.190)	0.163 (0.155, 0.170), 0.000***, 1.177 (1.168, 1.185)
Diversion Ratio	-0.007 (-0.015, 0.001), 0.077, 0.993 (0.985, 1.001)	-0.161 (-0.172, -0.150), 0.000***, 0.851 (0.842, 0.861)	-0.374 (-0.386, -0.362), 0.000***, 0.688 (0.680, 0.696)	-0.112 (-0.120, -0.104), 0.000***, 0.894 (0.887, 0.901)
Random effect - City	0.22	0.22	0.23	0.21
ICC	0.12	0.11	0.13	0.12
Marginal R^2 / Conditional R^2	0.485 / 0.545	0.496 / 0.553	0.596 / 0.647	0.592 / 0.641

Note: This table reports results from negative binomial count models of observed speeding events aggregated at the road-link level for roads with a posted speed limit of 20 mph. The dependent variable is the total number of speeding events per link. Network metrics (connectivity, betweenness, closeness, average shortest path, and diversion ratio) are measured at alternative network distance thresholds of 400 m, 800 m, 2 km, and 5 km, while all other covariates remain unchanged across model specifications. No adjustment is made for traffic exposure or GPS observation intensity. Results are reported as incidence rate ratios (IRR) with 95% confidence intervals and p-values.

Table S12 Negative binomial count model results for speeding events per road link on 30 mph roads, with network metrics measured at different network distances (400 m, 800 m, 2 km, and 5 km)

	Speeding events per road link (Coefficients, 95% CI, p-value, IRR, 95% CI)			
Independent variables	50m_400m	50m_800m	50m_2km	50m_5km
Traffic Calming	-0.492 (-0.523, -0.461), 0.000***, 0.611 (0.593, 0.631)	-0.468 (-0.500, -0.437), 0.000***, 0.626 (0.607, 0.646)	-0.485 (-0.516, -0.454), 0.000***, 0.616 (0.597, 0.635)	-0.449 (-0.479, -0.419), 0.000***, 0.638 (0.619, 0.658)
Choker	-0.471 (-0.582, -0.359), 0.000***, 0.625 (0.559, 0.698)	-0.452 (-0.563, -0.340), 0.000***, 0.637 (0.569, 0.712)	-0.426 (-0.536, -0.316), 0.000***, 0.653 (0.585, 0.729)	-0.448 (-0.556, -0.340), 0.000***, 0.639 (0.574, 0.712)
Island	0.466 (0.434, 0.497), 0.000***, 1.593 (1.543, 1.645)	0.393 (0.361, 0.425), 0.000***, 1.482 (1.435, 1.530)	0.386 (0.355, 0.417), 0.000***, 1.471 (1.426, 1.518)	0.313 (0.283, 0.343), 0.000***, 1.368 (1.327, 1.410)
Signalised Crossing	0.217 (0.189, 0.245), 0.000***, 1.242 (1.208, 1.277)	0.239 (0.211, 0.266), 0.000***, 1.270 (1.235, 1.305)	0.215 (0.187, 0.242), 0.000***, 1.239 (1.206, 1.274)	0.141 (0.113, 0.168), 0.000***, 1.151 (1.120, 1.183)
Marked Crossing	-0.016 (-0.064, 0.032), 0.502, 0.984 (0.938, 1.032)	0.063 (0.015, 0.111), 0.010*, 1.065 (1.015, 1.118)	0.038 (-0.009, 0.086), 0.113, 1.039 (0.991, 1.089)	-0.095 (-0.142, -0.049), 0.000***, 0.909 (0.868, 0.952)
Uncontrolled Crossing	-0.152 (-0.191, -0.113), 0.000***, 0.859 (0.826, 0.893)	-0.153 (-0.192, -0.114), 0.000***, 0.858 (0.825, 0.892)	-0.220 (-0.258, -0.181), 0.000***, 0.803 (0.773, 0.834)	-0.181 (-0.218, -0.143), 0.000***, 0.835 (0.804, 0.867)
Roundabout	0.041 (-0.002, 0.085), 0.062, 1.042 (0.998, 1.088)	0.088 (0.045, 0.132), 0.000***, 1.092 (1.046, 1.141)	-0.016 (-0.059, 0.026), 0.444, 0.984 (0.943, 1.026)	-0.119 (-0.160, -0.078), 0.000***, 0.888 (0.852, 0.925)
Mini Roundabout	-0.909 (-0.958, -0.860), 0.000***, 0.403 (0.384, 0.423)	-0.931 (-0.980, -0.882), 0.000***, 0.394 (0.375, 0.414)	-1.009 (-1.058, -0.961), 0.000***, 0.364 (0.347, 0.383)	-1.002 (-1.050, -0.955), 0.000***, 0.367 (0.350, 0.385)
Motorway Junction	0.671 (0.492, 0.849), 0.000***, 1.955 (1.636, 2.337)	0.719 (0.536, 0.902), 0.000***, 2.052 (1.709, 2.464)	1.002 (0.821, 1.183), 0.000***, 2.725 (2.274, 3.265)	0.939 (0.765, 1.113), 0.000***, 2.558 (2.150, 3.044)
Traffic Signal	-0.473 (-0.499, -0.447), 0.000***, 0.623 (0.607, 0.639)	-0.468 (-0.495, -0.442), 0.000***, 0.626 (0.610, 0.643)	-0.478 (-0.504, -0.452), 0.000***, 0.620 (0.604, 0.636)	-0.569 (-0.595, -0.543), 0.000***, 0.566 (0.552, 0.581)
Speed Camera	0.299 (0.193, 0.404), 0.000***, 1.348 (1.213, 1.498)	0.282 (0.176, 0.388), 0.000***, 1.326 (1.193, 1.474)	0.289 (0.185, 0.392), 0.000***, 1.335 (1.203, 1.480)	0.276 (0.174, 0.377), 0.000***, 1.317 (1.190, 1.458)
Link Bidirection	-1.079 (-1.104, -1.053), 0.000***, 0.340 (0.332, 0.349)	-1.002 (-1.027, -0.977), 0.000***, 0.367 (0.358, 0.376)	-1.502 (-1.528, -1.476), 0.000***, 0.223 (0.217, 0.229)	-1.493 (-1.517, -1.468), 0.000***, 0.225 (0.219, 0.230)
Link Average Width	0.831 (0.819, 0.842), 0.000***, 2.295 (2.268, 2.321)	0.813 (0.801, 0.824), 0.000***, 2.254 (2.228, 2.281)	0.782 (0.771, 0.793), 0.000***, 2.186 (2.161, 2.210)	0.714 (0.703, 0.725), 0.000***, 2.042 (2.020, 2.064)
Link Length	1.111 (1.098, 1.124), 0.000***, 3.039 (2.999, 3.079)	1.272 (1.259, 1.284), 0.000***, 3.568 (3.523, 3.613)	1.386 (1.374, 1.398), 0.000***, 3.998 (3.951, 4.046)	1.409 (1.397, 1.420), 0.000***, 4.091 (4.044, 4.138)
Link Angular Curvature	-0.747 (-0.758, -0.735), 0.000***, 0.474 (0.468, 0.479)	-0.867 (-0.878, -0.856), 0.000***, 0.420 (0.415, 0.425)	-0.911 (-0.922, -0.900), 0.000***, 0.402 (0.398, 0.407)	-0.862 (-0.873, -0.852), 0.000***, 0.422 (0.418, 0.427)
Link Degree	0.058 (0.049, 0.067), 0.000***, 1.060 (1.050, 1.069)	-0.044 (-0.052, -0.036), 0.000***, 0.957 (0.949, 0.965)	-0.133 (-0.140, -0.125), 0.000***, 0.876 (0.869, 0.883)	-0.127 (-0.135, -0.120), 0.000***, 0.881 (0.874, 0.887)
Connectivity	-0.032 (-0.045, -0.018), 0.000***, 0.969 (0.956, 0.982)	-0.437 (-0.451, -0.424), 0.000***, 0.646 (0.637, 0.655)	-0.808 (-0.819, -0.798), 0.000***, 0.446 (0.441, 0.450)	-0.761 (-0.770, -0.753), 0.000***, 0.467 (0.463, 0.471)
Betweenness	0.010 (-0.005, 0.025), 0.184, 1.010 (0.995, 1.026)	0.329 (0.314, 0.345), 0.000***, 1.390 (1.368, 1.412)	0.896 (0.882, 0.910), 0.000***, 2.450 (2.415, 2.485)	1.118 (1.106, 1.130), 0.000***, 3.058 (3.021, 3.095)
Closeness	1.093 (1.061, 1.124), 0.000***, 2.983 (2.890, 3.079)	0.718 (0.645, 0.792), 0.000***, 2.051 (1.906, 2.207)	0.294 (0.247, 0.340), 0.000***, 1.342 (1.281, 1.405)	0.401 (0.341, 0.460), 0.000***, 1.493 (1.407, 1.584)
Average Shortest Path	0.789 (0.777, 0.801), 0.000***, 2.201 (2.175, 2.227)	0.580 (0.570, 0.590), 0.000***, 1.786 (1.769, 1.804)	0.408 (0.400, 0.416), 0.000***, 1.503 (1.492, 1.515)	0.312 (0.305, 0.319), 0.000***, 1.366 (1.356, 1.376)
Diversion Ratio	0.004 (0.000, 0.008), 0.026*, 1.004 (1.000, 1.008)	-0.006 (-0.007, -0.004), 0.000***, 0.994 (0.993, 0.996)	-0.210 (-0.217, -0.204), 0.000***, 0.810 (0.805, 0.815)	-0.221 (-0.228, -0.214), 0.000***, 0.802 (0.796, 0.807)
Random effect - City	0.29	0.28	0.28	0.27
ICC	0.13	0.13	0.13	0.13
Marginal R^2 / Conditional R^2	0.626 / 0.676	0.586 / 0.638	0.589 / 0.642	0.624 / 0.672

Note: This table reports results from negative binomial count models of observed speeding events aggregated at the road-link level for roads with a posted speed limit of 30 mph. The dependent variable is the total number of speeding events per link. Network metrics (connectivity, betweenness, closeness, average shortest path, and diversion ratio) are measured at alternative network distance thresholds of 400 m, 800 m, 2 km, and 5 km, while all other covariates remain unchanged across model specifications. No adjustment is made for traffic exposure or GPS observation intensity. Results are reported as incidence rate ratios (IRR) with 95% confidence intervals and p-values.

Table S13 Negative binomial count model results for speeding events per road link on 40 mph roads, with network metrics measured at different network distances (400 m, 800 m, 2 km, and 5 km)

	Speeding events per road link (Coefficients, 95% CI, p-value, IRR, 95% CI)			
Independent variables	50m_400m	50m_800m	50m_2km	50m_5km
Traffic Calming	-0.437 (-0.585, -0.290), 0.000***, 0.646 (0.557, 0.748)	-0.423 (-0.571, -0.275), 0.000***, 0.655 (0.565, 0.760)	-0.374 (-0.522, -0.226), 0.000***, 0.688 (0.594, 0.798)	-0.406 (-0.554, -0.258), 0.000***, 0.666 (0.574, 0.773)
Choker	-1.236 (-1.752, -0.720), 0.000***, 0.291 (0.173, 0.487)	-1.247 (-1.769, -0.726), 0.000***, 0.287 (0.171, 0.484)	-1.281 (-1.800, -0.761), 0.000***, 0.278 (0.165, 0.467)	-1.381 (-1.900, -0.861), 0.000***, 0.251 (0.150, 0.423)
Island	0.014 (-0.057, 0.086), 0.697, 1.014 (0.944, 1.089)	-0.029 (-0.100, 0.042), 0.426, 0.971 (0.904, 1.043)	-0.038 (-0.109, 0.034), 0.301, 0.963 (0.897, 1.034)	-0.067 (-0.139, 0.005), 0.067, 0.935 (0.870, 1.005)
Signalised Crossing	-0.378 (-0.433, -0.323), 0.000***, 0.685 (0.648, 0.724)	-0.370 (-0.426, -0.315), 0.000***, 0.691 (0.653, 0.730)	-0.364 (-0.420, -0.309), 0.000***, 0.695 (0.657, 0.734)	-0.370 (-0.426, -0.315), 0.000***, 0.690 (0.653, 0.730)
Marked Crossing	0.175 (0.042, 0.307), 0.010**, 1.191 (1.043, 1.359)	0.142 (0.010, 0.274), 0.036*, 1.152 (1.010, 1.315)	0.132 (-0.000, 0.264), 0.050, 1.141 (1.000, 1.302)	0.084 (-0.049, 0.217), 0.213, 1.088 (0.953, 1.243)
Uncontrolled Crossing	-0.461 (-0.566, -0.355), 0.000***, 0.631 (0.568, 0.701)	-0.417 (-0.523, -0.312), 0.000***, 0.659 (0.593, 0.732)	-0.415 (-0.520, -0.309), 0.000***, 0.661 (0.594, 0.734)	-0.465 (-0.571, -0.360), 0.000***, 0.628 (0.565, 0.698)
Roundabout	0.111 (0.039, 0.182), 0.002**, 1.117 (1.040, 1.200)	0.143 (0.071, 0.214), 0.000***, 1.153 (1.074, 1.239)	0.168 (0.097, 0.239), 0.000***, 1.183 (1.102, 1.270)	0.118 (0.047, 0.190), 0.001**, 1.126 (1.048, 1.209)
Mini Roundabout	-1.334 (-1.662, -1.005), 0.000***, 0.264 (0.190, 0.366)	-1.230 (-1.557, -0.902), 0.000***, 0.292 (0.211, 0.406)	-1.093 (-1.423, -0.763), 0.000***, 0.335 (0.241, 0.466)	-1.440 (-1.768, -1.113), 0.000***, 0.237 (0.171, 0.329)
Motorway Junction	0.929 (0.796, 1.061), 0.000***, 2.531 (2.217, 2.890)	0.944 (0.811, 1.077), 0.000***, 2.570 (2.251, 2.935)	0.992 (0.859, 1.125), 0.000***, 2.696 (2.361, 3.079)	0.936 (0.802, 1.069), 0.000***, 2.549 (2.230, 2.913)
Traffic Signal	-0.536 (-0.590, -0.481), 0.000***, 0.585 (0.554, 0.618)	-0.535 (-0.590, -0.481), 0.000***, 0.585 (0.554, 0.618)	-0.525 (-0.580, -0.471), 0.000***, 0.591 (0.560, 0.624)	-0.555 (-0.610, -0.500), 0.000***, 0.574 (0.544, 0.607)
Speed Camera	-0.204 (-0.341, -0.068), 0.003**, 0.815 (0.711, 0.934)	-0.265 (-0.402, -0.129), 0.000***, 0.767 (0.669, 0.879)	-0.245 (-0.382, -0.109), 0.000***, 0.782 (0.682, 0.897)	-0.304 (-0.441, -0.166), 0.000***, 0.738 (0.643, 0.847)
Link Bidirection	-0.675 (-0.724, -0.625), 0.000***, 0.509 (0.485, 0.535)	-0.842 (-0.891, -0.793), 0.000***, 0.431 (0.410, 0.452)	-0.857 (-0.911, -0.804), 0.000***, 0.424 (0.402, 0.448)	-0.765 (-0.819, -0.711), 0.000***, 0.465 (0.441, 0.491)
Link Average Width	-0.038 (-0.062, -0.015), 0.002**, 0.962 (0.940, 0.986)	-0.050 (-0.073, -0.026), 0.000***, 0.951 (0.929, 0.974)	-0.050 (-0.074, -0.027), 0.000***, 0.951 (0.929, 0.974)	-0.056 (-0.080, -0.033), 0.000***, 0.945 (0.923, 0.968)
Link Length	1.666 (1.631, 1.702), 0.000***, 5.293 (5.110, 5.482)	1.739 (1.703, 1.774), 0.000***, 5.689 (5.491, 5.895)	1.724 (1.690, 1.759), 0.000***, 5.607 (5.417, 5.804)	1.751 (1.717, 1.785), 0.000***, 5.760 (5.566, 5.961)
Link Angular Curvature	-0.665 (-0.692, -0.637), 0.000***, 0.514 (0.500, 0.529)	-0.683 (-0.710, -0.656), 0.000***, 0.505 (0.492, 0.519)	-0.687 (-0.714, -0.660), 0.000***, 0.503 (0.490, 0.517)	-0.674 (-0.701, -0.648), 0.000***, 0.509 (0.496, 0.523)
Link Degree	0.149 (0.127, 0.171), 0.000***, 1.161 (1.136, 1.186)	0.149 (0.128, 0.170), 0.000***, 1.160 (1.136, 1.185)	0.129 (0.109, 0.149), 0.000***, 1.137 (1.115, 1.161)	0.119 (0.099, 0.140), 0.000***, 1.127 (1.104, 1.150)
Connectivity	-0.359 (-0.384, -0.334), 0.000***, 0.699 (0.681, 0.716)	-0.334 (-0.359, -0.310), 0.000***, 0.716 (0.699, 0.734)	-0.289 (-0.317, -0.261), 0.000***, 0.749 (0.728, 0.770)	-0.187 (-0.218, -0.155), 0.000***, 0.830 (0.804, 0.857)
Betweenness	0.096 (0.070, 0.123), 0.000***, 1.101 (1.072, 1.131)	0.063 (0.037, 0.090), 0.000***, 1.065 (1.037, 1.094)	-0.007 (-0.034, 0.020), 0.604, 0.993 (0.967, 1.020)	0.096 (0.068, 0.123), 0.000***, 1.100 (1.070, 1.131)
Closeness	0.256 (0.117, 0.395), 0.000***, 1.291 (1.124, 1.484)	0.020 (-0.017, 0.056), 0.285, 1.020 (0.984, 1.058)	0.199 (0.069, 0.330), 0.003**, 1.221 (1.071, 1.391)	0.467 (0.312, 0.622), 0.000***, 1.595 (1.366, 1.862)
Average Shortest Path	0.147 (0.123, 0.171), 0.000***, 1.159 (1.131, 1.186)	0.135 (0.114, 0.156), 0.000***, 1.145 (1.121, 1.169)	0.206 (0.186, 0.225), 0.000***, 1.228 (1.204, 1.253)	0.145 (0.126, 0.163), 0.000***, 1.156 (1.134, 1.178)
Diversion Ratio	0.059 (0.039, 0.079), 0.000***, 1.060 (1.039, 1.082)	-0.064 (-0.082, -0.046), 0.000***, 0.938 (0.921, 0.955)	0.017 (-0.007, 0.042), 0.170, 1.018 (0.993, 1.043)	0.185 (0.160, 0.210), 0.000***, 1.203 (1.173, 1.234)
Random effect - City	0.19	0.19	0.18	0.19
ICC	0.12	0.12	0.12	0.12
Marginal R^2 / Conditional R^2	0.652 / 0.695	0.648 / 0.690	0.646 / 0.688	0.655 / 0.698

Note: This table reports results from negative binomial count models of observed speeding events aggregated at the road-link level for roads with a posted speed limit of 40 mph. The dependent variable is the total number of speeding events per link. Network metrics (connectivity, betweenness, closeness, average shortest path, and diversion ratio) are measured at alternative network distance thresholds of 400 m, 800 m, 2 km, and 5 km, while all other covariates remain unchanged across model specifications. No adjustment is made for traffic exposure or GPS observation intensity. Results are reported as incidence rate ratios (IRR) with 95% confidence intervals and p-values.

Table S14 Negative binomial count model results for speeding events per road link on 50 mph roads, with network metrics measured at different network distances (400 m, 800 m, 2 km, and 5 km)

	Speeding events per road link (Coefficients, 95% CI, p-value, IRR, 95% CI)			
Independent variables	50m_400m	50m_800m	50m_2km	50m_5km
Traffic Calming	0.329 (-0.153, 0.812), 0.181, 1.390 (0.858, 2.252)	0.359 (-0.123, 0.841), 0.145, 1.432 (0.884, 2.319)	0.387 (-0.099, 0.873), 0.119, 1.473 (0.906, 2.394)	0.243 (-0.242, 0.728), 0.327, 1.275 (0.785, 2.071)
Choker	-0.341 (-1.731, 1.050), 0.631, 0.711 (0.177, 2.857)	-0.356 (-1.742, 1.030), 0.615, 0.700 (0.175, 2.801)	-0.381 (-1.778, 1.015), 0.592, 0.683 (0.169, 2.760)	-0.485 (-1.883, 0.913), 0.497, 0.616 (0.152, 2.492)
Island	-0.728 (-0.942, -0.514), 0.000***, 0.483 (0.390, 0.598)	-0.684 (-0.900, -0.469), 0.000***, 0.505 (0.407, 0.626)	-0.704 (-0.920, -0.487), 0.000***, 0.495 (0.398, 0.614)	-0.718 (-0.935, -0.501), 0.000***, 0.488 (0.392, 0.606)
Signalised Crossing	-0.583 (-0.770, -0.396), 0.000***, 0.558 (0.463, 0.673)	-0.560 (-0.747, -0.373), 0.000***, 0.571 (0.474, 0.689)	-0.607 (-0.799, -0.415), 0.000***, 0.545 (0.450, 0.660)	-0.614 (-0.806, -0.422), 0.000***, 0.541 (0.447, 0.656)
Marked Crossing	-1.005 (-1.420, -0.591), 0.000***, 0.366 (0.242, 0.554)	-0.928 (-1.341, -0.514), 0.000***, 0.396 (0.261, 0.598)	-0.966 (-1.382, -0.549), 0.000***, 0.381 (0.251, 0.577)	-1.015 (-1.433, -0.596), 0.000***, 0.363 (0.239, 0.551)
Uncontrolled Crossing	-0.332 (-0.638, -0.027), 0.033*, 0.717 (0.528, 0.973)	-0.316 (-0.620, -0.011), 0.043*, 0.729 (0.538, 0.989)	-0.301 (-0.609, 0.006), 0.055, 0.740 (0.544, 1.006)	-0.287 (-0.595, 0.022), 0.068, 0.751 (0.552, 1.022)
Roundabout	0.059 (-0.096, 0.215), 0.455, 1.061 (0.908, 1.240)	0.096 (-0.060, 0.252), 0.229, 1.101 (0.942, 1.287)	0.191 (0.033, 0.349), 0.018*, 1.211 (1.034, 1.418)	0.190 (0.032, 0.348), 0.019*, 1.209 (1.032, 1.416)
Mini Roundabout	0.169 (-0.408, 0.746), 0.565, 1.184 (0.665, 2.109)	0.072 (-0.505, 0.649), 0.806, 1.075 (0.604, 1.914)	0.126 (-0.453, 0.706), 0.669, 1.135 (0.636, 2.025)	0.116 (-0.465, 0.696), 0.696, 1.123 (0.628, 2.006)
Motorway Junction	0.523 (0.361, 0.686), 0.000***, 1.688 (1.434, 1.986)	0.512 (0.349, 0.675), 0.000***, 1.668 (1.417, 1.964)	0.517 (0.353, 0.681), 0.000***, 1.677 (1.424, 1.976)	0.492 (0.328, 0.656), 0.000***, 1.635 (1.388, 1.926)
Traffic Signal	-0.231 (-0.390, -0.073), 0.004**, 0.793 (0.677, 0.930)	-0.252 (-0.411, -0.092), 0.002**, 0.778 (0.663, 0.912)	-0.272 (-0.435, -0.109), 0.001**, 0.762 (0.648, 0.897)	-0.298 (-0.460, -0.135), 0.000***, 0.743 (0.631, 0.874)
Speed Camera	0.308 (-0.045, 0.660), 0.087, 1.360 (0.956, 1.935)	0.290 (-0.062, 0.643), 0.106, 1.337 (0.940, 1.902)	0.354 (-0.001, 0.709), 0.051, 1.425 (0.999, 2.031)	0.318 (-0.037, 0.672), 0.079, 1.374 (0.964, 1.959)
Link Bidirection	-1.469 (-1.605, -1.332), 0.000***, 0.230 (0.201, 0.264)	-1.618 (-1.751, -1.484), 0.000***, 0.198 (0.174, 0.227)	-1.672 (-1.814, -1.530), 0.000***, 0.188 (0.163, 0.217)	-1.755 (-1.905, -1.606), 0.000***, 0.173 (0.149, 0.201)
Link Average Width	0.332 (0.269, 0.395), 0.000***, 1.394 (1.308, 1.485)	0.330 (0.267, 0.393), 0.000***, 1.391 (1.306, 1.481)	0.335 (0.272, 0.399), 0.000***, 1.398 (1.312, 1.490)	0.331 (0.267, 0.395), 0.000***, 1.392 (1.306, 1.484)
Link Length	1.921 (1.833, 2.009), 0.000***, 6.828 (6.255, 7.455)	1.878 (1.793, 1.964), 0.000***, 6.543 (6.005, 7.129)	1.972 (1.887, 2.056), 0.000***, 7.182 (6.602, 7.812)	1.994 (1.910, 2.078), 0.000***, 7.346 (6.754, 7.989)
Link Angular Curvature	-0.769 (-0.830, -0.707), 0.000***, 0.464 (0.436, 0.493)	-0.748 (-0.810, -0.687), 0.000***, 0.473 (0.445, 0.503)	-0.772 (-0.833, -0.710), 0.000***, 0.462 (0.435, 0.492)	-0.764 (-0.825, -0.703), 0.000***, 0.466 (0.438, 0.495)
Link Degree	0.266 (0.215, 0.316), 0.000***, 1.304 (1.240, 1.372)	0.211 (0.158, 0.263), 0.000***, 1.235 (1.171, 1.301)	0.229 (0.181, 0.277), 0.000***, 1.258 (1.198, 1.319)	0.219 (0.171, 0.267), 0.000***, 1.245 (1.187, 1.306)
Connectivity	-0.288 (-0.344, -0.233), 0.000***, 0.750 (0.709, 0.793)	-0.317 (-0.376, -0.258), 0.000***, 0.729 (0.687, 0.773)	-0.137 (-0.207, -0.067), 0.000***, 0.872 (0.813, 0.935)	-0.221 (-0.304, -0.138), 0.000***, 0.801 (0.738, 0.871)
Betweenness	-0.058 (-0.127, 0.010), 0.096, 0.943 (0.881, 1.010)	0.130 (0.068, 0.192), 0.000***, 1.139 (1.071, 1.212)	-0.052 (-0.117, 0.013), 0.114, 0.949 (0.889, 1.013)	0.083 (0.015, 0.151), 0.016*, 1.086 (1.015, 1.163)
Closeness	0.073 (-0.039, 0.184), 0.200, 1.075 (0.962, 1.202)	0.354 (0.171, 0.536), 0.000***, 1.424 (1.187, 1.709)	0.172 (0.026, 0.319), 0.021*, 1.188 (1.026, 1.376)	0.107 (-0.020, 0.234), 0.098, 1.113 (0.981, 1.264)
Average Shortest Path	0.117 (0.055, 0.179), 0.000***, 1.124 (1.056, 1.196)	0.113 (0.055, 0.171), 0.000***, 1.119 (1.056, 1.186)	0.054 (0.007, 0.102), 0.024*, 1.056 (1.007, 1.107)	0.013 (-0.031, 0.057), 0.565, 1.013 (0.969, 1.059)
Diversion Ratio	0.129 (0.080, 0.178), 0.000***, 1.138 (1.083, 1.195)	0.182 (0.126, 0.239), 0.000***, 1.200 (1.134, 1.270)	-0.004 (-0.064, 0.055), 0.885, 0.996 (0.938, 1.056)	0.032 (-0.027, 0.090), 0.293, 1.032 (0.973, 1.094)
Random effect - City	0.47	0.44	0.41	0.39
ICC	0.24	0.23	0.22	0.21
Marginal R^2 / Conditional R^2	0.670 / 0.750	0.676 / 0.750	0.671 / 0.742	0.675 / 0.743

Note: This table reports results from negative binomial count models of observed speeding events aggregated at the road-link level for roads with a posted speed limit of 50 mph. The dependent variable is the total number of speeding events per link. Network metrics (connectivity, betweenness, closeness, average shortest path, and diversion ratio) are measured at alternative network distance thresholds of 400 m, 800 m, 2 km, and 5 km, while all other covariates remain unchanged across model specifications. No adjustment is made for traffic exposure or GPS observation intensity. Results are reported as incidence rate ratios (IRR) with 95% confidence intervals and p-values.

Table S15 Negative binomial count model results for speeding events per road link on 60 mph roads, with network metrics measured at different network distances (400 m, 800 m, 2 km, and 5 km)

	Speeding events per road link (Coefficients, 95% CI, p-value, IRR, 95% CI)			
Independent variables	50m_400m	50m_800m	50m_2km	50m_5km
Traffic Calming	-1.681 (-2.742, -0.620), 0.002**, 0.186 (0.064, 0.538)	-1.834 (-2.960, -0.708), 0.001**, 0.160 (0.052, 0.492)	-1.723 (-2.804, -0.643), 0.002**, 0.178 (0.061, 0.526)	-1.701 (-2.766, -0.637), 0.002**, 0.182 (0.063, 0.529)
Choker	/	/	/	/
Island	0.547 (-0.028, 1.121), 0.062, 1.727 (0.973, 3.067)	0.609 (0.029, 1.189), 0.040*, 1.838 (1.029, 3.282)	0.699 (0.076, 1.321), 0.028*, 2.011 (1.079, 3.748)	0.670 (0.051, 1.289), 0.034*, 1.955 (1.053, 3.630)
Signalised Crossing	-2.292 (-2.866, -1.718), 0.000***, 0.101 (0.057, 0.180)	-2.083 (-2.651, -1.515), 0.000***, 0.125 (0.071, 0.220)	-2.171 (-2.749, -1.592), 0.000***, 0.114 (0.064, 0.203)	-2.291 (-2.873, -1.709), 0.000***, 0.101 (0.057, 0.181)
Marked Crossing	/	/	/	/
Uncontrolled Crossing	-0.610 (-1.559, 0.339), 0.208, 0.543 (0.210, 1.404)	-0.515 (-1.457, 0.428), 0.284, 0.598 (0.233, 1.534)	-0.053 (-0.992, 0.886), 0.912, 0.948 (0.371, 2.425)	-0.173 (-1.126, 0.781), 0.723, 0.841 (0.324, 2.184)
Roundabout	0.624 (0.216, 1.031), 0.003**, 1.866 (1.241, 2.804)	0.857 (0.451, 1.263), 0.000***, 2.355 (1.569, 3.535)	0.878 (0.451, 1.306), 0.000***, 2.407 (1.570, 3.690)	0.609 (0.181, 1.038), 0.005**, 1.839 (1.199, 2.823)
Mini Roundabout	-1.491 (-2.432, -0.550), 0.002**, 0.225 (0.088, 0.577)	-1.252 (-2.197, -0.307), 0.009**, 0.286 (0.111, 0.735)	-1.486 (-2.434, -0.537), 0.002**, 0.226 (0.088, 0.584)	-1.094 (-2.030, -0.157), 0.022*, 0.335 (0.131, 0.855)
Motorway Junction	-0.148 (-0.642, 0.347), 0.558, 0.863 (0.526, 1.414)	-0.205 (-0.694, 0.283), 0.410, 0.814 (0.500, 1.327)	-0.098 (-0.591, 0.395), 0.698, 0.907 (0.554, 1.485)	-0.011 (-0.513, 0.490), 0.965, 0.989 (0.599, 1.633)
Traffic Signal	1.008 (0.604, 1.412), 0.000***, 2.740 (1.829, 4.106)	1.037 (0.641, 1.433), 0.000***, 2.821 (1.899, 4.191)	1.312 (0.914, 1.710), 0.000***, 3.714 (2.495, 5.529)	1.471 (1.070, 1.871), 0.000***, 4.352 (2.915, 6.497)
Speed Camera	1.947 (0.650, 3.244), 0.003**, 7.006 (1.915, 25.630)	1.465 (0.247, 2.684), 0.018*, 4.329 (1.280, 14.641)	1.452 (0.211, 2.692), 0.022*, 4.270 (1.235, 14.767)	1.293 (0.048, 2.539), 0.042*, 3.645 (1.049, 12.670)
Link Bidirection	-3.000 (-3.305, -2.696), 0.000***, 0.050 (0.037, 0.067)	-3.264 (-3.564, -2.964), 0.000***, 0.038 (0.028, 0.052)	-3.366 (-3.684, -3.047), 0.000***, 0.035 (0.025, 0.048)	-3.474 (-3.802, -3.147), 0.000***, 0.031 (0.022, 0.043)
Link Average Width	1.235 (1.136, 1.334), 0.000***, 3.438 (3.113, 3.796)	1.254 (1.155, 1.353), 0.000***, 3.504 (3.175, 3.868)	1.263 (1.163, 1.363), 0.000***, 3.536 (3.199, 3.909)	1.272 (1.171, 1.374), 0.000***, 3.570 (3.226, 3.950)
Link Length	1.807 (1.688, 1.926), 0.000***, 6.091 (5.406, 6.862)	1.830 (1.724, 1.936), 0.000***, 6.233 (5.608, 6.929)	1.958 (1.854, 2.063), 0.000***, 7.086 (6.383, 7.866)	2.035 (1.932, 2.139), 0.000***, 7.656 (6.903, 8.491)
Link Angular Curvature	-2.529 (-2.683, -2.376), 0.000***, 0.080 (0.068, 0.093)	-2.432 (-2.584, -2.280), 0.000***, 0.088 (0.076, 0.102)	-2.397 (-2.551, -2.243), 0.000***, 0.091 (0.078, 0.106)	-2.427 (-2.581, -2.273), 0.000***, 0.088 (0.076, 0.103)
Link Degree	0.232 (0.166, 0.299), 0.000***, 1.262 (1.181, 1.348)	0.168 (0.102, 0.234), 0.000***, 1.183 (1.107, 1.263)	0.075 (0.011, 0.139), 0.022*, 1.078 (1.011, 1.150)	0.028 (-0.033, 0.089), 0.367, 1.028 (0.968, 1.093)
Connectivity	-0.486 (-0.585, -0.387), 0.000***, 0.615 (0.557, 0.679)	-0.438 (-0.524, -0.351), 0.000***, 0.646 (0.592, 0.704)	-0.294 (-0.374, -0.215), 0.000***, 0.745 (0.688, 0.807)	-0.494 (-0.594, -0.395), 0.000***, 0.610 (0.552, 0.674)
Betweenness	-0.087 (-0.186, 0.013), 0.087, 0.917 (0.831, 1.013)	-0.054 (-0.141, 0.033), 0.226, 0.948 (0.868, 1.034)	0.031 (-0.059, 0.120), 0.500, 1.031 (0.943, 1.127)	0.276 (0.183, 0.369), 0.000***, 1.318 (1.201, 1.446)
Closeness	0.304 (0.015, 0.593), 0.039*, 1.355 (1.015, 1.809)	0.149 (-0.137, 0.435), 0.306, 1.161 (0.872, 1.545)	0.132 (-0.081, 0.345), 0.226, 1.141 (0.922, 1.412)	0.012 (-0.110, 0.134), 0.844, 1.012 (0.896, 1.143)
Average Shortest Path	0.353 (0.248, 0.458), 0.000***, 1.424 (1.282, 1.581)	0.427 (0.357, 0.498), 0.000***, 1.533 (1.429, 1.645)	0.405 (0.339, 0.472), 0.000***, 1.500 (1.404, 1.602)	0.279 (0.226, 0.332), 0.000***, 1.322 (1.254, 1.393)
Diversion Ratio	0.095 (0.020, 0.170), 0.013*, 1.100 (1.020, 1.185)	-0.042 (-0.101, 0.017), 0.162, 0.959 (0.904, 1.017)	-0.139 (-0.193, -0.086), 0.000***, 0.870 (0.825, 0.918)	-0.092 (-0.164, -0.021), 0.011*, 0.912 (0.849, 0.979)
Random effect - City	2.67	1.97	2.35	2.51
ICC	0.56	0.49	0.53	0.55
Marginal R^2 / Conditional R^2	0.674 / 0.858	0.709 / 0.852	0.682 / 0.851	0.671 / 0.851

Note: This table reports results from negative binomial count models of observed speeding events aggregated at the road-link level for roads with a posted speed limit of 60 mph. The dependent variable is the total number of speeding events per link. Network metrics (connectivity, betweenness, closeness, average shortest path, and diversion ratio) are measured at alternative network distance thresholds of 400 m, 800 m, 2 km, and 5 km, while all other covariates remain unchanged across model specifications. No adjustment is made for traffic exposure or GPS observation intensity. Results are reported as incidence rate ratios (IRR) with 95% confidence intervals and p-values.

Table S16 Negative binomial count model results for speeding events per road link on 70 mph roads, with network metrics measured at different network distances (400 m, 800 m, 2 km, and 5 km)

	Speeding events per road link (Coefficients, 95% CI, p-value, IRR, 95% CI)			
Independent variables	50m_400m	50m_800m	50m_2km	50m_5km
Traffic Calming	-0.397 (-1.112, 0.319), 0.277, 0.673 (0.329, 1.375)	-0.337 (-1.060, 0.387), 0.362, 0.714 (0.347, 1.472)	-0.271 (-1.004, 0.461), 0.468, 0.762 (0.367, 1.586)	-0.159 (-0.885, 0.567), 0.668, 0.853 (0.413, 1.763)
Choker	/	/	/	/
Island	-0.071 (-0.464, 0.322), 0.723, 0.931 (0.629, 1.380)	-0.122 (-0.524, 0.281), 0.554, 0.885 (0.592, 1.325)	-0.128 (-0.534, 0.277), 0.536, 0.880 (0.586, 1.320)	0.093 (-0.315, 0.502), 0.654, 1.098 (0.730, 1.652)
Signalised Crossing	-0.500 (-0.770, -0.230), 0.000***, 0.607 (0.463, 0.795)	-0.495 (-0.774, -0.216), 0.001***, 0.610 (0.461, 0.806)	-0.465 (-0.746, -0.184), 0.001**, 0.628 (0.474, 0.832)	-0.455 (-0.736, -0.173), 0.002**, 0.635 (0.479, 0.841)
Marked Crossing	-0.385 (-1.073, 0.303), 0.273, 0.681 (0.342, 1.354)	-0.204 (-0.907, 0.498), 0.569, 0.815 (0.404, 1.646)	-0.212 (-0.924, 0.500), 0.560, 0.809 (0.397, 1.649)	-0.275 (-0.985, 0.434), 0.447, 0.759 (0.374, 1.543)
Uncontrolled Crossing	-1.038 (-1.479, -0.597), 0.000***, 0.354 (0.228, 0.551)	-0.859 (-1.314, -0.405), 0.000***, 0.423 (0.269, 0.667)	-0.951 (-1.410, -0.492), 0.000***, 0.386 (0.244, 0.612)	-0.914 (-1.375, -0.453), 0.000***, 0.401 (0.253, 0.636)
Roundabout	0.114 (-0.018, 0.247), 0.091, 1.121 (0.982, 1.280)	0.083 (-0.051, 0.216), 0.227, 1.086 (0.950, 1.242)	0.096 (-0.041, 0.233), 0.172, 1.100 (0.959, 1.262)	0.111 (-0.022, 0.244), 0.101, 1.118 (0.978, 1.277)
Mini Roundabout	-0.728 (-1.387, -0.069), 0.030*, 0.483 (0.250, 0.933)	-0.822 (-1.492, -0.153), 0.016*, 0.439 (0.225, 0.858)	-0.560 (-1.240, 0.120), 0.107, 0.571 (0.289, 1.128)	-0.567 (-1.241, 0.108), 0.100, 0.568 (0.289, 1.114)
Motorway Junction	-0.051 (-0.166, 0.063), 0.379, 0.950 (0.847, 1.065)	-0.043 (-0.158, 0.072), 0.464, 0.958 (0.854, 1.075)	-0.085 (-0.201, 0.031), 0.151, 0.919 (0.818, 1.031)	-0.080 (-0.195, 0.034), 0.169, 0.923 (0.823, 1.035)
Traffic Signal	-0.101 (-0.244, 0.042), 0.165, 0.904 (0.783, 1.042)	-0.082 (-0.229, 0.064), 0.271, 0.921 (0.796, 1.066)	-0.061 (-0.209, 0.087), 0.422, 0.941 (0.812, 1.091)	-0.009 (-0.154, 0.137), 0.907, 0.991 (0.857, 1.146)
Speed Camera	-0.353 (-0.764, 0.058), 0.092, 0.703 (0.466, 1.059)	-0.457 (-0.876, -0.038), 0.033*, 0.633 (0.416, 0.963)	-0.250 (-0.673, 0.174), 0.248, 0.779 (0.510, 1.190)	-0.248 (-0.674, 0.179), 0.255, 0.781 (0.510, 1.196)
Link Bidirection	/	/	/	/
Link Average Width	0.676 (0.609, 0.742), 0.000***, 1.965 (1.839, 2.100)	0.697 (0.630, 0.765), 0.000***, 2.009 (1.877, 2.149)	0.684 (0.615, 0.754), 0.000***, 1.983 (1.851, 2.124)	0.716 (0.646, 0.785), 0.000***, 2.046 (1.908, 2.193)
Link Length	2.133 (2.034, 2.232), 0.000***, 8.442 (7.646, 9.322)	1.611 (1.531, 1.691), 0.000***, 5.006 (4.621, 5.423)	1.723 (1.631, 1.814), 0.000***, 5.599 (5.111, 6.135)	1.686 (1.604, 1.768), 0.000***, 5.397 (4.971, 5.860)
Link Angular Curvature	-0.512 (-0.570, -0.454), 0.000***, 0.599 (0.565, 0.635)	-0.471 (-0.530, -0.413), 0.000***, 0.624 (0.589, 0.662)	-0.445 (-0.503, -0.386), 0.000***, 0.641 (0.604, 0.680)	-0.434 (-0.493, -0.375), 0.000***, 0.648 (0.611, 0.688)
Link Degree	0.317 (0.268, 0.365), 0.000***, 1.373 (1.308, 1.441)	0.304 (0.249, 0.359), 0.000***, 1.355 (1.282, 1.433)	0.276 (0.224, 0.328), 0.000***, 1.318 (1.252, 1.388)	0.217 (0.170, 0.265), 0.000***, 1.243 (1.185, 1.303)
Connectivity	-0.218 (-0.275, -0.161), 0.000***, 0.804 (0.760, 0.852)	-0.154 (-0.209, -0.099), 0.000***, 0.857 (0.811, 0.906)	-0.124 (-0.188, -0.061), 0.000***, 0.883 (0.829, 0.941)	-0.319 (-0.394, -0.243), 0.000***, 0.727 (0.675, 0.784)
Betweenness	-0.564 (-0.618, -0.511), 0.000***, 0.569 (0.539, 0.600)	-0.233 (-0.300, -0.165), 0.000***, 0.792 (0.741, 0.848)	-0.126 (-0.194, -0.058), 0.000***, 0.882 (0.824, 0.944)	-0.081 (-0.150, -0.011), 0.023*, 0.922 (0.861, 0.989)
Closeness	-0.075 (-0.121, -0.028), 0.002**, 0.928 (0.886, 0.972)	-0.126 (-0.209, -0.044), 0.003**, 0.881 (0.811, 0.957)	-0.055 (-0.136, 0.025), 0.178, 0.946 (0.873, 1.026)	-0.071 (-0.147, 0.004), 0.063, 0.931 (0.864, 1.004)
Average Shortest Path	-0.273 (-0.355, -0.191), 0.000***, 0.761 (0.701, 0.826)	-0.016 (-0.067, 0.036), 0.552, 0.985 (0.936, 1.036)	0.024 (-0.023, 0.071), 0.318, 1.024 (0.977, 1.074)	0.080 (0.031, 0.128), 0.001**, 1.083 (1.032, 1.137)
Diversion Ratio	0.144 (-0.008, 0.296), 0.063, 1.155 (0.992, 1.345)	0.297 (0.231, 0.363), 0.000***, 1.346 (1.260, 1.437)	-0.149 (-0.208, -0.090), 0.000***, 0.862 (0.812, 0.914)	-0.206 (-0.258, -0.154), 0.000***, 0.814 (0.772, 0.857)
Random effect - City	0.36	0.37	0.37	0.36
ICC	0.23	0.23	0.23	0.22
Marginal R^2 / Conditional R^2	0.651 / 0.731	0.658 / 0.737	0.666 / 0.742	0.676 / 0.747

Note: This table reports results from negative binomial count models of observed speeding events aggregated at the road-link level for roads with a posted speed limit of 70 mph. The dependent variable is the total number of speeding events per link. Network metrics (connectivity, betweenness, closeness, average shortest path, and diversion ratio) are measured at alternative network distance thresholds of 400 m, 800 m, 2 km, and 5 km, while all other covariates remain unchanged across model specifications. No adjustment is made for traffic exposure or GPS observation intensity. Results are reported as incidence rate ratios (IRR) with 95% confidence intervals and p-values.

PSM and GPS causal model results

Table S17 Causal analysis results for treatment variables at 400 m showing the average treatment effect on the treated (ATT) for speeding rate (%) and speeding events

**Binary treatment variables (ATT between 0 and 1, PSM)**	Total Obs.	ATT for Speeding rate (%)	ATT for Speeding event	Matched treated / control obs.	Average balance
Traffic calming	871524	(13.299, 7.719), −5.580	(30.065, 20.215), −9.851	113620 / 113620	0.003, (<0.1)
Choker	871524	(10.895, 9.206), −1.689	(25.272, 20.410), −4.862	6327 / 6327	0.005, (<0.1)
Traffic island	871524	(5.816, 7.347), +1.531	(29.983, 45.774), +15.791	50580 / 50580	0.007, (<0.1)
Signalised crossing	871524	(7.010, 6.472), −0.538	(40.520, 51.308), +10.787	63678 / 63678	0.015, (<0.1)
Marked crossing	871524	(7.131, 6.331), −0.799	(28.679, 30.417), +1.738	19670 / 19670	0.011, (<0.1)
Uncontrolled crossing	871524	(8.768, 7.324), −1.445	(27.830, 36.261), +8.430	37930 / 37930	0.007, (<0.1)
Roundabout	871524	(5.093, 5.962), +0.869	(79.613, 76.754), −2.859	23381 / 23381	0.008, (<0.1)
Mini roundabout	871524	(8.452, 6.618), −1.834	(27.703, 23.061), −4.641	19278 / 19278	0.009, (<0.1)
Motorway junction	871524	(6.623, 10.984), +4.361	(417.064, 555.641), +138.576	3166 / 3166	0.020, (<0.1)
Traffic signal	871524	(8.624, 5.359), −3.265	(65.548, 49.859), −15.689	67120 / 67120	0.015, (<0.1)
Speed camera	871524	(7.203, 6.094), −1.109	(84.747, 133.550), +48.802	3421 / 3421	0.018, (<0.1)
Link bidirection	871524	(7.174, 6.022), −1.152	(81.089, 32.521), −48.569	95473 / 95473	0.095, (<0.1)
**Continuous treatment variables (ATT between mean and mean +1 SD, GPS)**	Trim treated (0.05, 0.95), Weight, Trim GPS	ATT for speeding rate (%)	ATT for speeding event	Effective sample size	Average balance
Link average width	785276 / trim gps (0.001, 0.999), 783704	(8.283, 9.290), +1.008	(27.953, 41.524), +13.571	148336	0.014, (<0.1)
Link length	784371 / trim gps (0.005, 0.995), 776527	(8.301, 9.766), +1.466	(20.279, 50.416), +30.136	24600	0.033, (<0.1)
Link angular curvature	827947 / matching	(8.978, 7.050), −1.928	(41.711, 31.161), −10.549	2700	0.093, (<0.1)
Link degree	837019 / matching	(8.470, 7.625), −0.845	(29.758, 29.956), +0.198	26213	0.029, (<0.1)
Connectivity	798676 / trim gps (0.001, 0.999), 797078	(7.422, 7.251), −0.171	(18.788, 16.146), −2.641	12209	0.038, (<0.1)
Betweenness	784370 / trim gps (0.005, 0.995), 776526	(7.817, 9.601), +1.784	(22.859, 24.052), +1.193	31234	0.053, (<0.1)
Closeness	784370 / trim gps (0.005, 0.995), 776526	(7.481, 7.922), +0.441	(22.071, 24.230), +2.160	73264	0.020, (<0.1)
Average shortest path	784370 / trim gps (0.005, 0.995), 776526	(7.268, 9.289), +2.021	(18.296, 24.435), +6.139	81328	0.034, (<0.1)
Diversion ratio	784370 / trim gps (0.005, 0.995), 776526	(7.780, 7.125), −0.655	(19.594, 17.954), −1.639	127170	0.018, (<0.1)

Note: This table reports causal estimates of the average treatment effect on the treated (ATT) for speeding rate and speeding events at a 400 m road-network distance. Binary treatments were evaluated using propensity score matching, while continuous treatments employed generalised propensity score models. Continuous effects are reported as mean and mean + 1 SD responses derived from exposure–response functions. For the treatment variable, all models adjust for covariates including road engineering features, road attributes, network metrics, and posted speed limits. Average balance values below 0.1 indicate acceptable covariate balance.

Table S18 Causal analysis results for treatment variables at 800 m showing the average treatment effect on the treated (ATT) for speeding rate (%) and speeding events

**Binary treatment variables (ATT between 0 and 1, PSM)**	Total Obs.	ATT for Speeding rate (%)	ATT for Speeding event	Matched treated / control obs.	Average balance
Traffic calming	871524	(13.236, 7.719), −5.517	(29.833, 20.196), −9.637	113585 / 113585	0.003, (<0.1)
Choker	871524	(10.633, 9.206), −1.427	(21.517, 20.410), −1.107	6327 / 6327	0.009, (<0.1)
Traffic island	871524	(6.026, 7.347), +1.321	(30.352, 45.774), +15.422	50580 / 50580	0.009, (<0.1)
Signalised crossing	871524	(7.007, 6.455), −0.552	(41.007, 51.723), +10.716	63948 / 63948	0.015, (<0.1)
Marked crossing	871524	(6.958, 6.334), −0.624	(29.420, 30.436), +1.016	19658 / 19658	0.010, (<0.1)
Uncontrolled crossing	871524	(8.841, 7.323), −1.518	(28.044, 36.260), +8.216	37932 / 37932	0.009, (<0.1)
Roundabout	871524	(4.981, 5.962), +0.982	(85.988, 76.757), −9.231	23380 / 23380	0.006, (<0.1)
Mini roundabout	871524	(8.650, 6.617), −2.033	(29.296, 23.058), −6.238	19281 / 19281	0.008, (<0.1)
Motorway junction	871524	(7.060, 10.984), +3.924	(418.385, 556.131), +137.747	3165 / 3165	0.031, (<0.1)
Traffic signal	871524	(8.689, 5.385), −3.304	(63.723, 50.292), −13.431	67124 / 67124	0.017, (<0.1)
Speed camera	871524	(7.596, 6.095), −1.502	(90.161, 138.337), +48.177	3426 / 3426	0.018, (<0.1)
Link bidirection	871524	(7.817, 6.085), −1.732	(70.763, 21.911), −48.852	76763 / 76763	0.045, (<0.1)
**Continuous treatment variables (ATT between mean and mean +1 SD, GPS)**	Trim treated (0.05, 0.95), Weight, Trim GPS	ATT for speeding rate (%)	ATT for speeding event	Effective sample size	Average balance
Link average width	785276 / trim gps (0.001, 0.999), 783704	(8.293, 9.381), +1.088	(29.372, 44.267), +14.895	143214	0.014, (<0.1)
Link length	784371 / trim gps (0.001, 0.999), 782801	(7.920, 9.898), +1.978	(21.664, 62.759), +41.095	17551	0.019, (<0.1)
Link angular curvature	827947 / matching	(8.991, 7.206), −1.785	(42.973, 33.309), −9.663	5718	0.069, (<0.1)
Link degree	837019 / matching	(8.359, 6.963), −1.396	(29.619, 26.188), −3.431	27329	0.026, (<0.1)
Connectivity	786060 / trim gps (0.005, 0.995), 778198	(7.406, 6.229), −1.178	(20.414, 13.698), −6.716	34906	0.048, (<0.1)
Betweenness	784370 / trim gps (0.005, 0.995), 776526	(6.885, 9.277), +2.392	(20.927, 22.477), +1.550	24821	0.069, (<0.1)
Closeness	784371 / trim gps (0.005, 0.995), 776526	(7.408, 7.646), +0.239	(24.303, 24.222), −0.081	97883	0.031, (<0.1)
Average shortest path	784370 / trim gps (0.001, 0.999), 782800	(6.987, 7.994), +1.007	(19.136, 22.541), +3.405	60008	0.015, (<0.1)
Diversion ratio	784370 / trim gps (0.005, 0.995), 776526	(7.715, 6.924), −0.791	(21.715, 18.054), −3.661	145371	0.023, (<0.1)

Note: This table reports causal estimates of the average treatment effect on the treated (ATT) for speeding rate and speeding events at a 800 m road-network distance. Binary treatments were evaluated using propensity score matching, while continuous treatments employed generalised propensity score models. Continuous effects are reported as mean and mean + 1 SD responses derived from exposure–response functions. For the treatment variable, all models adjust for covariates including road engineering features, road attributes, network metrics, and posted speed limits. Average balance values below 0.1 indicate acceptable covariate balance.

Table S19 Causal analysis results for treatment variables at 2 km showing the average treatment effect on the treated (ATT) for speeding rate (%) and speeding events

**Binary treatment variables (ATT between 0 and 1, PSM)**	Total Obs.	ATT for Speeding rate (%)	ATT for Speeding event	Matched treated / control obs.	Average balance
Traffic calming	871524	(12.468, 7.720), −4.748	(29.265, 20.176), −9.089	113585 / 113585	0.003, (<0.1)
Choker	871524	(10.601, 9.206), −1.395	(23.919, 20.410), −3.509	6327 / 6327	0.007, (<0.1)
Traffic island	871524	(6.419, 7.347), +0.928	(31.479, 45.774), +14.295	50580 / 50580	0.008, (<0.1)
Signalised crossing	871524	(7.195, 6.450), −0.745	(42.402, 51.072), +8.671	64162 / 64162	0.014, (<0.1)
Marked crossing	871524	(6.943, 6.332), −0.611	(27.449, 30.418), +2.970	19670 / 19670	0.008, (<0.1)
Uncontrolled crossing	871524	(9.037, 7.323), −1.714	(30.452, 36.260), +5.808	37932 / 37932	0.008, (<0.1)
Roundabout	871524	(5.863, 5.962), +0.099	(76.879, 76.754), −0.125	23381 / 23381	0.006, (<0.1)
Mini roundabout	871524	(9.477, 6.618), −2.859	(30.712, 23.061), −7.651	19278 / 19278	0.011, (<0.1)
Motorway junction	871524	(6.802, 10.973), +4.171	(400.058, 557.723), +157.666	3169 / 3169	0.009, (<0.1)
Traffic signal	871524	(8.726, 5.417), −3.309	(62.669, 49.868), −12.801	66739 / 66739	0.015, (<0.1)
Speed camera	871524	(7.796, 6.098), −1.698	(99.222, 137.504), +38.282	3424 / 3424	0.015, (<0.1)
Link bidirection	871524	(7.693, 6.339), −1.355	(55.392, 27.149), −28.243	82842 / 82842	0.051, (<0.1)
**Continuous treatment variables (ATT between mean and mean +1 SD, GPS)**	Trim treated (0.05, 0.95), Weight, Trim GPS	ATT for speeding rate (%)	ATT for speeding event	Effective sample size	Average balance
Link average width	785276 / trim gps (0.001, 0.999), 783704	(8.200, 9.064), +0.864	(26.846, 38.345), +11.499	115435	0.019, (<0.1)
Link length	784371 / trim gps (0.001, 0.999), 782801	(7.904, 10.144), +2.240	(24.971, 60.546), +35.575	16234	0.031, (<0.1)
Link angular curvature	827947 / matching	(9.074, 7.068), −2.006	(43.391, 34.222), −9.169	8472	0.057, (<0.1)
Link degree	837019 / matching	(8.279, 7.031), −1.248	(28.646, 25.746), −2.900	64013	0.022, (<0.1)
Connectivity	784503 / trim gps (0.001, 0.999), 782933	(8.771, 5.995), −2.776	(25.789, 16.484), −9.305	19224	0.030, (<0.1)
Betweenness	784370 / trim gps (0.005, 0.995), 776526	(7.412, 11.402), +3.990	(22.698, 32.510), +9.813	36957	0.068, (<0.1)
Closeness	784371 / trim gps (0.001, 0.999), 782801	(7.636, 7.392), −0.245	(24.932, 20.777), −4.155	70361	0.017, (<0.1)
Average shortest path	784370 / trim gps (0.001, 0.999), 782800	(7.692, 8.643), +0.951	(20.373, 21.963), +1.590	306186	0.011, (<0.1)
Diversion ratio	784371 / trim gps (0.005, 0.995), 776527	(7.813, 7.422), −0.390	(23.726, 21.920), −1.807	80669	0.032, (<0.1)

Note: This table reports causal estimates of the average treatment effect on the treated (ATT) for speeding rate and speeding events at a 2 km road-network distance. Binary treatments were evaluated using propensity score matching, while continuous treatments employed generalised propensity score models. Continuous effects are reported as mean and mean + 1 SD responses derived from exposure–response functions. For the treatment variable, all models adjust for covariates including road engineering features, road attributes, network metrics, and posted speed limits. Average balance values below 0.1 indicate acceptable covariate balance.

Table S20 Causal analysis results for treatment variables at 5 km showing the average treatment effect on the treated (ATT) for speeding rate (%) and speeding events

**Binary treatment variables (ATT between 0 and 1, PSM)**	Total Obs.	ATT for Speeding rate (%)	ATT for Speeding event	Matched treated / control obs.	Average balance
Traffic calming	871524	(11.314, 7.721), −3.593	(28.444, 20.191), −8.253	113570 / 113570	0.005, (<0.1)
Choker	871524	(10.217, 9.206), −1.010	(23.565, 20.410), −3.155	6327 / 6327	0.006, (<0.1)
Traffic island	871524	(6.346, 7.347), +1.001	(31.492, 45.774), +14.282	50580 / 50580	0.009, (<0.1)
Signalised crossing	871524	(7.204, 6.459), −0.746	(42.360, 50.411), +8.051	63919 / 63919	0.011, (<0.1)
Marked crossing	871524	(7.263, 6.329), −0.934	(28.026, 30.402), +2.376	19681 / 19681	0.009, (<0.1)
Uncontrolled crossing	871524	(8.945, 7.324), −1.621	(31.194, 36.264), +5.071	37926 / 37926	0.009, (<0.1)
Roundabout	871524	(6.510, 5.962), −0.548	(71.754, 76.582), +4.828	23379 / 23379	0.007, (<0.1)
Mini roundabout	871524	(9.502, 6.616), −2.885	(30.542, 23.053), −7.488	19281 / 19281	0.013, (<0.1)
Motorway junction	871524	(6.695, 10.981), +4.286	(390.566, 555.577), +165.011	3169 / 3169	0.011, (<0.1)
Traffic signal	871524	(8.783, 5.386), −3.398	(60.964, 49.632), −11.331	66709 / 66709	0.013, (<0.1)
Speed camera	871524	(8.236, 6.096), −2.139	(91.812, 138.375), +46.564	3425 / 3425	0.016, (<0.1)
Link bidirection	871524	(7.819, 7.559), −0.260	(56.356, 29.787), −26.569	93307 / 93307	0.047, (<0.1)
**Continuous treatment variables (ATT between mean and mean +1 SD, GPS)**	Trim treated (0.05, 0.95), Weight, Trim GPS	ATT for speeding rate (%)	ATT for speeding event	Effective sample size	Average balance
Link average width	785276 / trim gps (0.001, 0.999), 783704	(8.193, 8.820), +0.628	(25.788, 34.240), +8.452	101729	0.021, (<0.1)
Link length	784371 / trim gps (0.001, 0.999), 782801	(7.877, 10.274), +2.398	(24.762, 59.557), +34.795	24794	0.029, (<0.1)
Link angular curvature	827947 / matching	(9.132, 7.133), −2.000	(42.602, 33.452), −9.149	9522	0.055, (<0.1)
Link degree	837019 / matching	(8.279, 7.149), −1.130	(28.843, 26.733), −2.110	75270	0.018, (<0.1)
Connectivity	784382 / trim gps (0.001, 0.999), 782812	(8.625, 6.410), −2.216	(24.461, 19.302), −5.159	101821	0.030, (<0.1)
Betweenness	784370 / trim gps (0.005, 0.995), 776526	(7.637, 12.117), +4.480	(25.772, 43.813), +18.041	45252	0.053, (<0.1)
Closeness	784370 / trim gps (0.001, 0.999), 782800	(7.884, 8.151), +0.266	(24.968, 20.754), −4.214	100532	0.022, (<0.1)
Average shortest path	784370 / trim gps (0.001, 0.999), 782800	(7.824, 8.809), +0.984	(21.110, 22.739), +1.629	298341	0.015, (<0.1)
Diversion ratio	784371 / trim gps (0.005, 0.995), 776527	(8.218, 8.518), +0.300	(23.282, 24.383), +1.100	103095	0.032, (<0.1)

Note: This table reports causal estimates of the average treatment effect on the treated (ATT) for speeding rate and speeding events at a 5 km road-network distance. Binary treatments were evaluated using propensity score matching, while continuous treatments employed generalised propensity score models. Continuous effects are reported as mean and mean + 1 SD responses derived from exposure–response functions. For the treatment variable, all models adjust for covariates including road engineering features, road attributes, network metrics, and posted speed limits. Average balance values below 0.1 indicate acceptable covariate balance.

Causal sensitivity results

Table S21 Sensitivity analysis of post-adjustment causal effect estimates using E-values for unmeasured confounding in exposure-adjusted speeding rates model

**Binary treatment variables**	400 m		800 m		2 km		5 km	
	IRR, (95% CI)	E-value	IRR, (95% CI)	E-value	IRR, (95% CI)	E-value	IRR, (95% CI)	E-value
Traffic calming	0.681, (0.67, 0.691)	2.300	0.686, (0.676, 0.697)	2.272	0.726, (0.715, 0.737)	2.099	0.787, (0.775, 0.799)	1.857
Choker	0.875, (0.824, 0.929)	1.546	0.884, (0.832, 0.939)	1.517	0.866, (0.816, 0.92)	1.577	0.896, (0.844, 0.952)	1.474
Traffic island	1.25, (1.222, 1.278)	1.808	1.203, (1.176, 1.23)	1.696	1.142, (1.117, 1.168)	1.546	1.151, (1.126, 1.177)	1.569
Signalised crossing	0.898, (0.88, 0.915)	1.471	0.899, (0.882, 0.917)	1.465	0.874, (0.858, 0.891)	1.550	0.868, (0.852, 0.885)	1.571
Marked crossing	0.92, (0.886, 0.955)	1.396	0.94, (0.905, 0.975)	1.326	0.951, (0.916, 0.986)	1.286	0.917, (0.883, 0.951)	1.406
Uncontrolled crossing	0.86, (0.838, 0.882)	1.599	0.856, (0.834, 0.878)	1.613	0.835, (0.814, 0.856)	1.685	0.833, (0.812, 0.854)	1.691
Roundabout	1.103, (1.061, 1.148)	1.441	1.125, (1.082, 1.17)	1.500	0.98, (0.943, 1.018)	1.166	0.885, (0.853, 0.919)	1.511
Mini roundabout	0.771, (0.742, 0.802)	1.916	0.751, (0.723, 0.78)	1.995	0.694, (0.668, 0.721)	2.238	0.691, (0.666, 0.717)	2.252
Motorway junction	1.565, (1.442, 1.698)	2.506	1.446, (1.334, 1.567)	2.250	1.513, (1.395, 1.642)	2.395	1.583, (1.461, 1.715)	2.544
Traffic signal	0.626, (0.615, 0.638)	2.574	0.625, (0.614, 0.637)	2.577	0.626, (0.614, 0.637)	2.576	0.622, (0.611, 0.634)	2.595
Speed camera	0.778, (0.718, 0.842)	1.893	0.764, (0.706, 0.826)	1.946	0.747, (0.69, 0.808)	2.012	0.73, (0.674, 0.79)	2.083
Link bidirection	0.89, (0.873, 0.907)	1.496	0.784, (0.77, 0.8)	1.867	0.825, (0.809, 0.841)	1.720	0.936, (0.919, 0.952)	1.340

**Continuous treatment variables**	400 m		800 m		2 km		5 km	
	IRR, (95% CI)	E-value	IRR, (95% CI)	E-value	IRR, (95% CI)	E-value	IRR, (95% CI)	E-value
Link average width	1.129, (1.125, 1.132)	1.510	1.138, (1.134, 1.141)	1.534	1.11, (1.106, 1.113)	1.459	1.077, (1.074, 1.08)	1.365
Link length	1.104, (1.101, 1.108)	1.444	1.161, (1.159, 1.163)	1.593	1.191, (1.189, 1.193)	1.668	1.202, (1.2, 1.204)	1.696
Link angular curvature	0.717, (0.717, 0.717)	2.137	0.765, (0.765, 0.766)	1.939	0.754, (0.754, 0.755)	1.982	0.778, (0.778, 0.778)	1.891
Link degree	0.908, (0.906, 0.91)	1.435	0.838, (0.836, 0.841)	1.672	0.852, (0.849, 0.854)	1.627	0.865, (0.863, 0.867)	1.581
Connectivity	0.977, (0.974, 0.981)	1.178	0.863, (0.859, 0.867)	1.588	0.71, (0.707, 0.712)	2.168	0.782, (0.779, 0.784)	1.878
Betweenness	1.254, (1.248, 1.259)	1.818	1.431, (1.424, 1.438)	2.217	1.854, (1.845, 1.863)	3.112	1.882, (1.874, 1.89)	3.170
Closeness	1.087, (1.082, 1.092)	1.394	1.058, (1.053, 1.063)	1.307	0.974, (0.97, 0.978)	1.192	1.025, (1.02, 1.029)	1.184
Average shortest path	1.266, (1.26, 1.272)	1.847	1.168, (1.163, 1.172)	1.610	1.148, (1.144, 1.153)	1.561	1.142, (1.138, 1.147)	1.546
Diversion ratio	0.919, (0.915, 0.922)	1.399	0.897, (0.894, 0.901)	1.472	0.943, (0.94, 0.947)	1.312	1.021, (1.017, 1.025)	1.168

Note: Reported estimates are incidence rate ratios (IRR) and 95% confidence intervals obtained from post-adjustment negative binomial mixed-effects models fitted after propensity score adjustment. Binary treatments were adjusted using propensity score matching (PSM), and continuous treatments using generalized propensity score (GPS) weighting. All post-adjustment models incorporate matching or GPS-derived weights and city-level random intercepts. For binary treatments, the IRR represents the average treatment effect on the treated (ATT). For continuous treatments, the IRR corresponds to the multiplicative change in speeding associated with a one–standard-deviation increase in the standardized exposure. E-values quantify the minimum strength of association, on the risk-ratio scale, that an unmeasured confounder would need to have with both the treatment and the outcome, conditional on observed covariates, to fully explain away the estimated effect. These sensitivity measures are heuristic robustness diagnostics and do not constitute sharp bounds on causal effects. Results are reported across 400 m, 800 m, 2 km, and 5 km distance bands.

Table S22 Sensitivity analysis of post-adjustment causal effect estimates using E-values for unmeasured confounding in raw speeding events model

**Binary treatment variables**	400 m		800 m		2 km		5 km	
	IRR, (95% CI)	E-value	IRR, (95% CI)	E-value	IRR, (95% CI)	E-value	IRR, (95% CI)	E-value
Traffic calming	0.538, (0.526, 0.55)	3.125	0.541, (0.529, 0.553)	3.103	0.548, (0.536, 0.561)	3.050	0.575, (0.562, 0.588)	2.872
Choker	0.734, (0.672, 0.802)	2.065	0.85, (0.778, 0.929)	1.632	0.811, (0.742, 0.886)	1.769	0.836, (0.765, 0.913)	1.682
Traffic island	1.439, (1.396, 1.484)	2.235	1.365, (1.324, 1.408)	2.071	1.39, (1.348, 1.432)	2.126	1.387, (1.347, 1.429)	2.120
Signalised crossing	1.083, (1.054, 1.112)	1.382	1.092, (1.063, 1.122)	1.409	1.039, (1.012, 1.067)	1.241	1.024, (0.997, 1.051)	1.179
Marked crossing	1.013, (0.962, 1.066)	1.126	1.031, (0.98, 1.085)	1.209	1.094, (1.04, 1.151)	1.415	1.124, (1.069, 1.183)	1.498
Uncontrolled crossing	1.189, (1.148, 1.233)	1.664	1.184, (1.142, 1.227)	1.651	1.09, (1.052, 1.129)	1.402	1.074, (1.037, 1.112)	1.355
Roundabout	1.046, (0.986, 1.109)	1.266	1.026, (0.967, 1.089)	1.190	1.095, (1.035, 1.158)	1.417	1.129, (1.069, 1.192)	1.511
Mini roundabout	0.722, (0.684, 0.762)	2.116	0.729, (0.691, 0.77)	2.085	0.673, (0.639, 0.709)	2.336	0.672, (0.638, 0.708)	2.342
Motorway junction	1.221, (1.089, 1.368)	1.740	1.27, (1.138, 1.417)	1.855	1.292, (1.156, 1.444)	1.906	1.283, (1.15, 1.432)	1.885
Traffic signal	0.763, (0.743, 0.784)	1.947	0.789, (0.769, 0.811)	1.849	0.779, (0.759, 0.8)	1.886	0.793, (0.773, 0.814)	1.834
Speed camera	1.28, (1.131, 1.448)	1.878	1.22, (1.079, 1.379)	1.737	1.288, (1.135, 1.463)	1.898	1.242, (1.095, 1.41)	1.791
Link bidirection	0.357, (0.346, 0.368)	5.047	0.329, (0.319, 0.338)	5.535	0.508, (0.494, 0.523)	3.349	0.566, (0.552, 0.581)	2.930

**Continuous treatment variables**	400 m		800 m		2 km		5 km	
	IRR, (95% CI)	E-value	IRR, (95% CI)	E-value	IRR, (95% CI)	E-value	IRR, (95% CI)	E-value
Link average width	1.625, (1.617, 1.633)	2.633	1.643, (1.635, 1.652)	2.672	1.54, (1.533, 1.548)	2.452	1.399, (1.392, 1.406)	2.147
Link length	2.115, (2.106, 2.124)	3.651	2.183, (2.177, 2.189)	3.790	2.096, (2.091, 2.101)	3.612	2.079, (2.074, 2.084)	3.576
Link angular curvature	0.56, (0.56, 0.561)	2.969	0.633, (0.632, 0.633)	2.539	0.63, (0.63, 0.63)	2.553	0.647, (0.646, 0.647)	2.465
Link degree	1.003, (0.999, 1.007)	1.059	0.884, (0.881, 0.887)	1.516	0.913, (0.909, 0.916)	1.419	0.93, (0.927, 0.934)	1.358
Connectivity	0.852, (0.847, 0.856)	1.626	0.702, (0.698, 0.707)	2.201	0.581, (0.578, 0.584)	2.834	0.716, (0.712, 0.721)	2.140
Betweenness	1.154, (1.146, 1.163)	1.576	1.109, (1.101, 1.118)	1.458	1.924, (1.906, 1.941)	3.256	2.857, (2.833, 2.88)	5.160
Closeness	1.157, (1.148, 1.165)	1.582	1.035, (1.028, 1.042)	1.226	0.839, (0.834, 0.844)	1.670	0.842, (0.837, 0.848)	1.658
Average shortest path	1.314, (1.305, 1.322)	1.956	1.203, (1.196, 1.21)	1.697	1.126, (1.119, 1.132)	1.502	1.176, (1.169, 1.184)	1.632
Diversion ratio	0.897, (0.893, 0.901)	1.471	0.831, (0.827, 0.835)	1.697	0.928, (0.923, 0.933)	1.368	1.041, (1.035, 1.047)	1.249

Note: Reported estimates are incidence rate ratios (IRR) and 95% confidence intervals obtained from post-adjustment negative binomial mixed-effects models fitted after propensity score adjustment. Binary treatments were adjusted using propensity score matching (PSM), and continuous treatments using generalized propensity score (GPS) weighting. All post-adjustment models incorporate matching or GPS-derived weights and city-level random intercepts. For binary treatments, the IRR represents the average treatment effect on the treated (ATT). For continuous treatments, the IRR corresponds to the multiplicative change in speeding associated with a one–standard-deviation increase in the standardized exposure. E-values quantify the minimum strength of association, on the risk-ratio scale, that an unmeasured confounder would need to have with both the treatment and the outcome, conditional on observed covariates, to fully explain away the estimated effect. These sensitivity measures are heuristic robustness diagnostics and do not constitute sharp bounds on causal effects. Results are reported across 400 m, 800 m, 2 km, and 5 km distance bands.
